# The knowledge of danger signs of obstetric complications among women in rural India: evaluating an integrated microfinance and health literacy program

**DOI:** 10.1186/s12884-021-03563-5

**Published:** 2021-01-23

**Authors:** Danish Ahmad, Itismita Mohanty, Avishek Hazra, Theo Niyonsenga

**Affiliations:** 1 Health Research Institute, Faculty of Health, University of Canberra, Canberra, Australia; 2 Public Health Foundation of India, and Indian Institute of Public Health-Gandhinagar (IIPH-G), New Delhi and Gandhinagar, India; 3 Population Council, New Delhi, India

**Keywords:** Maternal health, Community health program, Microfinance and Self-help group, Health literacy, Obstetric complications, Maternal danger signs, Health diffusion

## Abstract

**Background:**

Maternal mortality can be prevented in low-income settings through early health care seeking during maternity complications. While health system reforms in India prioritised institutional deliveries, inadequate antenatal and postnatal services limit the knowledge of danger signs of obstetric complications to women, which delays the recognition of complications and seeking appropriate health care. Recently, a novel rapidly scalable community-based program combining maternal health literacy delivery through microfinance-based women-only self-help groups (SHG) was implemented in rural India. This study evaluates the impact of the integrated microfinance and health literacy (IMFHL) program on the knowledge of maternal danger signs in marginalised women from one of India’s most populated and poorer states - Uttar Pradesh. Additionally, the study evaluates the presence of a diffusion effect of the knowledge of maternal danger signs from SHG members receiving health literacy to non-members in program villages.

**Methods:**

Secondary data from the IMFHL program comprising 17,232 women from SHG and non-member households in rural Uttar Pradesh was included. Multivariate logistic regression models were used to identify the program’s effects on the knowledge of maternal danger signs adjusting for a comprehensive range of confounders at the individual, household, and community level.

**Results:**

SHG member women receiving health literacy were 27% more likely to know all danger signs as compared with SHG members only. Moreover, the results showed that the SHG network facilitates diffusion of knowledge of maternal danger signs from SHG members receiving health literacy to non-members in program villages. The study found that the magnitude of the program impact on outcome remained stable even after controlling for other confounding effects suggesting that the health message delivered through the program reaches all women uniformly irrespective of their socioeconomic and health system characteristics.

**Conclusions:**

The findings can guide community health programs and policy that seek to impact maternal health outcomes in low resource settings by demonstrating the differential impact of SHG alone and SHG plus health literacy on maternal danger sign knowledge.

**Supplementary Information:**

The online version contains supplementary material available at 10.1186/s12884-021-03563-5.

## Background

Improving maternal health is a global public health priority. While maternal health, essentially captured by estimates of maternal mortality showed a substantial decline during the Millennial Development Goals (MDG) period (2000–2015), desired global and country-specific goals for maternal mortality reduction were almost universally missed [1, 2]. Consequently, estimates from key studies suggested that despite the 39% reduction in maternal mortality ratio (MMR) over the MDG period, almost 295,000 maternal deaths still occurred annually in 2017 with the majority disproportionately situated in Africa and South Asia [2]. Moreover, the unmet gap in reducing maternal deaths from the MDG’s period has now carried over to the Sustainable Development Goals (SDG) with an ambitious target of maternal mortality ratio of 70 per 100,000 live births by 2030 for *all* countries [3].

Reducing maternal mortality in high burden regions requires addressing preventable causes of maternal mortality that may occur at any stage of maternity requiring high-quality person-centred care [4, 5]. These often manifest in pregnant women through physical signs related to underlying pregnancy-related complications, namely bleeding disorders, pregnancy-induced hypertension (eclampsia), delivery complications, post-delivery bleeding and infections [4, 6]. These physical signs act as an early warning or danger signs of maternal complications. Studies show that within Sub-Saharan Africa and South Asia, there is limited health system capability in providing emergency maternity care, that contributes to the overall high rates of maternal death [4, 7]. Therefore, achieving the maternal health SDG target would require novel strategies that complement existing country-level efforts, especially among low resource and high disease burden regions where substantial maternal deaths are avoidable [1, 4].

According to the World Health Organization (2019), the majority (99%) of maternal deaths still occur in low-income regions of South Asia and Sub-Saharan Africa where selected countries contribute the substantial burden [1, 4, 8, 9]. Within South Asia, India alone accounts for an estimated 10% of global maternal deaths or 45,000 maternal deaths annually and 20% of global under-5 child mortality with 1.04 million deaths estimated annually [1, 10, 11]. India accelerated the rate of maternal death decline in the latter half of the MDG period (2006–2015) due to strategic health system reforms that prioritised community health care and incentivised institutional delivery, leading to a national average of 80% institutional deliveries across rural and urban populations [10–12]. Importantly, the rise in institutional deliveries has not been matched with adequate provision of Basic and Emergency Medical Obstetric Care (EMOC) in facilities in rural areas [13]. Previous studies from India found that institutional deliveries alone, in the absence of high-quality EMOC and adequate referral system, is weakly associated with maternal mortality reduction [14–16]. Studies from other countries also showed that prioritising institutional deliveries alone, without adequate investments to ensure skilled high-quality care, increases the risk of negligence in maternal health care facilities [17, 18].

In India, substantial regional disparities account for select northern states traditionally reporting low development and maternal health indicators. Notably, the state of Uttar Pradesh (UP) accounts for the highest number of maternal deaths in India, which is partly attributed to the state population (200 million) [10, 19, 20]. The Maternal Mortality Ratio (MMR) in Uttar Pradesh stands at 188 per 100,000 live births compared to the national MMR of 130 per 100,000 live births [21]. Institutional delivery in rural regions in UP is substantially lower at 66% as compared to the national rural average of 75% [22, 23]. Importantly, UP reported 34% home deliveries in 2016, among which only 4 % were attended by a skilled birth attendant [23].

Studies from rural Uttar Pradesh showed that delays in care-seeking were exacerbated by health system gaps in the provision of adequate pre-and post-natal care, gaps that disproportionately impact poor families with low literacy [24–27]. Additionally, among the 22% of pregnant women in rural UP who reported receiving the minimum required three Antenatal care (ANC) visits (now the minimum required ANC visits are revised to four), only 10% consumed the recommended iron-folic acid (IFA) for 100 days or more when they were pregnant [23].

It was reported that less than 28% of women received basic post-natal care (PNC) in rural UP among which a meagre 17% received PNC within the first week of post-delivery where most complications are likely to occur [13]. Inadequate ANC and PNC compromise the quality of health literacy related to maternal complications recognition in women. Women who know the danger signs are likely to support earlier identification of maternal complications when they occur and prompt families to seek early health care. Studies in many settings found that low knowledge levels predispose women and households to either miss out potential maternal complications or seek delayed care to the detriment of the mother [16, 24, 27–29].

Pervasive cultural beliefs about the nature of the complications indicated that many women in rural settings lacked awareness of danger signs of maternal illness, which influences the decision-making process to seek care [16, 24]. Studies in rural UP showed that families with low access to maternal services were more likely to follow cultural traditions around the timing and place of childbirth [16, 24]. These traditions and social norms restrict women’s mobility and access to treatment during maternal complications [24, 29].

### Community-based health literacy and microfinance program

In rural India and similar regions, community-based health literacy interventions are used to supplement formal health system efforts to promote routine maternal health service utilisation related to antenatal visits, institutional deliveries and skilled attendance at birth [7, 29, 30]. Studies highlighted the positive effect of health literacy on improving maternal health utilisation despite low levels of education in communities [31]. Community health programs are increasingly providing evidence for reducing maternal and newborn health inequities in rural areas through women’s empowerment and support greater female economic participation [32–34]. Additionally, community mobilisation advocates progressively push for layering multiple interventions, including financial mechanisms in developmental packages for low resource regions seeking to impact maternal mortality [35–37]. Women only microfinance-based Self-help groups (SHG) program is a novel intervention to address informational and financial barriers to maternal care-seeking by improving maternal health literacy and providing access to credit [33, 38]. The provision of health knowledge through peer-network of SHGs is also postulated to shift social norms about maternal health outcomes by providing an enabling environment for discussion and change [39–41]. Moreover, SHGs have now been placed under India’s rural health programs facilitating coordination of health services with community health workers in villages [12, 42]. SHGs as a developmental model permits the scaling of an added health intervention and allows the diffusion of new knowledge and skills through program channels over time in a social system [39, 43].

The SHG platform is the underlying developmental model of the IMFHL program evaluated in this paper. The IMFHL program aimed to empower marginalised women by organising them in self-help groups comprising 10–15 households to adopt desired health behaviours and provide access credit for poverty reduction [44, 45]. Studies have shown that SHG membership fosters social capital and cohesion among member through group collectivisation, with membership influencing social norms and behaviours [39–41]. Previous studies in rural India suggested that SHG membership may increase access to social and health advice networks for mothers, including increased linkages to the health system [39, 40]. However, the potential of SHGs as a community network to reach non-members with new knowledge has not been studied.

While most studies attribute the change in health behaviours due to the influence of program input, limited studies have explored the phenomenon of diffusion of health literacy from program members to non-members [39, 44, 46]. Diffusion in this paper adopts a definition that explains the process by which an innovation - in this case, the knowledge of maternal danger signs, is communicated through programmatic channels over time from members to non-members of a social system [43]. Moreover, in this study, diffusion is contrasted with dissemination which entails active planned efforts to reach an intended outcome [47]. For example, the layering of a health literacy component on the SHG platform is indicative of a planned approach for the dissemination of health information among members; whereas the assumed natural transfer of knowledge from SHG members to neighbouring non-members without planned programmatic input reflects the phenomenon of diffusion.

The commonly adopted Roger’s model of diffusion has been used in public health programs to describe the pattern of behaviour change adoption across communities using selected interventions such as contraceptive use, child marriage and intimate partner violence [47–50]. Moreover, while previous diffusion studies have shown the key role of interpersonal connection in promoting health information, the application of the model to evaluate the spread of microfinance has not yet been done [32, 39, 44, 50]. Particularly, no study elsewhere, to the best of our knowledge, has evaluated the diffusion of the knowledge of maternal danger signs from microfinance members to non-members in a rural setting with low literacy and high poverty- populations.

The IMFHL program provided health literacy to women on recognising key pregnancy-related danger signs, and adoption of birth preparedness complication readiness (BPCR) plans to reduce delays in seeking health care during maternal complications. While studies evaluating SHGs and embedded health programs have demonstrated the gain in knowledge of routine maternal services such as antenatal care and institutional delivery [32, 36, 44, 51], no study till date examined the incremental impact of program participation on knowledge of maternal danger signs among women or the women’s likelihood of identifying the risks of complications during the pregnancy, delivery and post-partum period. Moreover, there is limited evidence about the effects of broader individual, household, community, and area-level confounders on knowledge of danger signs among members and non-members of integrated microfinance and health literacy (IMFHL) program in rural settings.

This research aims to evaluate if membership in an IMFHL program improves the knowledge levels of maternal danger signs associated with high-risk pregnancies among women in rural Uttar Pradesh while adjusting for other individual, households and community or area-level characteristics. This research also seeks to investigate the impact of exposure to each of the different levels of the integrated program and the presence of a diffusion effect of knowledge from member to non-member households in program villages in Uttar Pradesh. The study hypothesises that providing health literacy to women through the SHG program is likely to increase knowledge of maternal danger signs leading to early recognition of danger signs at home and fewer delays in seeking health treatment, and thereby improving maternal health.

**Fig. 1:**
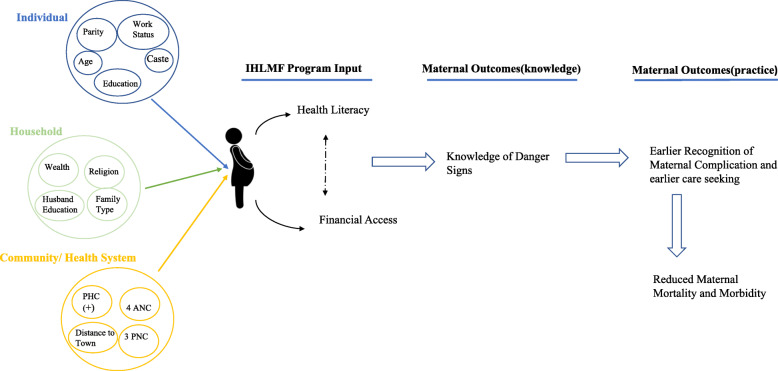
Analysis framework Program Main and Secondary effect on Improving the Knowledge of Maternal Danger signs (outcome) among women in Rural Uttar Pradesh, India. Legends: PHC+ (Primary Health Center present in community); ANC (Ante Natal Care); PNC (Post Natal Care)

Figure 1 provides a conceptual framework showing the program’s input on eligible woman’s knowledge of maternal danger signs and hypothesized improvement in care-seeking practises.

## Methods

### Study design and study setting

This paper uses secondary data collected from 17,232 women from an Integrated microfinance and health literacy (IMFHL) program that was implemented in rural Uttar Pradesh, India between 2012 and 2017 (please see [52] for a detail description of this survey). The program aimed to provide low-income women with maternal and newborn health literacy delivered through a microfinance platform [38, 48, 52]. Under the program, a quasi-experimental survey design was used to collect cross-sectional survey data in two rounds (round 1 in 2015; round 2 in 2017) to evaluate the program’s impact on knowledge and health behaviours of women during pregnancy, delivery and post-delivery in Uttar Pradesh [38, 48, 52].

### The IMFHL program

**Fig. 2:**
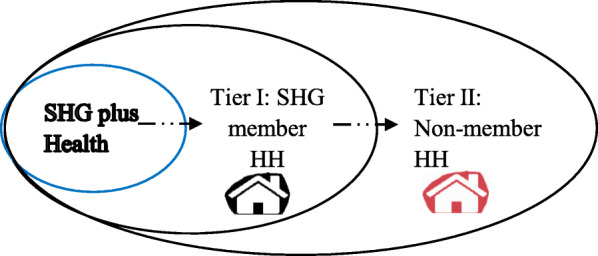
The Direction of Potential Knowledge Diffusion from member households (tier I) to neighbouring non-member households (tier II) in the same village

The IMFHL program was a community-driven and rapidly scalable program that integrated health promotion activities in a microfinance platform across low developmental districts of UP [44, 52]. Details of the IMFHL implementationand health intervention have been published elsewhere [44, 52]. The health promotion component sought to address community-related barriers mainly related to low health literacy and poverty and to encourage women to adopt preventive health behaviours known to reduce maternal and neonatal mortality [44, 52]. The IMFHL program was built on previous participatory community programs such as the Makwanpur trial in Nepal and the Shivgarh trial in Uttar Pradesh that showed a reduction in maternal and newborn mortality achieved through the adoption of essential maternal and newborn care practices in households [29, 53]. Under the IMFHL program, the maternal health literacy component targeted eligible women at different stages of their pregnancy and provided them with information to recognise pregnancy-related complication signs in order to reduce delays in seeking care from a health facility in the event of any pregnancy-related complication [38, 44]. A SHG member, trained as a health volunteer, facilitated health discussions involving pregnant and recently delivered women using key program strategies such invitation to SHG meetings, reminder letters to pregnant and new mothers with key health messages, house visits, and exposure to community health video shows developed by the program [44, 52]. Furthermore, the IMFHL program created three apparent groups of beneficiaries. The baseline tier was composed of pregnant women in SHG member households that received health messages directly through program strategies (IMFHL intervention group). Since villages are comprised of member and non-member households living in close proximity and communicating with each other, a process of community-based diffusion in knowledge sharing was expected from SHG plus Health (IMFHL intervention group) to tier I and to tier II households respectively, to supplement direct program intervention efforts as depicted in Fig. 2.

Diffusion of knowledge was expected to occur in SHG program villages through a process of collective socialisation in which heath literate SHG members serve as role models and help other non-members internalise biomedical norms around pregnancy and childbirth [30–41]. This research examined the above-stated assumption to determine if the SHG platform encourages the sharing of health information from members receiving health literacy to non-members.

### IMFHL program implementation: selection of intervention and comparison blocks

Under the IMFHL program, implementation districts were selected comprising high maternal and neonatal mortality burden with a higher percentage of scheduled caste (SC)/tribe (ST) and low literacy [52]. SC/ST are members from communities designated by the Indian Government to have historically faced ‘extreme social, educational and economic backwardness arising out of the traditional practice of untouchability’ and afforded legislative protection and entitlements [54].

Within districts, 120 implementation blocks were selected and separately,83 comparison blocks, were roughly matched to the intervention blocks as per the percentage of SC/ST [38, 44], Lastly, ‘pure control’ blocks that received no SHG or health intervention were chosen to observe secular change in health indicators in rural Uttar Pradesh. Eligible women in households were identified as SHG members in villages using a community participatory approach and inclusion criteria [52]. The most disadvantaged households were often represented by landless poor households with low literacy, lower social class (and caste) with multiple (social) deprivations [44, 52]. In these villages, one woman from an eligible household was allowed to join SHGs that were nurtured by the field staff. Other households who would not be facing similar credit constraints as poorer households from lower castes in the same villages, were not targeted by the SHG program for membership [44, 52].

### Survey sampling approach and study population

#### Sampling strategy for survey

With a total population close to 200 million, Uttar Pradesh state is administratively subdivided into 75 districts, 822 blocks and 98,000 Gram panchayats (GP) [52]. The IMFHL program was implemented in 203 blocks while the survey data was collected from 70 blocks in 20 districts as a representative sample from the IMFHL program’s coverage area [44, 52]. In India, GPs are the smallest unit of administration within blocks where SHGs were established. Where data were collected, a GP may be classified as a larger main village with smaller peripheral villages attached to it and may have fewer houses attached as hamlets. The surveys followed a three-stage sampling approach for selecting blocks, GPs and finally, households, based on the state’s administrative hierarchy, and as depicted in Fig. 2 below.

**Fig. 3:**
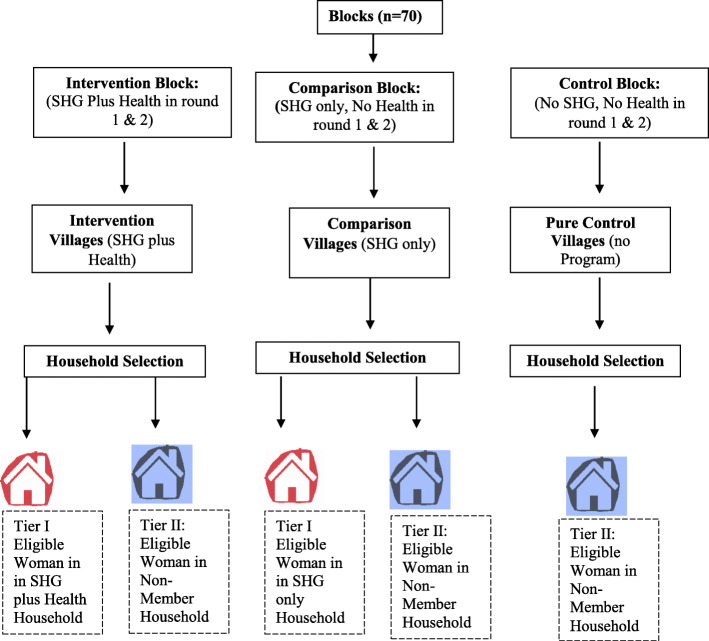
Multi-stage sampling approach comprising block, village, and household selection

The IMFHL program collected data in both survey rounds from three types of blocks, depending on the IMFHL program exposure: i) **intervention** blocks where households received health intervention through SHG program, ii) **comparison** blocks where households received SHG program only, and iii) **pure control** blocks where households did not receive any program exposure reflecting the natural change in health indicators. While the IMFHL program used SHGs in **both intervention and comparison** blocks, only households in intervention blocks received additional health intervention (see Fig. 3). Moreover, both intervention and comparison blocks had SHG membership of similar duration (average duration of 18 months). In the first stage, the intervention (SHG plus health) blocks were first arranged in ascending order of their associated percentage of Scheduled Caste (SC) and Scheduled Tribe (ST) population (SC/ST), a critical parameter for development [54]. The required number of intervention blocks were then equally selected by random sampling within each SC/ST-based stratum [38, 44]. Comparison (SHG only) blocks were selected within the same district (or from a geographically adjacent district if comparison block were not available in the same district) to reduce the effect of socio-cultural diversity between study blocks [38, 44]. Although comparison blocks comprised of roughly similar proportions of SC/ST as intervention blocks, these comparison blocks were, however, not one-to-one matched pairs and were selected independently of the intervention blocks [44, 52]. The average proportion of SC/ST population in the intervention and comparison blocks were similar (45% and 44%). Lastly, pure control blocks (no SHG and no health intervention) were also selected based on block percentage of SC/ST as the criterion for matching with intervention and comparison blocks in the same districts [44, 52].

In the second stage, Gram Panchayats (GPs) were selected within comparison and intervention blocks as per SHG population coverage, and village population size in pure control blocks as no SHGs were established in these blocks [44, 52]. In intervention and comparison blocks where SHGs had been established, GP’s were drawn in equal numbers from three strata of SHG coverage: 5–15%, 16–30% and 30–60%. Outlier GPs with coverage of SHGs < 5% and > 60% were excluded [44, 52]. Whereas GPs in pure control blocks (no SHG, no health) were selected based on GP population size and with a similar proportion of main village and hamlets as comparison arm (with SHG, no health intervention) to ensure similar population characteristics in these villages [44, 52].

In the final stage, households were selected from all three categories of blocks following a house listing and mapping exercise to develop a sampling frame to identify the eligible woman in intervention and comparison block [44, 52]. While eligible women in SHG member and non-member households were selected in intervention and comparison blocks, only eligible women from non-member households were selected in pure control blocks [44, 52]. The listing and mapping exercise in intervention and comparison blocks showed that the number of SHG households with an eligible woman was almost equal to the sample size requirement; therefore, all SHG households with eligible women were selected for an interview in these blocks [38, 44].

As SHG programs enrol one member from each household only, each individual woman in the survey represents a household; a random procedure was used to select the eligible respondent where more than one eligible woman was found in a household [44, 52]. Furthermore, the house listing and mapping exercise in pure control blocks also provided a sampling frame to select non-member households following a systematic random sampling [44, 52].

In the successive survey rounds, data was collected from the same GPs, but not the same households or women [44, 52]. Furthermore, as the survey used different selection criteria at the higher level (stratified and matched block selection using SC/ST) and at a lower level (stratified GP selection based on population coverage by SHGs members and non-members), this survey analyses all eligible women in households across the sampled GPs.

### Data collection

Separate questionnaires were used to collect individual, household and village data. The eligible respondents comprised currently married women aged 15 to 49 years who had had a baby in the 12 months preceding the survey; household head; and village representatives. Trained data collectors administered interviews in the local language (Hindi) after obtaining verbal informed consent from respondents using computer-assisted personal interview (CAPI) package designed in the Census and Survey Processing System (CSPro), a public domain software used for census and survey data [44, 52].

Data collection from eligible women used a structured survey questionnaire with open-ended questions. Knowledge of danger signs was spontaneously recalled by women and then marked against danger signs options in the questionnaire. The questionnaire separately elicited probed responses for danger signs, however, for the purposes of this research paper only spontaneously provided responses by the woman were considered. The survey instrument is provided [see Additional file 1].

To capture the individual, households, and community/area level influence on maternal danger signs among member and non-member women, this analysis merged individual-level, household, and village level sub-datasets across rounds.

### Outcome and explanatory variables

i) **Outcome variable:** The main outcome variable captured eligible woman’s self-reported knowledge of maternal danger signs. A binary variable capturing no or partial knowledge (=0), and complete knowledge of danger signs (=1) was created for this study.

In the IMFHL program, the survey collected data from women on self- recalled knowledge of key danger signs in the last pregnancy retrospectively. In both rounds, eligible women irrespective of place of delivery were asked to recall multiple responses to the question “*What problems/complications can a woman face during pregnancy or delivery or within 42 days of delivery which require immediate medical attention?”* The responses in the interview were marked against fourteen common medical danger signs occurring across the maternity period, such as severe headache, blurred vision, loss of foetal movement etcetera. As some of the fourteen signs also comprised signs that were common to normal pregnancy, a clinical review was undertaken by a medical doctor for this study to select key danger signs indicative of serious direct (preventable) causes of maternal mortality for this analysis. The dangers signs selected identify complications in pregnancy, delivery and the post-partum period that are associated with main direct causes of mortality in India and other high maternal burden countries namely haemorrhage, eclampsia, severe maternal infections, prolonged labour.

ii) **Main Explanatory variables:** The main explanatory variable, the IMFHL intervention, was categorised into five levels based on household’s exposure to IMFHL program. An ordinal variable was created to capture the program’s main effect on women’s knowledge, that is, the change in women’s knowledge across the program levels of exposure: intervention (SHG plus health), comparison (SHG only) and control (no SHG, no health) villages. Woman’s SHG membership is determined by the eligible woman being *herself* a member of the SHG or *belonging* to a household where someone else (e.g., mother, mother-in-law, sister in law) is an SHG member. The coding of the IMFHL intervention variable with description is shown as follows:

- Group 0: Comprised of households that were not SHG members (non-members) and were in villages without *any* program intervention (pure control households).
- Group 1: Comprised of households that were not SHG members (non-members) but were in program villages where the SHG program alone was implemented (diffusion-control households).
- Group 2: Comprised of households that were not SHG members (non-members) but were in program villages where the SHG program plus Health intervention was implemented (diffusion-control households).
- Group 3: Comprised of households that were SHG members in program villages where only SHG program was implemented (comparison households).
- Group 4: Comprised of households that were also SHG members but were in villages where both the SHG program and additional health intervention was provided. Only these households received health intervention through the SHG (intervention households).

iii) A survey round variable was created to assess the effect of program intervention on women in round II (2017) compared to round I (2015).
iv) **Confounding Variables:** The analysis included a comprehensive set of confounding variables that were identified from the maternal health literature and captured at the individual and community levels. They represented socio-demographic, health, and community factors. The *individual maternal health variables* included parity, history of past pregnancy-related complication, number of previous pregnancy loss, knowledge of minimum ANC visits required, and place of last delivery. Whereas *maternal health system utilisation variables* were the quality and number of ANC received, duration of stay in a hospital after delivery and intensity of contact with the frontline worker in last pregnancy. *Socio-demographic variables* represented the type of family (nuclear versus joint/extended), household head’s religion, social caste, and the years of education attained by an eligible woman and her husband. *Economic variables* capturing household poverty included the working status of eligible woman and a composite variable of household wealth quintile. The wealth quintile variable was constructed for this analysis using Polychoric Principal Component Analysis (PCA) combining household assets and amenities to evaluate program impact across five gradients of poverty from the poor (reference category) to the poorest.

### Sample size

Under the IMFHL program, the total sample size was calculated considering a seven percentage point increase in primary health indicators, for example, institutional delivery, number of antenatal care visits and others, after program implementation, with an 85% power to detect changes, the usual 5% level of significance and a design effect of 2 [44, 52]. In this study, a sample comprising 17,232 eligible women was used, out of which 41% or 7144 women were SHG members.

### Statistical data analyses and models

This study examined the effect of an IMFHL intervention on knowledge levels of maternal danger signs in women from households that were either SHG members or non-members in rural Uttar Pradesh.

The dependent variable in this analysis was a binary variable that represents the knowledge of maternal danger signs about obstetric complications among women. Consequently, the program impact was evaluated using multivariable logit regressions to establish the program’s effect while controlling for other confounders in models. While model I established the main effects of both the intervention and the survey round, model II included an interaction term to draw out the interaction effect of program exposure with survey round. Confounders related to individual health and health system were included in model III, while the full model IV included sociodemographic-economic and area-level variables. The results are reported as Odds ratios (OR) with associated 95% confidence intervals, and the model of best fit was evaluated using the Consistent Akaike Information Criteria (CAIC) and Bayesian Information Criteria (BIC), two valid measures of model fit, parsimony and model selection [55]. Estimates of effects were reported with associated 95% confidence intervals as suggested by NEJM guidelines [56]. The a priori level of significance was set at the usual 5% alpha and all *p*-values reported in Tables using the asterisk convention: ***: *p* < 0.01; **: *p* < 0.05; *: *p* < 0.10 [57], with the last category meant to show that a “trend towards statistical significance” has to be noted [58–60]. All analyses were performed using Stata 16 (Statacorp, USA).

### Summary statistics

Table 1 presents the summary statistics of the outcome variable used in this analysis.

**Table 1:** Knowledge about Maternal Danger Signs of Obstetric Complications Among Women (Aged 15–49) during their last pregnancy.Study population (*N* = 17,232)

Sl.no	Danger signs in Pregnancy/ Delivery & Post Delivery	Non-member Households n (%)	SHG Households n (%)
		10,088 (59%)	7144 (41%)
1	**During Pregnancy**		
	a. Severe headache /High blood pressure	964(10%)	656(9%)
	b. Blurred vision/Convulsions	412(4%)	267(4%)
	c. Absence or /less movements of foetus	478(5%)	376(5%)
2.	**During Labour and Delivery**		
	a. Prolonged labour over 12 h	181(2%)	129(2%)
	b. Excessive vaginal bleeding	1076(11%)	1037(15%)
	c. Delay in placental expulsion/Retained placenta	49(1%)	23(1%)
	d. Severe abdominal pain	3732(37%)	2325(33%)
	e. Rupture uterus	81(1%)	59(1%)
	f. Baby in abnormal position	730(7%)	606(8%)
	g. Cord prolapsed/Baby’s hand & feet coming out first	35(1%)	29(1%)
	h. Cord around neck	40 (1%)	27 (1%)
**3**	**Post-partum**		
	a. High fever	569 (6%)	384 (5%)
	b. Foul-smelling vaginal discharge	311 (3%)	319 (4%)
	c. Other (specify)	177 (2%)	145 (2%)
	d. Do not know	1253 (12%)	762 (11%)
**4.**	**Knowledge of Danger Signs across Pregnancy Phases**		
	• No knowledge of any danger signs	1325 (13%)	809 (11%)
	• Knowledge of danger signs either during pregnancy or labour/delivery or postpartum	346 (3%)	220 (3%)
	• Knowledge of danger signs during pregnancy and labour/delivery and postpartum	6924 (69%)	5242 (73%)

**Note**: Recalls under 1% have been rounded to 1%, the total, therefore, may be > 100%

A detail list of the knowledge of all danger signs that were retrospectively collected from eligible women is provided in Table 1, which shows the frequency and percentage of danger signs across different phases of pregnancy/delivery and 42 days post-partum. The table, categorised across member and non-member households showed that most women (37%) recalled severe abdominal pain, while only a minority (1%) recalled danger signs related to placental expulsion or umbilical cord issues. Moreover, the distribution of danger signs showed that women were more likely to recall those danger signs that occurred in the pregnancy and delivery period as compared to the post-partum period. The Table 1 also shows that across member and non-member households, the majority (> 69%) of women knew danger signs in all three phases of pregnancy, delivery and post-delivery with only a minority (< 15%) of all women reporting no knowledge.

Table 2 presents the summary statistics of the explanatory variables for eligible women categorised across (SHG) member and non-member households that capture all type of households’ program exposure and associated characteristics and relevant factors (individual and community levels).

**Table 2:** Summary statistics of variables by the Non-Self-Help Group [Non-member] Household and Self-Help Group [SHG] Households with *n* = 17,232 eligible women

	Variable	Summary Statistics (***N*** = 17,232)	
		Non-member Households n (%)	SHG Households n (%)
	***Independent Variables: Program Exposure Characteristics***		
1.	**Level of Household (HH) Microfinance (MF) Exposure**	10,088 (59%)	7144 (41%)
	HH in a village with **no SHG, no health** intervention (Pure control-reference)	3705 (36%)	–
	**Non-Member** Household in a village with **SHG only program**	3344 (33%)	–
	**Non-Member** Household in a village with **SHG plus health** intervention	3039 (30%)	–
	**SHG: Member** Household in a village with **SHG only intervention**	–	3622 (51%)
	**SHG plus Health: Member** Household in a village with **SHG** ***plus*** **Health** intervention	–	3522 (49%)
2.	**Evaluation Survey Round**		
	Round 1/ 2015 (=0)	5454 (54%)	3269 (45%)
	Round 2/−2017 (=1)	4634 (45%)	3875 (54%)
	***Independent Variables: Individual Health and Health System Characteristics***		
3.	**Parity (number of previous pregnancies) of Eligible Woman**	Mean = 2.4 (SD = 1.4)	Mean = 2.4 (SD = 1.4)
4.	**Any Past Pregnancy Loss (due to spontaneous/induced abortion)**	2548(25%)	1888(26%)
5.	**Any Complication experienced in last pregnancy/labour or post-partum**	4779 (47%)	3435 (48%)
6.	**Eligible woman’s correct knowledge of the minimum number of ANC required during pregnancy**	3714 (37%)	2760 (39%)
7.	**Received Four or more Antenatal Check-up (ANC) in last pregnancy with urine/blood pressure /weight/abdominal/ultrasound test in last ANC)**	2666 (26%)	2077 (29%)
8.	**Place of Last Delivery**		
	Home Delivery (reference)	1739 (17%)	1198 (17%)
	Institutional Delivery	8349 (83%)	5946 (83%)
9.	**Duration (in hours) of stay in Health Facility immediately after delivery**		
	1. Home Delivery (reference)	1739(17%)	1198(17%)
	2. Discharged within 12 h	5596(56%)	4125(58%)
	3. Discharged between 12 and 24 h	1134(11%)	795(11%)
	4. Discharged between 24 and 48 h	719(7%)	506(7%)
	5. Discharged between 48 and 72 h	283(3%)	146(2%)
	6. Discharged after > 72 h	617(6%)	374(5%)
10.	**Received 3 PNC in first 7 Days after Delivery**		
	Not Received	9180 (91%)	6485 (91%)
	Received 3 PNC in first 7 Days after Delivery	908(9%)	659(%)
11.	**Number of contact with ASHA/ANM/AWW/SHG in last pregnancy**	Mean = 4.0 (SD = 5.4)	Mean = 4.23 (SD = 5.4)
12.	**Distance (Kms) to Primary health Centre if not available in the village**	Mean = 5.4 (SD = 4.9)	Mean = 5.4 (SD = 4.7)
	***Independent Variables: Sociodemographic/Economic, and Area Level Characteristics***		
13.	**Distance (Kms) to closest town**	Mean = 1.47 (SD = 0.79)	Mean = 1.49 (SD = 0.75)
14.	**Population of Village**	Mean = 5153 (SD = 5135)	Mean = 5140 (SD = 5114)
15.	**Household (HH) with Below Poverty Line (BPL) Card**		
	HH without BPL Card -reference	5590(55%)	3830(54%)
	HH with BPL Card	4498(45%)	3314(46%)
16.	**Household Wealth Quintile (Poor to Poorest)**		
	1. Marginally Poor	1982(20%)	1498(21%)
	2. Moderately Poor	2046(20%)	1502(21%)
	3. Poor	1992(20%)	1469(20%)
	4. Poorer	2029(20%)	1395(20%)
	5. Poorest	2039(20%)	1280(18%)
17.	**Eligible Woman Presently Working to earn in cash, in kind or both**		
	Not Working	8427(53%)	5935(83%)
	presently working	1661(16%)	1209(17%)
18.	**Family Type**		
	Nuclear (reference)	4274 (42%)	2886 (40%)
	Joint and Extended Family	5814 (58%)	4258 (60%)
19.	**Religion**		
	Muslim (reference)	815 (8%)	601 (8%)
	Hinduism and Others	9273 (92%)	6543 (92%)
20.	**Scheduled Caste**		
	General Caste	1247 (12%)	873 (12%)
	Other Backward Caste	4358 (43%)	3072 (43%)
	Scheduled Caste and Scheduled Tribe	4483 (45%)	3199 (45%)
21.	**Eligible Woman (EW) Age in completed years**	Mean = 25 (SD = 4.56)	Mean = 25 (SD = 4.55)
22.	**Eligible Woman’s (EW’s) Education Level**		
	No schooling (reference)	3415 (34%)	2347 (33%)
	Completed Primary/Middle School (up to year nine) and above	6673 (66%)	4797 (67%)
23.	**EW’s Husband Education Level**		
	No schooling (reference)	1731 (17%)	1209 (17%)
	Completed Primary/Middle School (up to year nine) and above	8357 (83%)	5935 (83%)
	*Dependent Variable*		
24.	**Eligible Woman’s Knowledge of Maternal Danger sign**	10,088 (59%)	7144 (41%)
	No knowledge or partial knowledge (reference)	3164 (31%)	1902 (27%)
	Full Knowledge of Danger signs (in pregnancy/delivery/post-delivery up to 42 days)	6924 (69%)	5242 (73%)

**Acronyms:** Eligible woman (EW), Accredited Social Health Activist (ASHA)/Auxiliary Nurse Midwife (ANM) Anganwadi worker (AWW)-ASHA/ANM &AWW are Government health workers situated in villages as per population guidelines and provide preventative maternal, child and other health services

The sampled women in this analysis comprised of more women that were non-member (59%) than SHG members. However, an almost equal proportion of women spread across both survey rounds, and a proportionate number of households were allocated *within* each level of program exposure.

In both groups, eligible women reported a mean parity close to 2.4 reflecting near current Indian fertility rates (median 2.2, range 2.1–4), and among them, close to 50% women experienced an obstetric complication in their last pregnancy/delivery or post-partum period Furthermore, a quarter of all women across both groups reported experiencing a pregnancy loss either due to induced or spontaneous abortion. Although 83% women reported delivering in an institution for their last pregnancy (public or private), only a minority (26%) had received the minimum four antenatal care (ANC) visits in their last pregnancy with all required tests done in the last check-up, that are: urine, blood pressure, weight, abdominal and ultrasound tests. The summary statistics showed that among those women with an institutional delivery, the majority (70%) were discharged within 12 h after delivery which is less than the recommended 48 h stay post-delivery for normal deliveries [61]. Furthermore, only 10% of total women reported receiving the recommended minimum of three post-natal care visits within the crucial first 7 days post-delivery when maternal and neonatal complications commonly occur.

The villages were on average 5.4 km (km) from the closest Primary Health Centre (PHC) and about 1.47 km to the closet town.

The summary statistics reflecting household’s economic status showed that only 16% of women reported they were working to earn in cash or kind at the time of the survey, while almost 45% belonged to households that were living below the poverty line -an income-based measure of household poverty.

Moreover, most women (60%) were living in joint/extended households which is common in rural Uttar Pradesh. Almost 92% of all women identified themselves as being part of Hindu households, the majority religion in Uttar Pradesh and India. Moreover, 45% of women reported belonging to Scheduled Caste and Tribes (SC/ST), a proxy for social margination in the rural Indian content. Women across member and non-member households were comparable in relation to age (mean 26 years), and level of education. While 66% of all women interviewed reported having some level of education, the summary statistics showed that husbands overall were more likely (88%) to have had received some level of schooling. For the dependent variable, knowledge of danger signs in women, most women (70%) had full knowledge of danger signs in all phases of pregnancy/delivery and post-delivery.

### Inferential results: multivariable logistic regression models

Table 3 presents results from the multiple logistic regression models I and II of knowledge on Maternal Danger Signs of Obstetric Complications among women (Aged 15–49) during their last pregnancy categorised across SHG member and non-member households. Results, showing the main effect of program exposure across the survey rounds. The results from models III and IV are presented in Table 4, showing the additional effects of socio-demographic, economic and area-level confounders on the outcome in the adjusted models. The individual model coefficients were estimated using the maximum likelihood estimation method, and the Odds Ratios (OR), as measures of the magnitude of effects were reported along with their associated 95% confidence intervals (95% CI) and *p* values. Overall, the main findings from Table 3 showed an improvement in knowledge levels among SHG members receiving health literacy and even among non-members in the same villages due to a diffusion effect. Table 4 overall shows the consistency of program effect in the presence of confounders.

**Table 3:** Results of the logistic regression models I and II Estimating the Knowledge of Danger Signs about Obstetric Complication; Odds Ratio (OR) and associated 95% confidence intervals are reported: OR (95%CI)

		Main Effects	
S.no	Explanatory Variable name	Model I	Model II
		Odds Ratio (95% CI)	Odds Ratio (95% CI)
	**Main Effects**		
1.	**Level of Household (HH) Microfinance (MF) Exposure**		
	HH in a village with **no program** intervention (Pure control-reference)	–	–
	**Non-Member** Household in village with **SHG or SHG + health program**	0.81*** (0.74–0.89)	0.61*** (0.53–0.70)
	**SHG: Member** Household in village with **SHG only intervention**	1.08 (0.98–1.20)	1.06 (0.89–1.25)
	**SHG+ Health: Member** Household in village with **SHG** ***plus*** **Health** intervention	1.27*** (1.15–1.41)	0.80*** (0.68–0.95)
2	**Round**		
	Round 1 (reference)		
	Round 2	0.52*** (0.49–0.56)	0.37*** (0.32–0.43)
2.	**Two-way Interaction Effects: Round** # **Households (HH) Microfinance (MF) Exposure**		
	Round 1# Non-MF HH in Pure Control village (reference)		
	Round 2 # Non-MF HH in a village with MF or MF plus Health	–	1.65*** (1.37–1.98)
	Round2 # **SHG Member** HH in village with MF program only	–	1.05 0.85–1.30
	Round2 # **SHG+ Health Member** HH in village with MF plus health intervention	–	2.14*** (1.73–2.65)

**Note:** Confidence intervals in parentheses; and significant p-value showing ***: *p* < 0.01, **: *p* < 0.05 and *: *p* < 0.10 levels

**Table 4:** Results of the logistic regression models III and IV Estimating the Knowledge of Danger Signs about Obstetric Complication using Confounders; Odds Ratio (OR) and associated 95% confidence intervals are reported: OR (95%CI)

		Secondary Effects Using confounders	
S.no	Explanatory Variable name	Model III	Model IV
		Odds Ratio (95% CI)	Odds Ratio (95% CI)
1.	**Level of Household (HH) Microfinance (MF) Exposure**		
	HH in a village with **no program** intervention (Pure control-reference)		
	**Non-Member** Household in a village with **SHG or SHG + health program**	0.62*** (0.54–0.71)	0.61*** (0.53–0.71)
	**(SHG) Member** Household in a village with **SHG only intervention**	1.06 (0.90–1.26)	1.06 (0.90–1.27)
	**(SHG + Health) Member** Household in a village with **SHG** ***plus*** **Health** intervention	0.81*** (0.68–0.96	0.81*** (0.68–0.96)
2	**Round**		
	Round 1 (reference)		
	Round 2	0.35*** (0.30–0.41)	0.29*** (0.24–0.34)
3.	**Two-way Interaction Effects (Round** # **SHG Exposure)**		
	Round 1# Non-MF HH in Pure Control village (reference)		
	Round 2 # Non-MF HH in village with MF or MF+ Health	1.64*** (1.37–1.97)	1.65*** (1.37–1.99)
	Round2 # MF-HH in village with MF only	1.04 (0.84–1.29)	1.03 (0.83–1.28)
	Round2 # MF+ Health HH in village with MF plus health	2.13*** (1.72–2.64)	2.13*** (1.71–2.64)
	***Model III-Individual Health and Health System variables***		
4.	**Parity (number of previous pregnancies) of Eligible Woman**	0.96*** (0.93–0.98)	0.99 (0.96–1.02)
5.	**Any Past Pregnancy Loss (due to spontaneous/induced abortion)**	0.97 (0.90–1.05)	0.98 (0.91–1.06)
6.	**Any Complication experienced in last pregnancy/labour or post-partum**	0.84*** (0.78–0.90)	0.84*** (0.78–0.90)
7.	**Eligible woman’s knowledge of the minimum number of Antenatal Check-up (ANC) required during pregnancy**	1.03 (0.96–1.10)	0.98 (0.92–1.06)
8.	**Received four or more ANC in last pregnancy with urine/blood pressure /weight/abdominal/ultrasound test in last ANC**	1.01 (0.92–1.11)	0.92* (0.83–1.00)
9.	**Place of Last Delivery**		
	Home Delivery (reference)		
	Institutional Delivery	1.07 (0.90–1.28)	0.93 (0.78–1.10)
10.	**Duration (in hours) of stay in Health Facility immediately after delivery**		
	1. Home Delivery (reference)		
	2. Discharged within 12 h	1.22*** (1.03–1.43)	1.28*** (1.08–1.51)
	3. Discharged between 12 and 24 h	1.12 (0.93–1.35)	1.19* (0.99–1.43)
	4. Discharged between 24 and 48 h	1.03 (0.85–1.25)	1.08 (0.89–1.32)
	5. Discharged between 48 and 72 h	0.90 (0.69–1.16)	0.97 (0.74–1.26)
	6. Discharged after > 72 h	1.00	1.00
11.	**Received 3 PNC in first 7 Days after Delivery**	1.05 (0.93–1.19)	0.99 (0.88–1.12)
12.	**Number of contact with ASHA/ANM/AWW/SHG in last pregnancy**	0.99*** (0.98–1.00)	0.99*** (0.99–1.00)
13.	**Distance to Primary health Centre if not available in the village**	0.99*** (0.99–1.00)	0.99** (0.98–1.00)
	***Model IV-Socio-Demographic/Economic and Area Level Variables***		
14.	**Distance to closest town**	–	1.08*** (1.03–1.13)
15.	**Population of Village**	–	1.00 (0.99–1.00)
16.	**Household (HH) with Below Poverty Line (BPL) Card**		
	No BPL Card -reference		
	Yes-HH has BPL Card	–	1.10*** (1.03–1.19)
17.	**Household Wealth Quintile (Poor to Poorest)**		
	1. Marginally Poor (reference)		
	2. Moderately Poor	–	1.00 (0.89–1.12)
	3. Poor	–	0.84*** (0.75–0.94)
	4. Poorer	–	0.62*** (0.55–0.70)
	5. Poorest	–	0.51*** (0.45–0.58)
18.	**Eligible Woman Presently Working to earn in cash, kind, or both**		1.21*** (1.10–1.32)
19.	**Family Type**		
	Nuclear Family (reference)		
	Joint and Extended Family	–	1.20*** (1.11–1.30)
20.	**Religion**		
	Muslim (reference)		
	Hinduism and Others	–	1.15** (1.01–1.30)
21.	**Scheduled Caste**		
	General Caste (reference)	–	–
	Other Backward Caste	–	1.05 (0.93–1.17)
	Scheduled Caste & Tribe	–	0.97 (0.86–1.09)
22.	**Eligible Woman’s age in completed years**		1.00 (0.99–1.01)
23.	**Eligible Woman (EW) Age in completed years**	–	1.00 (0.99–1.01)
24.	**Eligible Woman (EW) Completed Education Level**		
	No schooling (reference)		
	Completed Primary School (year 9) and Above	–	0.93* (0.86–1.01)
25.	**EW’s Husband’s Completed Education Level**		
	No schooling (reference)		
	Completed Primary School (year 9) and Above	–	1.15*** (1.05–1.27)
	**Estimation of Model Fit**	**Model III**	**Model IV**
	Log likelihood	−10,143	− 9976
	Number of Observation	17,232	17,232
	AIC/BIC	20,329/20,492.	20,024 / 20,303

**Note:** Confidence intervals in parentheses; and significant p-value showing ***: *p* < 0.01, **: *p* < 0.05 and *: *p* < 0.10 levels. Log-likelihood and AIC/BIC values were also reported

#### Model I: IMFHL Program’s Main effects

Table 3 presents results from the first two multivariable logistic regression models I and II investigating the main effect of the level of household’s program exposure on the outcome adjusting for the survey round and including its moderation effect (an interaction intervention with round). The model I shows that SHG member households that received the added health intervention were 27% or 1.27 times more likely to have adequate knowledge of the key danger signs about obstetric complications as compared to women living in villages where no SHG program was conducted, that is, pure control villages (OR = 1.27, 95%CI: 1.15–1.41, *p* < 0.01).

Moreover, the woman from non-member households, but living in villages where other households received the SHG program or health intervention, had *lower* odds of the knowledge of the danger signs as compared to women in pure control villages (OR = 0.81, 95%CI: 0.74–0.89, *p* < 0.01). Lastly, model I showed that households in round II were 0.52 times or 48% less likely to know the danger signs as compared to households interviewed in round I (OR = 0.52, 95%CI: 0.49–0.56, *p* < 0.01).

#### Model II: interaction effects of IMFHL and survey round

The effect of program exposure on the outcome is expected to vary over round I and II. The program implementation is expected to have matured between round I in 2015 when the maternal health literacy component was started, and in 2017, 2 years into the intervention when round II was conducted. Therefore, in model II, an interaction term of program exposure with survey round was included to capture the change over time of the program effect on knowledge of danger signs.

Model II showed that the inclusion of the interaction term changed the direction and magnitude of the impact of the program exposure (of women in SHG plus health households) on the knowledge of danger signs compared to model I. The results showed that these women now reported 20% lower odds of knowing danger signs compared with non-member women in non- program or pure control villages (OR = 0.80, 95%CI: 0.68–0.95, *p* < 0.01). The shift in direction and magnitude observed for women in SHG plus health households between models I and II is suggestive of an underlying significant interaction effect of program exposure and survey round.

The results showed that women from households that received health intervention through SHGs had 2.14 times higher odds of knowing maternal danger signs in survey round II compared to the same household type in the round I when the health intervention was yet to start (OR = 2.14, 95%CI:1.73–2.65, *p* < 0.01). This means that women in the intervention group were roughly 1.712 (=0.80*2.14) times more likely to know maternal danger signs in survey round II compared to women in pure control group.

The results also showed that women in non-members households living in IMFHL program villages were 1.65 times *more likely* to have knowledge of danger signs in round II compared to the same household type in round I (OR = 1.65, 95%CI:1.73–2.65, *p* < 0.01). Since results showed that only women in SHG plus health household had higher and statistically significant odds of knowing danger signs in round II, the higher odds of knowledge found among non-members living in same villages suggests that the health literacy component contributed to the effect of diffusion of knowledge from members to non-members in program villages.

#### Models III and IV: effects of IMFHL program adjusted for confounders

The adjusted effect of program exposure is explored in model III, adding both individual health and health system confounders, whereas model IV included additional socio-demographic, economic and area-level variables, as presented in Table 4.

Overall, models III and IV show that the levels of household’s microfinance exposure have similar effects on the knowledge of maternal danger signs as observed previously in model II without confounders (Table 3), suggesting that the program effect is not in any way altered by the addition of confounders.

### Effects of individual health and health system variables on levels of maternal danger signs

The results for model III in Table 4 reveal that knowledge of danger signs was significantly and negatively associated with women’s increasing parity (OR = 0.96, 95%CI: 0.96–1.02, *p* < 0.01) and their experience of past pregnancy complications (OR = 0.84,95%CI: 0.78–0.90, *p* < 0.01). Furthermore, eligible women had lower odds of the knowledge of danger signs as contact with community front line workers increased during their last pregnancy (OR = 0.98, 95%CI: 0.98–1.00, *p* < 0.01) and as the distance of primary health facilities increased from their village (OR = 0.99, 95%CI: 0.99–1.00, *p* < 0.01).

Interestingly, institutional delivery was not significantly associated with knowledge of danger signs, except when women were discharged within 12 h after delivery as compared to women who delivered at home (OR = 1.22, 95%CI: 1.03–1.43, *p* < 0.01). However, even if statistically not significant, the results showed that higher duration of in facility stay post-delivery was generally associated with lower odds of knowledge of danger signs as compared to women who had home deliveries in both models (III and IV).

On the contrary, when primary exposure (SHG exposure) and round variables were removed from the analysis, the effect of institutional delivery on odds of the knowledge of danger signs was positive and significant (OR = 1.33, 95%CI:1.12–1.58, p < 0.01), suggesting that program and round variables take off the influence of institutional delivery on knowledge.

### Effects of Sociodemographic, economic and area-level variables on levels of maternal danger signs

The results show that keeping program exposure constant, the odds of knowledge of danger signs are statistically significant and *higher* in women belonging to households with below poverty limit (BPL) cards as compared to women from households without BPL cards.

To identify gradients of the poor even among the BPL card holders, this study included a constructed wealth quintile variable based on household amenities and assets. Results in model IV show that the odds of the knowledge of danger signs decrease as households become poorer with the poorest households in the fifth quintile, 49% or 0.51 times less likely to know all the danger signs as compared to women who were from marginally poor households (OR = 0.51, 95%CI: 0.45–0.58, *p* < 0.01). The effect of the working status, as seen among women presently working for cash or kind, revealed higher odds of the knowledge of danger signs as compared to non-working women (OR = 1.21, 95%CI: 1.10–1.32, *p* < 0.01). Similarly, women belonging to a joint or extended family have higher odd of the knowledge of danger signs compared to women in nuclear households (OR = 1.20, 95%CI: 1.11–1.30, *p* < 0.01). Interestingly, increasing levels of education of eligible women and their husbands have differing effects on knowledge of danger signs. While more educated women were *less likely* to have knowledge of danger signs compared to women with no schooling, the association was not strong enough to be statistically significant (OR = 0.93, 95%CI: 0.86–1.01, *p* < 0.10). However, women whose husbands had higher years of education were *more likely* to have the adequate knowledge of danger signs as compared to women whose husbands had no schooling (OR = 1.15, 95%CI: 1.05–1.27, *p* < 0.01).

## Discussion

Our study found that a microfinance program (SHG) is likely to improve the knowledge of maternal danger signs about obstetric complications among women when an additional health intervention is layered onto the SHG structure. It is also evident that the microfinance only program does not impact the knowledge outcome among rural women. The study also found evidence of diffusion of knowledge from members receiving health literacy to non-members living in the same program villages through social contacts and SHG peer network, as anticipated at the program design. Since results showed that women in SHG plus health household had higher and statistically significant odds of knowing danger signs in round II, the higher odds of knowledge found among non-members living in same villages suggests that the health literacy component contributed to the effect of diffusion of knowledge from members to non-members in program villages. The diffusion effects suggest that SHG members, when provided health literacy may be acting as *change agents* to promote health information among non-members, thereby extending program reach to more beneficiaries in program villages [43, 50]. According to diffusion studies elsewhere, we infer that the presence of SHG networks in villages influences knowledge diffusion by creating channels for inter-personnel communication in program villages and by the adoption of messages by SHG members themselves [39, 43, 48, 62]. SHG members may be acting as visible ‘model adopters ‘of new knowledge in the community leading to higher observability of newly gained maternal knowledge. Moreover, diffusion studies elsewhere showed that the spread of health messages from within community groups such as SHGs networks are more likely to facilitate social change and sustain the desired outcome when programmatic input ends [49, 50, 63].

The gain in knowledge is an interesting finding attributed to the health literacy program among members. However, as the knowledge of non-members was found to be low in villages where only SHGs were present (no added health), it reflects the limitations of the health system in improving knowledge of danger signs through routine channels comprising antenatal care and postnatal care.

While other studies in rural India have reached differing conclusions in the context of improving **routine** maternal care outcomes, no study has previously provided evidence on improvement in the levels of knowledge of key dangers signs in the context of health delivery through a microfinance program. Studies elsewhere have shown that membership in the SHG only program has a limited association with improving routine maternal and newborn health (MNH) practices. For example, three recent studies in rural UP showed that membership in SHG only programs did not improve the practice of three antenatal care check-ups, institutional delivery or post-natal care, but membership selectively improved practice of newborn cord care [51, 64, 65]. Similar inferences were also drawn from a larger 2019 study from UP that concluded that there were significant improvements (5–11 percentage points) in desired Maternal and Neonatal Health practices when a health behaviour change intervention was provided through the SHG platform compared to SHG alone [44]. Moreover, another study set in a different rural setting of India, Bihar, similarly observed that exposure to health intervention was notably associated with higher (at least four) ANC visits, and iron-folic acid consumption for 100 days [36].

Past studies show that early detection of maternal danger signs can help reduce maternal deaths in rural India that are otherwise precipitated due to delayed care-seeking by households [16, 24, 66]. Moreover, evidence from verbal autopsy studies in rural and tribal India found that while delays occur in all stages of care-seeking, a disproportionate number of women and households lack awareness of danger signs, the reason why serious complications are ignored and not treated in pregnancy, leading to deaths [16, 24, 66]. The lack of awareness in a rural setting, such as in this study, is exacerbated by high poverty and low education in general, and specifically for women who are further marginalised due to restrictive cultural norms that influence women’s independent decision-making and healthcare-seeking [24, 29, 66]. The potential of the integrated microfinance and health literacy (IMFHL) program for reducing these intersectoral disadvantages by providing health information and access to the most marginalised women is evident in this research. There is a need to guide the efficient implementation of such programs based on robust evidence and effectiveness. This research findings fit very well into that gap in the literature and generate evidence base for promoting successful implementation of such programs. Other studies have explored improved self-agency attained through membership in microfinance as a pathway to improving maternal health [38, 44, 51, 64, 65]. This study, however, hypothesised that improving knowledge of obstetric complications, which are important for detecting and early care-seeking, can be attained through health messages that are specific and culturally relevant as conducted in the IMFHL program.

A study conducted in another state of India, Bihar, with comparable poverty and maternal indicators as Uttar Pradesh, found a significant improvement in select indicators reflecting routine (non-emergency) maternal health utilisation among women enrolled in SHG program and receiving health literacy [36]. The study found that women who were SHG members and received health information about signs of pregnancy and delivery complications through SHG program showed a 20% improvement in their health behaviours [36]. However, in comparison to this study that used a pre-post-intervention and control design, the Bihar study used a post-intervention only study design and did not analyse the change in knowledge of maternal danger signs levels among women over time. Yet, the Bihar study found a marginal but, statistically non-significant improvement in seeking treatment for pregnancy complications. Our study adds significant value to the maternal health literature by providing empirical evidence of the program effect on the knowledge of maternal danger signs.

### Effect of Sociodemographic, economic and area level determinants of maternal danger signs

Above all, the IMFHL program’s main effect on knowledge of obstetric danger signs continued to remain statistically significant in this study even after controlling for the other confounders in the analysis. It is an important finding that delivers a significant message for policy design as such programs can reach rural marginalised women irrespective of their socio-economic background and health system construct.

### Individual health and health system variables

The health system variables examined in this study reflected health care practices across the continuum of maternity care contingent upon health services being available in an area. Irrespective of membership or program exposure status, the odds of maternal danger signs knowledge were lower for women with increasing parity, those who experienced pregnancy complications, and those living at increasing distances from primary health centres, keeping other factors constant. These findings are consistent with other studies from rural India and elsewhere that also reported lower utilisation of routine maternal health care services, especially, institutional delivery as parity and distance to health centre increase [67, 68]. Studies showed that, even in the case of villages where SHG only programs were present, membership was not associated with higher antenatal care, institutional delivery or post-natal care [51, 64, 65]. While increasing parity is also likely to be correlated with increasing age, the lower level of knowledge of danger signs found in our study with increasing parity could also be influenced by increasing age. Our study, however, has also controlled for age in the analysis, suggesting that lower knowledge in women is mainly driven by parity but not age.

The study also examined the impact of health system variables and found that women who received the minimum recommended four ANC with all key tests done in the last ANC visit do not exhibit any statistically significant improvement in their knowledge of obstetrics danger signs. As ANC is the mainstay of delivering health information to pregnant women under the formal health system, the not significant relation suggests that additional interventions for delivery of health knowledge such as through SHG would be beneficial in the rural UP context.

In addition to ANC and PNC, institutional delivery is a crucial health system intervention to prevent maternal complications, considering that the timing of most maternal complications is around the delivery and immediate post-delivery period. Our study found that institutional delivery is positively associated with higher knowledge of danger signs, but only when the eligible woman had been discharged from the health facility within 24 h post-delivery. Most women in our sample were found to be discharged from health facilities within 24 h post-delivery, despite Indian Government guidelines recommending a minimal of 48 h stay post normal delivery [12]. Studies found that the reduced in-hospital stay poses a risk for recently delivered women who can still experience complications in the immediate post-partum period, especially post-partum haemorrhage [16, 24]. Insights from verbal autopsy studies from rural Uttar Pradesh [24], and elsewhere in India [16, 67] using maternal deaths as case studies found instances of women suffering from a severe postpartum haemorrhage on the same day they were discharged from the facility.

### Effect of Sociodemographic, economic, and area-level variables on levels of maternal danger sign

Our study found that women in poor households with below poverty cards were more likely to know danger signs compared to those without BPL cards. Although the possession of BPL cards by households is indicative of poverty, the use of BPL cards as an effective criterion to capture the poor is widely debated [69, 70]. However, as revealed by an asset-based wealth quintile used in this study, there is a decreasing association of knowledge of danger signs as households become poorer with the poorest least likely to know the danger signs.

Previously studies in India have shown a strong inverse relationship between wealth and experiencing maternal complications with the poorest most likely to experience complications [71]. Similar associations in the context of knowledge are seen in our study with poorest households lacking knowledge of danger signs; however, the study also suggests that exposure to microfinance plus health intervention does not improve knowledge of danger signs across wealth gradients. One plausible explanation could have been that the IMFHL program is not able to retain poorest households in the program. An earlier study by Hazra et al. (2020), using the same program’s data as this study, found that routine indicators of maternal health (three tests during ANC visits, consumption of 100 IFA tablets in pregnancy) were substantially higher among the most marginalised SHG members than least marginalised SHG members [44]. Although there are differences in the construction of marginalisation index between the Hazra et al. (2020) and the wealth quintile in our study, the evidence generated by both studies shows that delivering health literacy through microfinance programs can improve maternal health outcomes.

The current study showed that social factors such as belonging to a joint or extended family, being part of the majority Hindu religion, and working women are more likely to improve the knowledge of all maternal danger signs. Our study extends findings of previous studies that showed a positive association between working women and the institutional delivery [64] and contrasts a recent study failing to show any association [44].

One aspect that our study reveals is the significance of the husband’s education level on the eligible woman knowledge of crucial danger signs. While this association was not observed for the woman’s level of education in our study, other studies from rural India have previously shown a similar inconsistent relationship between increasing levels of women’s education and experiencing complications. Moreover, while previously, the literature has established stronger associations for increasing education and institutional delivery, our study adds value by highlighting the limited impact of women’s education on danger signs levels. Our findings also differ from a study conducted in Uganda that showed a higher association of maternal and newborn danger signs awareness and maternal education level [72]. However, this contradiction in the influence of woman’s and her husband’s education on knowledge of maternal danger signs may emphasise that to achieve improved maternal health care; programs need to collectively include both fathers and mothers in the process. It is more relevant in the context of rural UP where a previous study suggested that husbands were not always involved in the discussions of Post-Partum Haemorrhage (PPH) and were unaware of their wife’s experience of it [24, 67]. Women reported feeling shame in discussing PPH with their husbands which restricted the husband’s involvement in care-seeking decision for PPH. Our results are supported by a previous study from UP that reported an influential role of husbands schooling with a higher likelihood of pregnancy registration, receiving ANC and institutional delivery [73]. The higher association of the husband’s education observed in our study is likely to improve care-seeking for women during maternal complications and reinforce knowledge of maternal danger signs. However, further research is required to examine the interrelationship between women and their husbands’ level of knowledge, especially for maternal complications.

### Study strengths and limitations

A strength of this study was that the health intervention was mainly delivered through the existing structure of the SHG platform, suggesting that integrated health interventions can feasibly be scaled up. These results apply to the Indian context, where the SHG platform is being actively promoted by the Indian Government as a poverty eradication tool and presents an opportunity to integrate maternal health service delivery to marginalised populations.

Another strength lies in the study design itself; it is a community-based cross-sectional study capturing data from two-time points pre-post intervention covering a substantial sample, that is 17,232 women. The study design allows for evaluation using a pre-post quasi-randomised survey using a relatively large sample size. Such studies are rare in a highly populated and vast country like India. Moreover, the study design also generates new evidence on the diffusion effect of knowledge on maternal health from SHG program members receiving health literacy to non-members living in the same villages. Therefore, the presence of a diffusion effect can guide program implementation seeking to maximise the coverage of program beneficiaries. Evidence generation and programs designed to capture such information would be hugely beneficial for India in case of maternal health intervention that would be potentially able to break through multiple layers of disadvantage and be cost-efficient. While previous studies have captured recall of *any* danger sign as an indication of knowledge of danger signs, our study comprehensively examined the impact of *all* danger signs that are commonly associated with preventable causes of maternal mortality across multiple phases of pregnancy and delivery. Our study also provides a theoretical framework to situate determinants of maternal danger signs by integrating the widely acclaimed three delays models [74] and social determinants models established by Mosely et al., and Marmot et al. [75, 76]. Another strength of this study lies in the fact that it included a range of other potential social determinants of health behaviours that are reflective of individual, households, community and area-level influence on the outcome as compared to other studies that are limited in scope. However, while the study provides insights into the knowledge of danger signs levels among women, the findings should be interpreted considering study limitations that are discussed below.

Firstly, programmatic requirements guided the selection of intervention and comparison areas for implementation, preventing randomisation of blocks. However, by selecting roughly matched control and intervention blocks based on the proportion of Scheduled Caste and Scheduled Tribe from the same districts, potential bias in the allocation of blocks is reduced. Moreover, as the selection of villages was based on a different parameter to block selection, that is based on SHG population coverage, the effect of clustering of gram panchayats (main villages) within blocks is minimised. Moreover, although the same villages were visited in both survey rounds, different households were sampled in both rounds. Additionally, it is possible that a few households may have been captured in both survey rounds; however, these would be a very limited sample. Another limitation is that the data and outcome variable were based on self-recall, which is susceptible to recall bias as well as social desirability. However, the potential for these errors was minimised by the inclusion of women who had delivered within 12 months post-delivery and the recruitment of trained and experienced interviewers to collect the data.

Another limitation is examining area-level effects across villages while there may potentially be clustering within villages. While the survey was designed to sample households within villages or GPs, the GPs were selected from comparison blocks roughly matched to intervention blocks suggesting that villages within blocks are also expected to be broadly similar. Consequently, the standard cluster sampling effects did not apply here, and there seemed to be rather similarities across villages than within villages. However, in India, rural health services and facilities are implemented at the level of the village, and they are likely to vary across villages. Therefore, it makes more sense to study the effect of area-level health system variables such as contact with community health workers and distance to Primary health centre across villages and not within villages on the outcome.

Furthermore, the way the survey was designed, sampled women comprising SHG and Non-member, are selected from the same villages. However, in some cases, the women sampled in a village were either all members or all non-members, allowing only little variation of the program effect within the village. Therefore, it makes more sense to assess the determinants of outcome variables across villages instead of within villages as the clustering within villages may be less likely to have an influence here. Moreover, the study considers membership across villages. Lastly, the study’s cross-sectional design prevents concluding causality from observed relationships. The investigation of poor and rural women in districts of UP with greater marginalisation as represented by higher scheduled caste and tribal population prevent generalisation to UP or to other states of India that are culturally different.

### Policy and program implications

Based on the study findings, policies may aim to strengthen the delivery of maternal health literacy information to women through an SHG program with an integrated health program in order to accelerate knowledge on maternal danger signs and consequently reduce maternal mortality towards the Sustainable Development Goals.

The study shows that the presence of an SHG alone would not be adequate to shift key maternal indicators related to knowledge of preventable obstetric complications and potentially improve care-seeking. Moreover, in the prevailing rural Indian context with low ANC and PNC coverage, the SHG network can act to strengthen awareness of danger signs in the community and support referral services.

Given the Inidan Government’s emphasis and active work under the National Rural Livelihood Mission and State Rural Livelihood Missions, our study demonstrates that such SHG platforms provides an opportunity to disseminate information about desired health practices and challenges the traditional social norms that restrict women’s mobility and care-seeking, especially in the post-partum period. The peer effect of the SHG can potentially shape behaviours for younger mothers and encourage higher health facility use as parity increases among women. The presence of SHG in a village provides the opportunity to improve linkages with frontline workers to improve the quality of the identification of higher maternal risk women and early follow up. Studies have shown that health interventions added to SHG networks improved coordination between SHGs and frontline workers [39, 40]. Lastly, the evidence supporting the diffusion effect of maternal knowledge from members to non-members illustrates the presence of the SHG social networks and presents a valuable opportunity for wider dissemination of health information. Further, qualitative research is required to explore in-depth the pathways of diffusion with a view to improving knowledge levels among members and non-members alike.

## Conclusion

The IMFHL program analysed in this paper sought to provide health literacy and financial access to marginalised households in one of India’s most populated and developmentally disadvantaged states, Uttar Pradesh. This study finds that maternal health interventions delivered through microfinance programs can impact women’s knowledge of danger signs leading to improved care-seeking. The addition of a maternal health literacy component along with access to funds for health reasons through the SHG is likely to complement the underlying microfinance platform as members gain dual value from membership. Both components synergistically operating may also help to interrupt the mutually reinforcing cycle of poverty and poor maternal health. The SHG model used in the IMFHL program additionally allows for rapidly scaling up, and the dissemination of developmental services to a largely marginalised and hard to reach population. Furthermore, an earlier study found microfinance and health interventions to be cost-effective in promoting maternal and neonatal health in Bihar, India which has similar health and developmental indicators to Uttar Pradesh [77]. Despite gains in knowledge of maternal danger signs, further research is required to establish the impact of membership in IMFHL programs on actual care-seeking practices among women who have experienced complications in the maternity period. The presence of diffusion of knowledge from SHG members to neighbouring non-members presents the opportunity to reach a larger population in program areas in resource-poor settings and provides an example of a community-based model that could be potentially scaled up to promote selected maternal health interventions.

This research also provides theoretical frameworks for examining the determinants of maternal complication combining previously established frameworks related to delays and determinants of care-seeking. Moreover, this research generates evidence on the role of a community organisation to strengthen health literacy in setting with limited supply-side health services. Lastly, by demonstrating the differential impact of SHG alone and SHG plus health on maternal health outcome, this research draws differences in the way traditional woman’s group are viewed to contribute to maternal health and makes a distinction in favour of SHG as a feasible platform capable of delivering health interventions.

## Supplementary Information

**Additional file 1.** Shows the survey questionnaire utilised for data collection from eligible woman.

## Data Availability

The datasets used and/or analysed during the current study are available from the corresponding author on reasonable request.

## Abbreviations

SHG: Self- help groups
IMFHL: Integrated microfinance and health literacy
MDG: Millennial Development Goals
SDG: Sustainable Development Goals
MMR: Maternal mortality ratio
EMOC: Emergency Medical Obstetric Care
UP: Uttar Pradesh
USD: United states dollars
ANC: Antenatal care
IFA: Iron-folic acid
PPH: Postpartum haemorrhage
PNC: Post-natal care
SC/ST: Scheduled caste /Scheduled tribe
GP: Gram panchayats
PP: Percentage point
CAPI: Computer-assisted personal interview
PCA: Principal Component Analysis
OR: Odds ratios
CAIC: Consistent Akaike Information Criteria
BIC: Bayesian Information Criteria
PHC: Primary Health Centre
KM: Kilometre
EW: Eligible woman
ASHA: Accredited Social Health Activist
ANM: Auxiliary Nurse Midwife
AWW: Anganwadi worker
CI: Confidence intervals
BPL: Below poverty limit
MNH: Maternal and newborn health
