# Supplementary material for: The knowledge of danger signs of obstetric complications among women in rural India: evaluating an integrated microfinance and health literacy program
Source: BMC Pregnancy Childbirth. 2021 Jan 23;21:79. doi: 10.1186/s12884-021-03563-5 (PMC7824939; doi:10.1186/s12884-021-03563-5)
Supplement: Supplementary file 1 — Additional file 1. Shows the survey questionnaire utilised for data collection from eligible woman. [file 12884_2021_3563_MOESM1_ESM.pdf]

CONFIDENTIAL
(For research purpose only)

HEALTH BEHAVIORS AND COVERAGE OF HEALTH SERVICES IN UTTAR PRADESH

WOMAN QUESTIONNAIRE महिला प्रश्नावली
(SCALE UP - BASELINE)

**A. Identification**	
District code जिले का कोड	☐☐
Block code ब्लाक कोड	☐☐☐
GP code जी पी कोड	☐☐☐☐☐
Village/Purva name & code गाँव/पुरवा का नाम और कोड ____________________	☐☐
Study arm सर्वेक्षण के वाहें — Intervention (**SHG + Health** intervention) = 1; Comparison 1 (**SHG** , **No** intervention) = 2; Comparison 2 (**No SHG** , **No** intervention) = 3	☐
Type of household परिवार के प्रकार — **SHG** HH………………………………………….= 1; Non-SHG HH from SHG cluster (**NSHG1**)….......= 2; Non-SHG HH from Non-SHG cluster (**NSHG2**) = 3	☐
Household number घर का नंबर	☐☐☐
Name of SHG if woman is from SHG household<br>यदि महिला समूह परिवार से है तो समूह का नाम	____________
SHG Code समूह का कोड	☐☐☐☐☐☐
**B. Total number of visit(s)** कुल विजिट	☐
**C. Result status of the Women Questionnaire**	☐

Completed पूरी की गयी	1
Partly completed आंशिक रूप से पूरी की गयी	2
Not at home घर पर नही	3
Refused साक्षात्कार से मना किया	4
Other (specify) अन्य (स्पष्ट करें)________________	7

CODE ☐☐<br>(Code of the Investigator प्रश्नकर्ता का कोड)	DATE DD ☐☐ MM ☐☐ YYYY ☐☐☐☐<br>Start Time: ☐☐ ☐☐

POPULATION COUNCIL
Ideas. Evidence. Impact.

Zone 5A, GroundFloor, India Habitat Centre
Lodi Road, New Delhi – 110 003
Tel: 011-24642901/02; Fax: 011-2464290

Version dated: 21 Nov. 2014

Informed Consent

Namaste. My name is ----------------------------. On behalf of the Population Council, New Delhi, we are conducting a survey with the aim of improving the health of women and their children. In this context we are trying to assess thepractice of maternal and newborn health behaviors in your village. I would like to discuss some topics with you in this regard. This will take about 45 minutes of your time. I will be discussing various topics like health care, care of newborn babies, nutrition etc. We would very much appreciate your participation, as this discussion with you will be very useful to take steps for raising awareness and providing better services in your village.

I assure you that you are at no risk by participating in this survey. The information you provide will mainly be health related and will be used for reaserch purpose only. It will also be made sure that your identity will not be revealed in any way.

Still if you do not want to answer a particular question or all questions, you can refuse. Also, you can leave the interview in between if you so wish to. However, we hope to receive your full participation in this survey and your participation is very critical.

Do you want to ask me anything about the study?

ANSWER RESPONDENT'S QUESTIONS

May I begin now?

नमस्ते मेरा नाम ....है। पपुलेशन काउन्सिल दिल्ली के तरफ से हम महिलाओं और उनके बच्चो के स्वास्थ्य में सुधार के उद्धेश्य से एक प्रोजेक्ट पर काम कर रहे हैं। इस संदर्भ में हम आपके गॉव के लोगों द्धारा स्वास्थ्य संबंधी विभिन्न व्यवहारों के बारे में पता लगाने की कोशिश कर रहे हैं। मैं इस संबंध में कुछ विषयों पर आपसे बातचीत करना चाहती हूँ। इसमें आपका लगभग 45 मिनट का समय लगेगा। मैं विभिन्न विषयों जैसे स्वास्थ्य की देखभाल, नवजात शिशुओं की देखभाल, पोषण आदि के बारे में चर्चा करूंगी। हम आपकी भागदारी के बहुत आभारी रहेंगे क्योंकि आपके साथ की यह चर्चा जागरूकता बढ़ाने, तथा आपके गॉव में बेहतर स्वास्थ्य सेवायें प्रदान करने हेतु कदम उठाने सहायता करेगी।

मैं आपको विश्वास दिलाती हूँ कि इस सर्वेक्षण में भाग लेने में आपको कोई जोखिम नहीं है। आपके द्धारा प्रदान की जाने वाली जानकारी मुख्य रूप से केवल स्वास्थ्य से संबंधित होगी। मैं आपको यह भी विश्वास दिलाती हूँ कि आपकी पहचान किसी भी रूप में प्रकट नहीं की जायेगी।

फिर भी यदि आप किसी खास प्रश्न का उत्तर नहीं देना चाहती हैं तो आप मना कर सकती हैं। इसके साथ ही यदि आपकी इच्छा हो तो आप इस चर्चा को बीच में ही छोड़ सकती हैं। परन्तु हम इस सर्वेक्षण में आपकी पूरी भागीदारी की आशा करते हैं तथा इसमें आपकी भागीदारी बहुत जरूरी है।

क्या आप इस अध्ययन के बारे में कुछ पूछना चाहती हैं?

उत्तरदाता के प्रश्नों के जवाब दें।

क्या मैं अब शुरू कर सकती हूँ?

Respondent agrees to be interviewed ….1
उत्तरदाता साक्षात्कार देने के लिए सहमत है
↓
START Interview
इंटरव्यू शुरू करें

Respondent does not agree to be interviewed.....2
उत्तरदाता साक्षात्कार देने के लिए सहमत नहीं है
↓
END Interview
इंटरव्यू समाप्त करें

A. BACKGROUND INFORMATION

S.N.	Questions	Coding categories	Codes	Skip to
A1	What is your age?<br>**INS:Record age in completed years**<br>आपकी उम्र कितनी है?<br>निर्देश: पूरे किये गये वर्षो में उम्र रिकार्ड करें		☐☐	
A2	Had you ever attended school?<br><br>क्या आपने कभी स्कूल में पढ़ाई की है?	Yes हाँ<br>No नहीं	1<br>2	 → **A4**
A3	What is the highest standard that you have completed?<br>**INS: Code exact number of years of schooling.**<br><br>आपने कहां तक पढ़ाई की है?<br><br>निर्देश: स्कूल की पढ़ाई के वास्तविक वर्ष कोड़ करें	Standard कक्षा<br><br>Technical education after 10th Class<br>दसवी कक्षा के बाद तकनीकी शिक्षा (डिप्लोमा)<br>BA/BSC बीए/बीएससी<br>Post graduate पोस्ट ग्रेजुएट<br>Other (specify)____________<br>अन्य (स्पष्ट करें)	☐☐<br><br><br>13<br>15<br>17<br><br>77	 <br><br>→ **A5** (13, 15, 17, 77)
A4	**INS: Check A3, if standard is up to 04, ask:**<br>Can you read and write?<br>निर्देश : **A3** चेक करें, यदि कक्षा 04 तक हो तो पूछें।<br>क्या आप पढ़/लिख सकती हैं?	No नहीं<br>Yes, can read<br>हाँ, पढ़ सकती है<br>Yes, can read and write<br>हाँ, पढ़ और लिख सकती है	1<br><br>2<br><br>3	
A5	a) Can your husband read and write?<br><br>क्या आपके पति पढ़/लिख सकते हैं?	No नहीं<br>Yes, can read<br>हाँ, पढ़ सकते है<br>Yes, can read and write<br>हाँ, पढ़ और लिख सकते है	1<br><br>2<br><br>3	→ **A6**
	b) If yes, what is the highest standard of schooling your husband has completed?<br><br>उन्होनें कहां तक पढ़ाई की है?	Standard कक्षा<br><br>Technical education after 10th Class<br>दसवी कक्षा के बाद तकनीकी शिक्षा (डिप्लोमा)<br>BA/BSC बीए/बीएससी<br>Post graduate पोस्ट ग्रेजुएट<br>Other (specify)____________<br>अन्य (स्पष्ट करें)<br>Never gone to school<br>स्कूल में कभी पढ़ाई नहीं की	☐☐<br><br><br>13<br>15<br>17<br><br>77<br><br>55	
A6	Some men drink alcohol or take drugs. Does your husband drink, often, sometimes, or never?<br>कई आदमी अक्सर शराब पीते है। क्या आपके पति शराब पीते है? अगर हाँ तो क्या अक्सर, कभी कभी या बिलकुल नही पीते है?	Often<br>आम तौर से<br>Sometimes<br>कभी कभी<br>Never<br>कभी नही	 1<br><br>2<br><br>3	
A7	Are you presently doing any work to earn either in cash/kind or both?<br>क्या आप आजकल पैसा कमाने के लिए कोई ऐसा काम करती है जिसमें आपको नकद/वस्तु के रूप में या दोनों तरह से भुगतान किया जाता है?	Yes हाँ<br>No नहीं	1<br>2	 → **A9**

S.N.	Questions	Coding categories	Codes	Skip to
A8	What is the **main** work you do?<br><br>आप मूख्य रूप से क्या काम करती हैं ?	Petty business/Kirana shop<br>छोटा धंधा/किराना की दुकान<br>Own farming<br>अपनी खेती<br>Dairy<br>दूध बेचना<br>Poultry/Fishery/Piggery<br>मुर्गीपालन/मछली पालन/सूअर पालना<br>Goat, Cow & Buffalo rearing<br>बकरी, गाय या भैंस पालना<br>Stitching<br>सिंलाई<br>Agricultural labouror<br>खेती मजदूर<br>Non-agricultural labouror<br>गैर खेती मजदूर<br>Govt./Private service<br>सरकारी/प्राईवेट नौकरी<br>Handy craft<br>हस्तशिल्प<br>Other (specify)_______अन्य (स्पष्ट करें)	01<br>02<br>03<br>04<br>05<br>06<br>07<br>08<br>09<br>10<br>77	
A9	Has your husband ever prohibited you from getting a job, going to work, trading, earning money or participating in income generating projects?<br>क्या आप के पति ने आपको नौकरी करने से/काम पर जाने से/व्यापार करने से/काम कर के पैसे कमाने से कभी रोका है?	Yes हाँ<br>No नहीं	1<br>2 →	 **A11**
A10	Did he try to stop you from working for earning in the last 12 months?<br>क्या आप के पति ने आपको पिछले 12 महिनों में काम कर के पैसे कमाने के लिये कभी रोका है?	Yes हाँ<br>No नहीं	1<br>2	
A11	**INS: Check A7. If code is 1, then ask A11 to A13; otherwise skip to A14.**<br>On an average how much money do you earn in a month, either in cash or kind?<br>निर्देशः अगर **A7** में कोड़ 1 है (नकद/वस्तु के लिये काम करती है) तो **A11** से **A13** पुछें नहीं तो **A14** पर जाएं।<br>आमतौर से एक महीने में नकद/वस्तु के रूप में आपकी कितनी आमदनी हो जाती है?	Rs.	☐☐☐☐☐	
A12	Who keeps your earning?<br>**INS: Read the options and ask.**<br><br>आप जो पैसे कमाती है वह किसके पास रहता है ?<br><br>निर्देशः उत्तरों को पढकर पूछें।	Keeps total amount with her<br>सारे कमाइ अपने पास रखती हैं<br>Gives total earning to husband<br>सारे कमाइ पति को दे देतीं है<br>Gives total earning to mother-in-law<br>सारे कमाइ सास को दे देतीं है<br>Keeps some amount with her and rest give to other<br>कुछ अपने पास रखते है और बाकी परिवार में अन्य किसी को दे देतीं हैं<br>Earn in kind<br>वस्तु के रूप में भुगतान किया जाता है	1<br>2<br>3<br>4<br>5	

S.N.	Questions	Coding categories	Codes	Skip to
A13	Can you spend your earned money in the way you want?<br>**INS: Read the options and ask.**<br><br>आप जो पैसे कमाती है, उसको क्या अपने हिसाब से खर्च कर सकती है?<br><br>निर्देश: उत्तरों को पढकर पूछें।	Always हमेशा<br>Occasionally कभी कभी<br>Never and not possible कभी नहीं एवं मुमकिन नही	1<br>2<br>3	
A14	Has your husband ever refused to give you money you needed for household expenses even when he has money for other things?<br><br>क्या आपके पति ने कभी आपको घर के जरूरत के खर्चों का पैसे देने से मना किया है, जबकि उन के पास पैसे थे?	Yes हाँ<br>No नहीं	1<br>2	
A15	How many living children do you have?<br>**[If none, code "0" in all three boxes]**<br>How many girls & boys?<br><br>आपके कुल कितने बच्चे जीवित हैं? कितने लड़के, कितने लड़कियां ?<br>निर्देश: यदि नही तो तीनो बक्सो में 0 रिकार्ड करें	Total कुल<br>Male लड़के<br>Female लड़कियां	☐<br>☐<br>☐	
A16	Have you ever given birth to a boy or a girl who was born alive but did not survive? If yes, how many?<br>**[If none, code "0" in all three boxes]**<br><br>क्या आपने कभी ऐसे लड़के या लडकी को जन्म दिया हे जो जीवित पैदा हुआ था लेकिन अब वह जीवित नहीं हैं? कितने लड़के, कितने लड़कियां ?<br>निर्देश: यदि नही तो तीनों बक्सो में 0 रिकार्ड करें	Total कुल<br>Male लड़के<br>Female लड़कियां	☐<br>☐<br>☐	
A17	**If any death reported in A16, then ask:**<br>Did any of them die before completing 1 month? If yes how many?<br>**[If none, code "0" in all three boxes]**<br>अगर कोई बच्चा जीवित पैदा हुआ था लेकिन अब वह जीवित नहीं हैं तो पूछें।<br>क्या उनमें से कोई बच्चा एक महीना पूरा होने से पहले ही मर गया था? यदि हाँ, तो कुल कितने बच्चें एक माह के पहले मर गये? कितने लड़के, कितने लड़कियां ?<br>निर्देश: यदि नही तो तीनो बक्सो में 0 रिकार्ड करें	Total कुल<br>Male लड़के<br>Female लड़कियां	☐<br>☐<br>☐	
A18	**INS: Add A15 & A16 and ask:**<br>So, in total you gave _______live births in your lifetime.<br>**INS: If response is NO, go back to A16 and A17 to find out the discrepancy and correct the responses.**<br><br>निर्देश: **A**15 और **A**16 का योग करें और पूछें।<br>तो आपने अपने जीवन में _____________ जीवित बच्चों को जन्म दिया है?<br>निर्देश: यदि जवाब नहीं में दे तो **A**15 और **A**16 वापस जाकर अंतर का कारण देखें तथा जवाब को सही करें	Total कुल<br>Male लड़के<br>Female लड़कियां<br><br>Yes हाँ<br>No नही	☐☐<br>☐<br>☐<br><br>1<br>2	

S.N.	Questions	Coding categories	Codes	Skip to
A19	Some pregnancies end up in delivery of dead child or spontaneous abortion or women herself induces abortion. Kindly tell me how many of your pregnancies ended up as:<br>**INS: Read each option one by one**<br>कुछ बच्चें मृत पैदा होते है, कुछ गर्भ खुद से ही गिर जाते है और कुछ गर्भ महिला अनेक कारणों से खुद से गर्भपात करवा देती है। कृपया बताये किं कितने गर्भ का ऐसा कुछ हुआ?<br>निर्देश: हर कोड़ को एक एक कर के पुछें।	Still birth मृत बच्चे के रूप में पैदा हुआ<br>Sponteneous abortion गर्भ खुद से गिर गया<br>Induced abortion आप ने गर्भपात करवाया<br>Total pregnency lost कुल कितनी बार गर्भ का नुकसान हुआ	☐<br>☐<br>☐<br>☐	

B. ANTE NATAL CARE

S.N.	Questions	Coding categories	Codes	Skip to
B1	How many TT injections did you receive during your last pregnancy?<br>पिछली गर्भावस्था के दौरान आपको कितने टीटी इंजेक्शन लगे थे?		☐	
B2	During your pregnancy how many<br>(a) IFA tablets did you receive<br>(b) IFA tablets did you consume<br>पिछली गर्भावस्था के दौरान<br>(a) आप को कितनी आयरन की गोलियां मिलीं?<br>(b) आप ने कितनी आयरन की गोलियां खाई?	No. of IFA tablets received कुल आयरन की गोलियां मिलीं<br>**CODE 999 IF NOT SURE**<br>No. of IFA tablets consumed कुल आयरन की गोलियां खाई<br>**CODE 999 IF NOT SURE**	☐☐☐<br>☐☐☐	
B3	During pregnancy how many<br>(a) TT injections must be administered<br>(b) IFA tablets must be consumed<br>गर्भावस्था के दौरान कितने<br>(a)टीटी इंजेक्शन लेगवाने चाहिए ?<br>(b) आयरन की गोलियां खानी चाहिए?	(a) Number of TT injections टीटी इंजेक्शन की संख्या<br>Do not know नहीं जानती<br>(b) Number of IFA tablets आयरन की गोली की संख्या<br>Do not know नहीं जानती	☐<br>8<br>☐☐☐<br>888	
B4	How many times did you receive ANC checkups during your last pregnancy?<br>पिछली गर्भावस्था के दौरान आपने कितनी बार प्रसव पूर्व जाँचें करवाइ थी?	Number of ANC checkups कुल प्रसव पूर्व जाँचों की संख्या<br>Not received any ANC checkup कोइ जाँच नही करवाइ थी	☐<br>0	→ **B6**
B5	As part of the antenatal checkups during your last pregnancy, were any of the following done at least once?<br>**INS: Read each option.**<br>आपकी पिछली गर्भावस्था के दौरान कौन कौन सी प्रसव पूर्व जाँचों को कम से कम एक बार करवाया था?<br>निर्देश: एक एक करके पुछें	a) Urine test पेशाब की जाँच<br>b) Blood test खून की जाँच<br>c) Blood Pressure रक्तचाप या ब्लड प्रेशर<br>d) Weight वजन<br>e) Abdominal examination पेट की जाँच<br>f) Ultrasound अल्ट्रासाउंड	**Yes** हाँ / **No** नही / **DK** पता नहीं<br>1 2 8<br>1 2 8<br>1 2 8<br>1 2 8<br>1 2 8<br>1 2 8	
B6	What is the minimum number of antenatal checkups that a pregnant woman should receive?<br>किसी गर्भवती महिला को कम से कम कितनी बार प्रसव पूर्व जाँचें करवानी चाहिए?	Number of antenatal checkups कुल प्रसव पूर्व जाँचों की संख्या<br>As many as required जरूरत के अनुसार<br>Do not know नहीं जानती	☐<br>7<br>8	

S.N.	Questions	Coding categories	Codes	Skip to
B7	Did you take deworming tablets anytime during your last pregnancy?<br>क्या आपने पिछली गर्भावस्था के दौरान पेट के कीडे को मारने की गोलियां खायी थी?	Yes हाँ<br>No नही	1<br>2	

	(a) Did any of the following health workers or any member from SHG contact you or did you meet them during your last pregnancy?<br>पिछली गर्भावस्था के दौरान क्या ....ने आपसे या आपने ....से सम्पर्क किया था?	Yes हाँ	No नही	(b) If yes, taking the two together how many such contacts was made?<br>दोनो मिलाकर कुल एसे कितनें बार सम्पर्क हुऐ?
B8				
A	ANM एएनएम	1	2	☐
B	ASHA आशा	1	2	☐
C	AWW आंगनवाड़ी कार्यकर्ता	1	2	☐
D	VO/SHG Member/SS ग्राम संगठन/समूह के सदस्य/स्वास्थ्य सखी	1	2	☐

B9	During your last pregnancy, did anyone advice you on the care you should take during pregnancy, PNC and newborn care? If yes, what all advice you had received?<br>**INS: Circle all the spontaneous answer you recived. Probe one by one all for care that were not mentioned**<br>पिछली गर्भावस्था के दौरान क्या आपको गर्भावस्था, प्रसव के बाद, या नवजात शिशु के बारे में कोई सलाह मिली थी? अगर हाँ तो क्या क्या सलाह मिली थी?<br>निर्देश : माँ जो जो बताती है वह जवाब पहले रिकार्ड करें। फिर एक एक कर के बाकी के सवाल पूछें।	**Circle all the Spontanious responses**<br>खुद से बताया	**After Probing**<br>पूछने पर बताया<br>**YES**	**NO**	B10.<br>If yes, who advised you?<br>अगर हाँ, तो आपको सलाह किस ने दी?
A	Must undergo at least 4 ANC checkups<br>कम से कम 4 बार प्रसव पूर्व जॉच जरूर करनी चाहिए	1	1	2	☐
B	Need and importance of taking IFA tablets<br>गर्भावस्था के दौरान आयरन की गोलियों का महत्व	2	1	2	☐
C	Need and importance of taking deworming tablets<br>गर्भावस्था के दौरान पेट के कीडे मारने की गोलियों का महत्व	3	1	2	☐
D	Symptoms of pregnancy complications<br>गर्भावस्था की जटिलताओं या खतरो के लक्षण	4	1	2	☐
E	Must undergo PNC<br>प्रसव के बाद चैकअप की जरूरत	5	1	2	☐
F	Immediate breastfeeding after birth<br>जन्म के तुरंत बाद स्तनपान की जरूरत	6	1	2	☐
G	Delay first bathing by at least 2 days<br>नवजात शिशु को कम से कम 2 दिन के बाद नहलाना	7	1	2	☐
H	Keep the baby warm by KMC/skin ro skin care<br>बच्चे को गरम रखने के लिए कगारू विधि/त्वचा से त्वचा की रक्षा का इस्तेमाल	8	1	2	☐
I	Exclusive breastfeeding to baby for 6 months and not to give anything else<br>6 महीने तक बच्चे को केवल स्तनपान कराना, और कुछ न खिलाना/पिलाना	9	1	2	☐

Codes for B10
ASHA आशा =1
AWW आंगनवाड़ी कार्यकर्त्री=2
ANM एएनएम =3
Doctor डाक्टर = 4
VO/SHG Member/SS ग्राम संगठन/समूह के सदस्य/स्वास्थ्य सखी =5
Dai/Elders in family/ friends दाई/घर के बड़े बुजुर्ग/दोस्त =6

C. DELIVERY PREPAREDNESS

S.N.	Questions	Coding categories	Codes	Skip to
C1	What advance preparation did you/your family members make to manage in case of any pregnancy/delivery complications?<br>**INS: Multiple responses possible. Code all given responses.**<br><br>**Probe: Any other arrangement?**<br><br>आपने या आपके परिवार वालों नें गर्भावस्था या प्रसव के दौरान होने वाली जटिलताओं से निपटने के लिये क्या क्या तैयारियां की थी?<br><br>निर्देशः कई जवाब संभव हैं, दिए गए सभी जवाब रिकार्ड करें।<br><br>फिर पूछें: और क्या तैयारीयां की थी?	Decided on place of delivery-home or health facility<br>घर पर या स्वास्थ्य केन्द्र पर प्रसव करेंगें उस का निर्णय	01	
		Knew the facility that could provide emergency care<br>स्वास्थ्य केन्द्र की जानकारी जो आपातकालिन सुविधा प्रदान करता है	02	
		Saved/arranged money for delivery expense or in case of emergency<br>प्रसव के खर्चों या जटिलताओं की हालत मैं खर्च करने के लिए पैसे जमा करके या इंतजाम करके रखना	03	
		Advance arrangement of transportation to go to facility<br>स्वास्थ्य केन्द्र तक जाने के लिए यातायात के साधन का पहले से इंतेजाम	04	
		Identified institution where to rush in case of emergency<br>महिला को किसी जटिलता के हालात में किस स्वास्थ केंन्द्र पर जाना चहिये उसकी पहचान	05	
		Identified people to accompany the woman<br>महिला के साथ अस्पताल जाने वाले सदस्य की व्यवस्था	06	
		Identified people to take care of children at home<br>घर पर बच्चों की देखभाल करने वाले सदस्य की व्यवस्था	07	
		Others (specify) अन्य (स्पष्ट करें)__________________	77	
		Not done any preparation<br>कोइ भी तैयारी नहीं की	55 →	**D1**
C2	Who adviced you to do these preparations?<br>**INS: Multiple responses possible. Code all given responses.**<br><br>इन तैयारियों को पहले से करने की सलाह आप को किसने दी थी?<br><br>निर्देशः कई जवाब संभव हैं, दिए गए सभी जवाब रिकार्ड करें।<br>फिर पूछें: और भी किसिने सलाह दी थी?	ASHA आशा	1	
		AWW आंगनवाड़ी कार्यकर्ता	2	
		ANM/LHV एएनएम/एलएचवी	3	
		Doctor डाक्टर	4	
		VO/SHG Member/SS ग्राम संगठन/समूह के सदस्य/स्वास्थ्य सखी	5	
		Elders in family/ friends/neighbors<br>घर के बड़े बुजुर्ग/दोस्त/पडोसी	6	
		Other (specify) अन्य (स्पष्ट करे)_______________	7	
		No one advised<br>किसिने सलाह नहीं दी थी	9	

D. INFORMATION ON DELIVERY CARE

S.N.	Questions	Coding categories	Codes	Skip to
D1	**Please provide the following information related to your last live birth?**<br>अब आप हमे अपने पिछले बच्चे के बारे में कुछ जानकारियाँ दिजिए			
(a)	In which year you delivered your last child?<br>आप का सबसे छोटा बच्चा किस साल में पैदा हुया था?	Year of delivery<br>प्रसव का वर्ष	[ ][ ][ ][ ]	
(b)	In which month? किस महीना में पैदा हुया था?	Month of delivery प्रसव का महीना	[ ][ ]	
(c)	How many months ago was your last child born?<br>**INS: Compare this answer with D1a & D1b. If response is different, find out the discrepancy and correct the responses.**<br>आपका सबसे छोटा बच्चा कितने महीना पहले पैदा हुया था?<br>निर्देश : प्रश्न **c** का जवाब **a** और **b** से मिलाये। यदि जवाब में अंतर हे तो कारण देखें और जवाब को सही करें।	Age of child (in months)<br>(if less than 1 month, write in 00)<br>बच्चे की उमर (महीनों में)	[ ][ ]	
(d)	What was the size of the baby at birth?<br>जन्म के समय बच्चा कितना बड़ा था?	Very small बहुत छोटा<br>Small छोटा<br>Normal आमतौर पे जैसा होता है<br>Quite big काफी बड़ा	1<br>2<br>3<br>4	
(e)	Was your last child a girl or a boy?<br>क्या वह लड़कि थी या लड़का ?	Male लड़का<br>Female लड़कि	1<br>2	
(f)	Place of delivery<br>प्रसव कहां हुआ था?	Sub Centre सब सेंटर/उप केन्द्र<br>PHC प्राथमिक स्वास्थ्य केन्द्र<br>CHC सामुदायिक स्वास्थ्य केन्द्र<br>District Hospital<br>जिला सरकारी अस्पताल<br>Private Hospital प्राइवेट अस्पताल<br>Home घर<br>Other (specify) अन्य (स्पष्ट करे)___________	1<br>2<br>3<br>4<br><br>5<br>6<br>7	1–5 → **D1(h)**
(g)	Who conducted the delivery at home?<br>घर पर प्रसव किसने कराया था?	Doctor डाक्टर<br>ANM/LHV/Nurse<br>एएनएम/एलएचवी/नर्स<br>Dai दाई<br>Friends/Relatives/Elder person<br>घर के बड़े बुजुर्ग/दोस्त<br>Nobody कोइ नही<br>Other (specify) अन्य, स्पष्ट करें_____________	1<br>2<br><br>3<br>4<br><br>5<br>7	→ **D1(j)**
(h)	To go to health facility for delivery did you use the 102/108 ambulance facility?<br>प्रसव के लिए अस्पताल जाने के लिए, क्या आपने 102/108 सेवा का इस्तेमाल किया था?	Yes हाँ<br>No नहीं	1<br>2	1 → **D1(j)**
(i)	What was the main reason for not using the 102/108 ambulance facility?<br><br>102/108 सेवा का इस्तेमाल न करने का मुख्य कारण क्या था?	Called, but it did not come<br>बुलाया था, पर नही आया<br>Was not aware about the service<br>इस सेवा के बारे में पता नही था<br>Arranged by self<br>खुद से व्यवस्था किरी<br>Had our own vehicle<br>खुद का वाहन था<br>It was night<br>रात का समय था<br>Other (specify) अन्य, स्पष्ट करें_____________<br>Don't Know पता नही या मालूम नही	 1<br><br>2<br><br>3<br><br>4<br><br>5<br>7<br>8	

S.N.	Questions	Coding categories	Codes	Skip to
(j)	Is the child alive?<br>क्या बच्चा जीवित है?	Yes हाँ<br>No नहीं	1<br>2	→ D2
(k)	If died, age at death<br>यदि बच्चे की मृत्यु हो गयी हो तो मृत्यु के समय बच्चे की उम्र	Day दिन .... 1<br>Month महीना....... 2	☐☐<br>☐☐	
(l)	Where did the child die?<br>बच्चे की मृत्यु कहाँ हुई?	Home घर<br>On the way while going to health facility स्वास्थ्य केन्द्र ले जाने के रास्ते में<br>On the way while coming back from health facility स्वास्थ्य केन्द्र से लौटने के रास्ते में<br>Govt. health facility सरकारी स्वास्थ्य केन्द्र में<br>Private health facility of qualified doctor प्रशिक्षित प्राईवेट डाक्टर का स्वास्थ्य केन्द्र में<br>Private health facility managed by non-qualified providers झोलाछाप गॉव के डाक्टर का दुकान/क्लीनिक में<br>Other (specify) अन्य, स्पष्ट करें__________________	1<br>2<br>3<br>4<br>5<br>6<br>7	
(m)	What was the cause of death of the baby?<br>**INS: If reports more than one reason, ask the mother about the most important reason**<br>बच्चे की मृत्यु का क्या कारण था?<br>निर्देश : अगर एक से अधिक कारण है तो, सबसे महत्वपूर्ण कारण जो माँ बताती है वह रिकार्ड करें	Tetanus टिटनस<br>Congenital abnormality जन्मजात विकृतियां<br>Pre-term birth (born early) समय से पहले जन्म हुआ था<br>Low birth weight (born small in size) जन्म के समय कम वजन/बहुत ही छोटा था<br>Not able to cry or breath or suckle after birth (Birth asphyxia) जन्म के बाद नही रोया/साँस नही लिया (जन्म श्वासवरोध)<br>Birth injury जन्म के समय घायल होना<br>Fever or cold/unresponsive बुखार या ठंड लगना/सुस्त होना<br>Redness or drainage from umbilical cord stump नाल के चारों ओर लाली और मवाद निकलना<br>Pneumonia /lower chest wall/ribs pulled while breathing निमोनिया/पसली चलना<br>Diarrhoea or blood in stool दस्त/डायरिया या पखाना/मल में खून आना<br>Yellow palms or soles (jaundice) हथेलिया या तलवा का पीला होना (पीलिया होना)<br>Sepsis/ ulcers, abscess or sores skin rash घाव का सड़ना/ अल्सर, फोड़ा या त्वचा में लाल चकत्ते होना<br>Convulsions/fits फिट्स आना<br>Bulging fontanelle on the head खोपड़ी पर टेटन/उभाड़ होना<br>Accident दुर्घटना<br>Jamoga जमोगा<br>Other (specify) अन्य, स्पष्ट करें__________________	01<br>02<br>03<br>04<br>05<br>06<br>07<br>08<br>09<br>10<br>11<br>12<br>13<br>14<br>15<br>77	

S.N.	Questions	Coding categories	Codes	Skip to
**INS: Check D1f, if code is 1 to 5 (INSTITUTIONAL DELIVERY) ask D2 to D4**				
D2	How long did you stay in the health facility after the delivery?<br>प्रसव के बाद आप कितने समय तक स्वास्थ्य केन्द्र में रही थीं?	Hours घंटे...... 1<br>Days दिन...... 2		
D3	After delivery who suggested you to go back home? Was it your family member or health care provider or ASHA?<br><br>प्रसव के बाद किसने आपको घर जाने की सलाह दी – परिवार के सदस्य या स्वास्थ्य केन्द्र के स्वास्थ्यकर्ता या आशा ने?	Family members<br>परिवार के सदस्य<br>Health care provider<br>स्वास्थ्य केन्द्र के स्वास्थ्यकता<br>ASHA आशा<br>Self स्वयं	1<br><br>2<br>3<br>4	
D4	Was any health advice given to you before or at the time of discharge? If yes, what?<br>**Probe: Anything else?**<br>**INS: Multiple answers possible. Code all given responses.**<br><br>स्वास्थ्य केन्द्र से छुटटी मिलने के पहले या उस समय आपको कोइ सलाह दी गयी थी, अगर हाँ तो क्या?<br><br>फिर पुछें. : और भी कुछ कहा था?<br>निर्देश : कई जवाब संभव है। दिये गये सभी जवाब रिकार्ड करें।	Early breastfeeding and not to give any pre-lacteal<br>जन्म के तुरंत बाद स्तनपान कराना और अन्य कुछ भी नही खिलाना व पिलाना<br>Keep the baby warm by putting the naked baby on bare chest and cover with clothes<br>बच्चे को गर्म रखने के लिए नंगे शिशु को खुली हुई छाती से लगाकर रखना और कपडे से ढकना<br>Delay first bathing by at least 2 days<br>नवजात शिशु को कम से कम दो दिन तक नही नहलाना<br>Not to apply any thing to cord stump<br>नाल पर कुछ नहीं लगाना<br>To get mother and newborn checked up within 7 days after delivery either at facility or at home even if there is no problem<br>कोई समस्या न हो तो भी प्रसव के बाद सात दिनों के भीतर माँ और नवजात की स्वास्थ्य केन्द्र या घर पर जाँच करवाना<br>Other (specify) अन्य (स्पष्ट करें)________<br>No advice was given<br>कोई भी सलाह नहीं दी गई	1<br><br>2<br><br>3<br><br>4<br><br>5<br><br>7<br><br>9	
**INS: Check D1f, if code is 6 (HOME DELIVERY) ask D5 & D6**				
D5	Why did you prefer to deliver at home and not at a health facility even though Govt. is paying money to deliver at health facility?<br><br>**Probe: Any other reason?**<br>**INS: Multiple responses possible. Code up to 4 responses.**<br><br>स्वास्थ्य केन्द्र में प्रसव के लिए सरकार पैसे देती है फिर भी स्वास्थ्य केन्द्र की बजाय घर में प्रसव करवाने के पक्ष में निर्णय किन कारण से लिया गया था?<br><br>फिर पूछें : क्या और कोई कारण था?<br><br>निर्देश : कई जवाब संभव है। 4 जवाब तक रिकार्ड करें	Wanted institutional delivery but child just happened at home<br>स्वास्थ्य केन्द्र में जाना चाहते थे पर बच्चा घर में हो गया<br>Family tradition/ Did not go for previous deliveries also<br>पारिवारिक परंपरा /पिछले प्रसवों में भी स्वास्थ्य केन्द्र नहीं गयी थी<br>It was elders' decision यह बुजुर्गों का निर्णय था<br>Delivery was normal प्रसव सामान्य था<br>Pain started at night so had no option<br>दर्द रात में शुरू हुआ था इसलिए कोई उपाय/चारा नहीं था<br>Could not arrange transportation in time<br>समय पर यातायात का इंतजाम नहीं हो सका था<br>Did not have required funds/could not arrange required fund<br>आवश्यक पैसे नहीं थे /इंतजाम नहीं हो सका<br>No one at home to look after children<br>घर में बच्चों की देखभाल करने वाला कोई नहीं था<br>No health facility close by<br>नजदीक में कोई स्वास्थ्य केन्द्र नहीं है<br>Institutional delivery costs too much<br>स्वास्थ्य केन्द्र में प्रसव कराने मे बहुत पैसा खर्च होता है<br>Other (specify) अन्य (स्पष्ट करें)____________	01<br><br>02<br>03<br>04<br>05<br>06<br>07<br>08<br>09<br>10<br>77	

S.N.	Questions	Coding categories	Codes	Skip to
D6	After delivery has anyone given advice on postnatal care of mother and newborn? If yes, what advice was given?<br>प्रसव के बाद क्या किसी ने आपको माँ और बच्चें के स्वास्थ्य सबंधी कुछ सलाह दी थी? अगर हाँ तो क्या क्या सलाह दी थी?<br>**INS: Circle all the spontaneous answer recived. Then probe one by one all that were not mentioned**<br>निर्देश : पहले वह जवाब जो माँ बताती है वह रिकाड़ करें । फिर एक एक कर के बाकी के सवाल पूछें	**Circle all the Spontanious responses**<br>खुद से बताया	**After Probing**<br>पूछने पर बताया<br>**YES**	**NO**
a)	Early breastfeeding<br>जन्म के तुरंत बाद स्तनपान कराना	1	1	2
b)	Keep the baby warm by putting the naked baby on bare chest and cover with clothes<br>बच्चे को गर्म रखने के लिए नंगे शिशु को खुली हुई छाती से लगाकर रखना और कपडे से ढकना	2	1	2
c)	Delay first bathing by at least 2 days<br>नवजात शिशु को कम से कम दो दिन तक नही नहलाना	3	1	2
d)	Not to apply any thing to cord stump<br>नाल पर कुछ नहीं लगाना	4	1	2
e)	To get mother and newborn checked up within 7 days after delivery either at facility or at home even if there is no problem<br>अगर कोई समस्या न हो तो भी प्रसव के बाद सात दिनों के भीतर माँ और नवजात शिशु की स्वास्थ्य केन्द्र या घर पर जाँच करवाना	5	1	2
**INS: Ask D7 to D9 to ALL WOMEN, irrespective of place of delivery**				
D7	What problems/complications can a woman face during pregnancy or delivery or within 42 days of delivery which requires immediate medical attention?<br>**DO NOT READ RESPONSES**<br>**Probe: Any other problem?**<br>**INS: Multiple responses possible. Code all that apply.**<br>वह कौन कौन सी जटिल्ताऐं है जो गर्भावस्था के दौरान या प्रसव के दौरान या प्रसव के 42 दिनो के अंदर हो सकती है और जिन के लिए स्वास्थ्य केन्द्र जाना चाहिए या किसी स्वास्थ्यकर्ता से तुरंत सम्पर्क करना चाहिए?<br>निर्देश : उत्तरों को न पढ़ें।<br>फिर पुछें : इनके अलावा कोई अन्य खतरा हो सकता हैं?<br>निर्देश : कई जवाब संभव हैं। दिए गए सभी जवाब रिकार्ड करें।			
	Severe headache /High blood pressure तेज़ सिर दर्द या ज़्यादा ब्लड प्रेशर होना		01	
	Blurred vision/Convulsions धुंधला दिखना,दौरे पडना		02	
	Absence or /less movements of fetus पटे में बच्चे की हिल डोल में कमी		03	
	Prolonged labor over 12 hours प्रसव दर्द 12 घण्टे से ज़्यादा होना		04	
	Excessive vaginal bleeding योनि से बहुत ज़्यादा खून बहना		05	
	Foul smelling discharge योनि से बदबूदार स्राव होना		06	
	Delay in placental expulsion/Retained placenta<br>बच्चा होने के तीस मिनट बाद तक खेंडी/आँवल का बाहर ना आना व अन्दर रह जाना		07	
	Severe abdominal pain पेट में जोर का दर्द होना		08	
	Rupture uterus बच्चेदानी का फटना		09	
	Baby in abnormal position बच्चे का असामान्य स्थिति में होना		10	
	Cord prolapsed/Baby's hand & feet coming out first<br>बच्चा होने से पहले ही नाल का बाहर आना/बच्चे का हाथ पैर पहले बाहर आना		11	
	Cord around neck नाल का गर्दन में फसना		12	
	High fever तेज बुखार होना		13	
	Other (specify)____________________ अन्य, स्पष्ट करें		77	
	Do not know मालूम नहीं		88	

S.N.	Questions	Coding categories	Codes	Skip to
D8	Did you face any complication during your last pregnancy or delivery or within 42 days of delivery?<br>क्या आपको पिछली गर्भावस्था या प्रसव के दौरान या प्रसव के बाद 42 दिनो के अंदर किसी जटिल्ता / कठिनाई का सामना करना पड़ा था?	Yes हाँ<br>No नहीं	1<br>2	 → E1
D9	What complications did you face during your last pregnancy or delivery or within 42 days of delivery?<br>**DO NOT READ RESPONSE**<br>**Probe: Any other complications?**<br>**INS: Multiple responses possible. Code all given responses.**<br>पिछली गर्भावस्था या प्रसव के दौरान या प्रसव के बाद 42 दिनो के अंदर आपको कौन कौन सी जटिलताओं का सामना करना पड़ा था?<br>निर्देश : उत्तरों को न पढ़ें।<br>फिर से पुछे : इनके अलावा किसि अन्य जटिल्ता या कठिनाई का सामना करना पड़ा था?<br>निर्देश : कई जवाब संभव है, दिए गए सभी जवाब रिकार्ड करें।			
		Severe headache /High blood pressure तेज़ सिरदर्द या ज़्यादा ब्लड प्रेशर होना	01	
		Blurred vision/Convulsions धुंधला दिखना, दौरे पडना	02	
		Absence or /less movements of fetal बच्चे की हिल डोल मैं कमी	03	
		Prolonged labor over 12 hours प्रसव दर्द 12 घण्टे से ज़्यादा होना	04	
		Excessive vaginal bleeding योनि से बहुत ज़्यादा खून बहना	05	
		Foul smelling discharge योनि से बदबूदार स्राव होना	06	
		Delay of more than 30 minutes in placental expulsion/Retained placenta बच्चा होने के तीस मिनट बाद तक खेंडी/आँवल का बाहर आना ना आना व अन्दर रह जाना	07	
		Severe abdominal pain पेट में जोर का दर्द होना	08	
		Rupture uterus बच्चेदानी का फटना	09	
		Baby in abnormal position बच्चे का असामान्य स्थिति में होना	10	
		Cord prolapsed/Baby's hand & feet coming out first बच्चा होने से पहले ही नाल का /बच्चे का हाथ पैर पहले बाहर आना	11	
		Cord around neck नाल का गर्दन में फसना	12	
		High fever तेज़ बुखार होना	13	
		Other (specify)__________________ अन्य, स्पष्ट करें	77	
		Do not know मालूम नहीं	88	
**CHECK D9. if experienced complication, i.e. if code 01/04/05/10 is marked, ask D10 to D13, else go to E1**				
D10	How long after the 1st symptom did you decide to seek care?<br>पहले जटिलता के लक्षण दिखने के कितनी देर के बाद इलाज कराने का निर्णय लिया गया?	Hours घंटे……1<br>Did not seek care कोइ इलाज नहीं करवाया…5	☐☐	 → E1
D11	How long after the decision to take you to health facility, a transport was arranged?<br>इलाज करवाने का निर्णय लेने की कितनी देर बाद यातायात के साधन का इंतेजाम हुआ?	Hours घंटे<br>Did not require transportation साधन का ज़रूरत नहीं पड़ा	☐☐<br>55	
D12	For the treatment expenses, did you have sufficient money with you or had to make arrangements for it at that time?<br>इलाज के खर्चे के लिए क्या आपके पास र्प्याप्त पैसा था या उसी समय इंतेजाम करना पड़ा?	Had sufficient money पर्याप्त पैसे थे<br>Arranged at that time उसी समय इंतेजाम किये	1<br>2	
D13	Did you receive the required treatment at the first place you visited or you were referred elsewhere?<br>जहाँ आप इलाज के लिए सबसे पहले गयीं थीं क्या आपका इलाज वहाँ हो गया या आपको कहीं और रेफर किया गया ?	Received treatment at first place where visited इलाज वहीं हुआ जहाँ इलाज के लिए सबसे पहले गयीं थीं<br>Refered elsewhere कहीं और रेफर किया गया	1<br>2	

E. POST NATAL CARE

S.N.	Questions	Categories	Codes	Skip to
E1	Did you face any complications within 42 days after delivery?<br><br>प्रसव के 42 दिनो के अन्दर क्या आपको स्वास्थ्य सबंधी कोई समस्या/जटिलता का सामना करना पड़ा था?	Yes हाँ<br>No नहीं	1<br>2	 → **E3**

S.N.	A. What complications did you face?<br>**INS: Do not read responses Multiple responses possible. Code up to 3 responses.**<br>आपको कौन कौन सी स्वास्थ्य संबंधी समस्याओं/जटिलताओं का सामना करना पड़ा था?<br>निर्देश : दिय गये जवाबों को न पढ़ें। कई जवाब संभव है। 3 जवाब तक रिकार्ड करें।	**Codes**	B. Which place you went for treatment and for how long? (day/days):<br>आप इलाज के लिये कहाँ कहाँ गये और वहाँ पर कितने दिन तक इलाज चला? सब से पहले कहाँ गई थी? उसके बाद कहाँ?					
			1st treatment		*2nd treatment*		*3rd treatment*	
			*Place**	*# of Days*	*Place**	*# of Days*	*Place**	*# of Days*
E2	a) Excessive vaginal bleeding<br>योनि से बहुत ज़्यादा खून बहना	1						
	b) Convulsions/ Blurred vision /severe headache/high BP<br>धुंधला दिखाई देना/तेज़ सिर दर्द/उच्च रक्तचाप	2						
	c) Pus or foul smelling vaginal discharge<br>योनि से पस या बदबूदार स्राव निकलना	3						
	d) High fever तेज़ बुखार	4						
	e) Other (specify)<br>अन्य (स्पष्ट करें)_______________	7						

***Place of treatment:**
1=Sub Centre सब सेंटर/उप केन्द्र
2=PHC/CHC प्राथमिक/सामुदायिक स्वास्थ्य केंद्र
3= District hospital जिला सरकारी अस्पताल
4=Private hospital/ doctors प्राइवेट अस्पताल/डाक्टर
5= Unqualified village doctor गाँव के झोलाछाप डाक्टर
6=Home treatment घर का इलाज
7= Other (specify) अन्य (स्पष्ट करे)_____
9= No treatment कोइ इलाज नहीं

<table>
<tr><th>S.N.</th><th>Questions</th><th>Coding categories</th><th>Codes</th><th>Skip to</th></tr>
<tr><td>E3</td><td>Did you receive a post natal check-up within 42 days after delivery?
क्या आपने प्रसव के 42 दिनों के भीतर अपनी जाँच करवाइ थी?</td><td>Yes हाँ
No नहीं</td><td>1
2</td><td>
→ E7</td></tr>
<tr><td>E4</td><td>How many times, did you receive the postnatal check-up within 7 days of delivery?
आपने प्रसव के सात दिनों के भीतर कुल कितनी बार जाँच करवाइ थी?</td><td></td><td>☐</td><td></td></tr>
<tr><td>E5</td><td>INS: Ask for the first three PNC that she received within 42 days of delivery.

निर्देश : प्रसव के 42 दिनों के भीतर की तीन जाँचों के बारे में पूछे।</td><td>A) How many day/days after your last delivery did you receive this PNC?
प्रसव के कितने दिन बाद यह जाँच करवाइ थी?</td><td colspan="2">B) Who did the check up*

जाँच किसने करी थी?</td></tr>
<tr><td>A</td><td>First PNC - पहली जाँच</td><td>☐☐</td><td colspan="2">☐</td></tr>
<tr><td>B</td><td>Second PNC - दुसरी जाँच</td><td>☐☐</td><td colspan="2">☐</td></tr>
<tr><td>C</td><td>Third PNC - तीसरी जाँच</td><td>☐☐</td><td colspan="2">☐</td></tr>
<tr><td></td><td></td><td colspan="3">* Codes for who did the check-up
1 = ASHA आशा
2= AWW आंगनवाड़ी कार्यकर्त्री
3= ANM एएनएम
4= Provider from Govt. hospital सरकारी असपताल के किसी प्रदाता से
5= Private Doctor/Nurse प्राइवेट डाक्टर/नर्स
7= Other (specify) अन्य (स्पष्ट करे)________</td></tr>
<tr><td>E6</td><td>What examinations were done in post-natal check ups for you?
INS: Multiple responses possible. Code all given responses.
प्रसव के बाद आपकी कौन कौन सा जाँच हुइ थी?

निर्देश : कई जवाब संभव है। दिये गये सभी जवाब रिकार्ड करें।</td><td>Temperature check-up तापमान की जाँच
BP check-up रक्तचाप की जाँच
Vaginal bleeding check-up योनि से रक्तस्राव की जाँच
Examination of breast problem स्तन की जाँच
Other (specify)____________अन्य, स्पष्ट करे
None कोई जाँच नही</td><td>01
02
03
04
77
55</td><td></td></tr>
<tr><td>E7</td><td>What examinations were done in post-natal check ups for the newborn?
INS: Multiple responses possible. Code all given responses.

प्रसव के बाद नवजात शिशु की कौन कौन सी जाँच हुइ थी ?

निर्देश : कई जवाब संभव है। दिये गये सभी जवाब रिकार्ड करें।</td><td>Temperature check-up तापमान की जाँच
Cord check-up नाल की जाँच
Eye check-up आँख की जाँच
Limbs check-up हाथ और पैर की जाँच
Breastfeeding check-up स्तनपान की जाँच
Position and attachment of breastfeeding स्तनपान के तरिके की जाँच
Check number of times urine passed पेशाब करने की संख्या की जाँच
Weight checkup वजन की जाँच
Other (specify)_________ अन्य, स्पष्ट करें
None कोई जाँच नहीं</td><td>01
02
03
04
05
06
07
08
77
55</td><td></td></tr>
<tr><td>E8</td><td>Does a mother need a checkup after delivery, even if she is feeling fine?

क्या एक माँ को प्रसव के बाद कोई जाँच करवाने की जरूरत है?चाहे वह स्वस्थ हो और अच्छा महसूस कर रही हो?</td><td>Yes हाँ
No नहीं
Do not know मालूम नहीं</td><td>1
2
8</td><td>
2, 8 → E10</td></tr>
</table>

E9	How many checkups should a mother undergo within 7 days of delivery, even if she is feeling fine?<br><br>प्रसव के सात दिनों के भीतर एक माँ को कितनी बार जाँच करवानी चहिये, चाहे उसे कोई भी तकलीफ ना हो?	Do not know<br>मालूम नहीं	☐<br><br>8	

E10	a) Did any of the following ------- contact you or did you meet _________within 7 days of delivery?<br><br>प्रसव के सात दिन के अन्दर क्या .... ने आपसे या आपने .... से सम्पर्क किया?	**Yes** हाँ	**No** नही	b) If yes, how many times such contact been made within 7 days of delivery?<br><br>अगर हाँ, तो प्रसव के सात दिनों के अन्दर, कुल कितनी बार ऐसे सम्पर्क हुऐ?
A	ANM एएनएम	1	2	☐
B	ASHA आशा	1	2	☐
C	AWW आंगनवाड़ी कार्यकर्त्री	1	2	☐
D	VO/SHG Member/SS<br>ग्राम संगठन/समूह के सदस्य/स्वास्थ्य सखी	1	2	☐

E11 What health problems/ complications, a newborn can face within first month of birth?
INS: Multiple responses possible. Code all given responses.
Probe: Any other complications?

जन्म के बाद एक महीने के अन्दर नवजात शिशु को स्वास्थ्य संबंधी कौन–कौन सी समस्याओं/जटिलताओं का सामना करना पड़ सकता है?

निर्देश : कई जवाब संभव हैं। दिए गए सभी जवाब रिकार्ड करें।
फिर पूछें : इनके अलावा कोई अन्य समस्याओं/जटिलताओं का सामना करना पड़ सकता है?

Poor sucking or feeding/ Breast feeding<br>अच्छी तरह से दूध नहीं चूस पाना/स्तनपान में परेशानी होना	01	
Redness and discharge around cord stump/ Umbilical sepsis<br>नाल के चारों ओर लाली और स्राव निकलना	02	
Difficult or fast breathing सँास लेने में कठिनाई या तेज़ सँास लेना	03	
Pneumonia/chest indrawing निमोनिया/पसली चलना	04	
Shivering बच्चे का ठण्डा होना/काँपना	05	
High Fever तेज़ बुखार होना	06	
Baby becomes drowsy/ Unconscious बच्चे का सुस्त होना/ बेहोश होना	07	
Baby very small/low birth weight बहुत छोटा बच्चा पैदा होना या कम वजन का होना	08	
Diarrohea डायरिया	09	
Baby does not cry immedietly after birth जन्म के तुरंत बाद बच्चे का ना रोना	10	
Jaundice पीलिया होना	11	
Jamoga जमोगा होना	12	
Other (specify) अन्य (स्पष्ट करें)________	77	
Do not know नहीं जानती	88	→ **E13**

E12	Who informed you about these complications?<br>**INS: Multiple responses possible. Code all given responses.**<br><br>इन जटिलताओं की जानकारी आपको किसने दी?<br>निर्देश : कई जवाब संभव हैं, दिए गए सभी जवाब रिकार्ड करें	ASHA आशा	1	
		AWW आंगनवाड़ी कार्यकर्ता	2	
		ANM/LHV एएनएम/एलएचवी<br>Doctor डाक्टर	3	
		VO/SHG Member/SS<br>ग्राम संगठन/समूह के सदस्य/स्वास्थ्य सखी	4	
		Elders in family/ friends/neighbors	5	
		घर के बड़े बुजुर्ग/दोस्त/पडोसी	6	
		Other (specify) अन्य (स्पष्ट करे)______	7	
		No one/self-experience<br>कोई नही/खुद अनुभव किया	9	

E13	Did your last child face any health complications within first month of birth?<br><br>जन्म के एक महीने के अन्दर क्या आपके पिछले बच्चे को किसी स्वास्थ्य संबंधी समस्या /जटिलता का सामना करना पड़ा था?	Yes हाँ<br>No नहीं	1<br>2 →	F1

E14

A. What complications did your last child face within **first month of birth**?<br>**INS: Do not read responses**<br>**Multiple responses possible. Code up to 3 responses.**<br>आपके पिछले बच्चे को जन्म के एक महीने के अन्दर कौन कौन सी स्वास्थ्य समस्यायें या जटिलतायें हुई थी?<br>निर्देश : दिय गये जवाब को ना पढ़ं। कई जवाब संभव है। 3 जवाब तक रिकार्ड करें	*Codes*	**B. For each complication that the child faced, ask the sequence of all treatment (places and time)?**<br>Which place you did you take him/her for treatment and for how long? (day/days):<br><br>बच्चे को जो जो समस्यायें/जटिलतायें हुइ थीं उसकें बारे में एक एक करके इलाज की जगह और कितने दिन तक इलाज चला था वह पुछे<br>बच्चे के इलाज के लिये आप कहाँ कहाँ गये थे और वहाँ पर कितने दिन तक इलाज चला था?					
		1st treatment		*2nd treatment*		*3rd treatment*	
		Place*	# of Days	Place*	# of Days	Place*	# of Days
a) Redness and discharge around the cord stump/ Umbilical sepsis<br>नाल के चारों ओर लाली और स्राव निकलना	1						
b) Difficult or fast breathing<br>साँस लेने में कठिनाई या तेज़ साँस लेना	2						
c) Pneumonia/chest indrawing<br>निमोनिया/पसली चलना	3						
d) Shivering<br>बच्चे का ठण्डा होना/काँपना	4						
e) High Fever तेज़ बुखार होना	5						
f) Baby became drowsy/ Unconscious<br>बच्चे का सुस्त होना, बेहोश होना	6						
g) Diarrohea डायरिया	7						
h) Jaundice पीलिया	8						
i) Other (specify)<br>अन्य (स्पष्ट करें)________________	9						

***Place of treatment:**
1=Sub Centre उप केन्द्र/ सब सेंटर
2=PHC /CHC प्राथमिक/सामुदायिक स्वास्थ्य केंद्र
3= District hospital जिला सरकारी अस्पताल
4=Private hospital/ doctors प्राइवेट अस्पताल/डाक्टर
5= Unqualified village doctor गाँव के डाक्टर/झोला छाप डाक्टर
6=Home treatment घर का इलाज
7= Other (specify) अन्य (स्पष्ट करे)_____
9= No treatment कोइ इलाज नहीं

	**E15 TO E17 - APPLICABLE ONLY IF CHILD HAS FALLEN SICK AND TREATED (CHECK E13 AND E14)**			
E15	How long after the 1st symptom was noticed, it was decided to seek care of your baby?<br>पहले जटिलता के लक्षण दिखाने के बाद यह तय करने में कितना समय लगा कि बच्चे का इलाज करना है?	Hours घंटे .....1<br>Days दिन.......2	☐☐<br>☐☐	

E16	How long after the decision to take your baby to health facility, a transport was arranged?<br>इलाज करना तय होने के बाद, यातायात के साधन का इंतेजाम करने में कितना समय लगा?	Hours घंटे.....1<br>Minute मिनट.....2<br>Did not require transportation..5<br>साधन का ज़रूरत नहीं पड़ा	[ ][ ]<br>[ ][ ]	
E17	For the treatment expenses, did you have sufficient money with you or had to make arrangements for it at that time?<br>इलाज के खर्चे के लिए क्या आपके पास पर्याप्त पैसा था या उसी समय इंतेजाम करना पड़ा था?	Had sufficient money<br>पर्याप्त पैसे थे<br>Arranged at that time<br>उस वक्त इंताजाम किये	1<br>2	

F. CORD CARE

S.N.	Questions	Coding categories	Codes	Skip to
F1.	What did you do to prevent cord infectionof your last baby?<br>**Probe: Any other care?**<br>**INS: Multiple responses possible. Code all given responses.**<br>बच्चे की नाभि को पकने और संक्रमण से बचाने के लिए आप ने क्या क्या किया था?<br>फिर पूछें : क्या इसके साथ कोई अन्य देखभाल भी की थी ?<br>निर्देश : कई जवाब संभव हैं। दिए गए सभी जवाब रिकार्ड करें।	Used new blade to cut cord<br>नाल काटने के लिए नये ब्लेड का उपयोग किया<br>Used boiled thread to tie cord<br>नाल को बांधने के लिए उबले धागे का उपयोग किया<br>Nothing applied on cord<br>नाभि पर कोई चीज नहीं लगायी<br>Kept cord stump clean and dry<br>नाभि को साफ और सूखा रखा<br>Applied Oil/Ghee<br>तेल/घी लगाया<br>Applied Talcum powder/Ash/Turmeric<br>टैल्कम पाउडर/राख/हल्दी लगायी<br>Applied antiseptic cream<br>एंटीसेप्टिक क्रीम/घोल/क्रीम लगायी<br>Applied Gentian violet<br>जेंशियन वायलेट (नीली दवा) लगायी<br>Other (specify)__________<br>अन्य, स्पष्ट करें	01<br>02<br>03<br>04<br>05<br>06<br>07<br>08<br>77	
F2.	How to prevent cord infection to a newborn after birth?<br>**Probe: Any other care which needs to be taken?**<br>**INS: Multiple responses possible. Code all given responses.**<br>नवजात शिशु की नाभि को पकने और संक्रमण से बचाने के लिए क्या करना चाहिए ?<br>फिर पूछें : क्या किसी अन्य देखभाल की भी ज़रूरत होती है?<br>निर्देश : कई जवाब संभव हैं। दिए गए सभी जवाब रिकार्ड करें।	Use new blade to cut cord<br>नाल काटने के लिए नये ब्लेड का उपयोग करना चाहिए<br>Use boiled thread to tie cord<br>नाल को बांधने के लिए उबला धागा का उपयोग करना चाहिए<br>Nothing should be applied<br>नाभि पर कोई चीज नहीं लगाना चाहिए<br>Keep cord stump clean and dry<br>नाभि को साफ और सूखा रखना चाहिए<br>Oil/Ghee should be applied<br>तेल/घी लगाना चाहिए<br>Talcum powder/Ash/Turmeric should be applied<br>टैल्कम पाउडर/राख/हल्दी लगानी चाहिए<br>Antiseptic cream should be applied<br>एंटीसेप्टिक क्रीम/घोल/क्रीम लगानी चाहिए<br>Gentian violet should be applied<br>जेंशियन वायलेट (नीली दवा) लगायी जानी चाहिए<br>Other (specify)__________<br>अन्य, स्पष्ट करें<br>Do not know नहीं जानती	01<br>02<br>03<br>04<br>05<br>06<br>07<br>08<br>77<br>88 →	**G1**

S.N.	Questions	Coding categories	Codes	Skip to
F3.	Who suggested you?<br>**INS: Multiple responses possible. Code up to 3 responses.**<br>**Probe: Who else suggested this?**<br>इसकी सलाह किसने दी थी?<br>फिर से पुछें: क्या किसी और ने भी सलाह दी थी?<br>निर्देश : कई जवाब संभव है। तीन जवाब तक कोड करें।	ASHA आशा<br>AWW आंगनवाड़ी कार्यकर्ता<br>ANM/LHV एएनएम/एलएचवी<br>Doctor डाक्टर<br>VO/SHG Member/SS ग्राम संगठन/समूह के सदस्य/स्वास्थ्य सखी<br>Elders in family/ friends घर के बड़े बुजुर्ग/दोस्त<br>Other (specify) अन्य (स्पष्ट करे)________<br>No one कोई नहीं	1<br>2<br>3<br>4<br>5<br>6<br>7<br>9	

G. THERMAL CARE

S.N.	Questions	Coding categories	Codes	Skip to
G1.	After the birth of your last child, when was the child given bath for the first time?<br>आपके पिछले बच्चे के जन्म के कितने समय बाद उसे पहली बार नहलाया गया था?	Hours घंटे……1<br>Child died बच्चा प्रसव के बाद जल्द ही मर गया…....0	__ __	
G2.	What was done to clean the child immedietly after birth?<br>बच्चे को जन्म के तुरंत बाद उसे साफ करने के लिए क्या किया गया था?	Applied mustard oil and cleaned with dry cloth सरसों का तेल लगाया और सूखे कपड़े से साफ किया<br>Cleaned with dry cloth only सिर्फ सूखे कपड़े से साफ किया<br>Applied mustard oil and gave bath with water and soap सरसों का तेल लगाया और पानी तथा साबुन से नहलाया<br>Gave bath with water and soap only केवल पानी तथा साबुन से नहलाया<br>Other (specify)_______अन्य, स्पष्ट करें	1<br>2<br>3<br>4<br>7	
G3.	What was done to keep the baby warm?<br>**Probe: anything else?**<br>**INS: Multiple responses possible. Code up to 3 responses.**<br>बच्चे को गर्म रखने के लिए क्या किया गया था ?<br>फिर पूछें : और क्या किया गया था ?<br>निर्देश : कई जवाब संभव हैं। दिए गए तीन जवाब रिकार्ड करें।	Covered/wrapped baby in clean cloth बच्चे को साफ कपड़े से लपेटा गया<br>Covered/wrapped baby in warm cloth बच्चे को गर्म कपड़े से लपेटा गया<br>Covered/wrapped baby in cotton cloths बच्चे को सुती कपड़े से लपेटा गया<br>Skin to skin contacts /KMC कंगारू विधी से बच्चे को गर्म रखा गया<br>Kept room warm with fire कमरे को आग से गर्म रखा गया<br>Incubeter/warmer machine गर्म रखने की मशीन में रखा गया<br>Other (specify)_______अन्य, स्पष्ट करें<br>Nothing was done कुछ नही किया गया	01<br>02<br>03<br>04<br>05<br>06<br>77<br>55	 <br><br>04 → **G6**
G4.	Did you practice KMC / skin to skin contact method to keep the baby warm?<br>बच्चे को गर्म रखने के लिए क्या आपने कंगारू विधी या त्वचा से त्वचा संपर्क विधी का इस्तेमाल किया था?	Yes हाँ<br>No नहीं	1<br>2	1 → **G6**

S.N.	Questions	Coding categories	Codes	Skip to
G5.	To keep child warm, KMC / skin to skin contact method is done. Do you know about this method?<br>बच्चे को गर्म रखने के लिए कंगारू विधी या त्वचा से त्वचा संपर्क विधी का इस्तेमाल किया जाता है।<br>क्या आप इस विधी को जानती है?	Yes हाँ<br>No नहीं	1<br>2	 → H1
G6.	Could you explain how to practice KMC/ skin to skin contact method?<br><br>बच्चे को गर्म रखने के लिए कंगारू विधी या त्वचा से त्वचा संपर्क विधी को कैसे इस्तेमाल किया जाता है?	Put naked child on the bare chest and covered properly with cloth so that direct skin to skin contact takes place<br>नंगे बच्चे को खुली हुई छाती पर रख कर कपड़े से अच्छी तरह से ढका ताकि त्वचा से त्वचा का सीधा संपर्क हो सके<br>Put the child on mother chest with blouse and covered the child<br>बच्चे को ब्लाउज पहने हुए अपनी छाती पर रखा और ढक लिया<br>Other (specify)_______अन्य, स्पष्ट करें	1<br><br>2<br>7	
G7.	Who suggested you?<br><br>**Probe: Who else suggested this?**<br>**INS: Multiple responses possible.**<br>**Code up to 3 responses**<br><br>इसकी सलाह किसने दी थी?<br><br>फिर से पुछें: क्या किसी और ने भी सलाह दी थी?<br>निर्देश : कई जवाब संभव है। तीन जवाब तक कोड करें	ASHA आशा<br>AWW आंगनवाड़ी कार्यकर्ता<br>ANM/LHV एएनएम/एलएचवी<br>Doctor डाक्टर<br>VO/SHG Member/SS ग्राम संगठन/समूह के सदस्य /स्वास्थ्य सखी<br>Elders in family/ friends<br>घर के बड़े बुजुर्ग/दोस्त<br>Other (specify) अन्य (स्पष्ट करे)_____<br>No one कोई नहीं	1<br>2<br>3<br>4<br><br>5<br>6<br>7<br>9	

H. BREASTFEEDING, IMMUNIZATION AND DIARRHEA CARE

S.N.	Questions	Coding categories	Codes	Skip to
H1	In case of your last child, how much time after delivery did you put the child to breast for the first time and tried to breastfeed?<br>आपने पिछले बच्चे को जन्म के कितने समय के बाद पहली बार आपने अपना दूध पिलाया था?	**Hours** घंटे<br><br>Never breastfeed<br>कभी नही पिलाया	☐☐<br><br>**55**	
H2	How much time after birth should a child be put to breast milk?<br><br>बच्चे को जन्म के कितनी देर बाद माँ का पहला दूध दिया जाना चाहिए?	**Hours** घंटे<br><br>Do not know<br>नहीं जानती	☐☐<br><br>**88**	
H3	Did you face any difficulty in breastfeeding? If yes, what difficulty did you face?<br>**Probe: Any other difficulty?**<br>**INS: Multiple responses possible.**<br>**Code up to 3 responses.**<br>बच्चे को स्तनपान कराने में क्या आपको कोई तकलीफ हुई? यदि हाँ तो आपको कौन कौन सी तकलीफ हुई?<br>फिर से पुछें: क्या किसी और तकलीफ भी हुई थी?<br>निर्देश : कई जवाब संभव है। तीन जवाब तक कोड करें।	Cracked or sore nipples/ engorgement<br>निप्पल का फट जाना या जख्म होना<br>Flat/inverted nipples<br>निप्पल का उलटा होना<br>Baby not sucking properly<br>बच्चें का ठीक से नही चूस पाना<br>Not enough breastmilk<br>दूध काफी नही होना<br>Not enough time to feed child<br>बच्चें को दूध पिलाने के लिए समय की कमी<br>Other (specify)___________ अन्य,स्पष्ट करें<br>No difficulty कोई समस्या नहीं	1<br><br>2<br><br>3<br><br>4<br><br>5<br>7<br>9	

ASK H3 TO H5 ONLY IF CHILD IS ALIVE (D1J=1), ELSE GO TO H6				
H4	Are you currently breastfeeding?<br>क्या आप वर्तमान में स्तनपान करा रही हैं?	Yes हाँ<br>No नहीं	1<br>2	
H5	**Now, I would ask about food/water given to your last child.**<br>At what age did you start feeding the following to your youngest child?<br><br>अब मैं आपसे आप का सबसे छोटे बच्चे के खान–पान के बारे में पूछूंगी।<br>कृपया बतायें कि बच्चे को किस उम्र से ....खिलाना/पिलाना शुरू किया गया था?			
a.	At what age did you start giving water to the child?<br>बच्चा कितने महीनों का था जब बच्चे को पानी देना शुरू किया?	Age of child in month उस समय बच्चे कि उम्र महीना में<br>**Ins: Code 00 if less than one month**<br>अगर एक माह से कम हो तो 00 कोड करें<br>Not yet started अभी शुरू नहीं किया है	□□<br>55	
b.	At what age did you start giving animal milk (goat, cow or buffalo) / formula or powdered milk to the child?<br>जानवर (बकरी, गाय या भैंस) का दूध/फार्मूला या पाउडर दूध बच्चे को किस उम्र में देना शुरू किया?	Age of child in month उस समय बच्चे कि उम्र महीना में<br>**Ins: Code 00 if less than one month**<br>अगर एक माह से कम हो तो 00 कोड करें<br>Not yet started अभी शुरू नहीं किया है	□□<br>55	
c.	At what age did you start giving semi-solid or solid food like *khichri* to the child?<br>अर्द्ध ठोस या ठोस आहार जैसे खिचड़ी बच्चे को किस उम्र में देना शुरू किया?	Age of child in month उस समय बच्चे कि उम्र महीना में<br>**Ins: Code 00 if less than one month**<br>अगर एक माह से कम हो तो 00 कोड करें<br>Not yet started अभी शुरू नहीं किया है	□□<br>55	
H6	At what age one should start feeding the following to their child?<br>कृपया बतायें कि एक बच्चे को किस उम्र से ....खिलाना/पिलाना शुरू करना चाहिए?			
a.	Water पानी	Age of child in month बच्चे कि उम्र महीना में<br>**Ins: code 00 if less than one month**<br>अगर एक माह से कम हो तो 00 कोड़ करें<br>Do not know नहीं जानती	□□<br>88	
b.	Animal (goat, cow or buffalo) milk/ formula milk<br>जानवर (बकरी, गाय या भैंस) का दूध/फार्मूला दूध	Age of child in month बच्चे कि उम्र महीना में<br>**Ins: code 00 if less than one month**<br>अगर एक माह से कम हो तो 00 कोड़ करें<br>Do not know नहीं जानती	□□<br>88	
c.	Semi-solid or solid food like *khichri*<br>अर्द्ध ठोस या ठोस आहार जैसे खिचड़ी	Age of child in month बच्चे कि उम्र महीना में<br>**Ins: code 00 if less than one month**<br>अगर एक माह से कम हो तो 00 कोड़ करें<br>Do not know नहीं जानती	□□<br>88	

H7	What should be the consistency of food that is fed to the 6 - 24 months old children?<br><br>6 महीने से दो साल के उम्र के बच्चों को दिये जाने वाले खाने का गाढ़ापन कैसा होना चाहिए?	Thick/Solid गाढ़ा<br>Semi-solid थोड़ा गाढ़ा<br>Thin पतला<br><br>Do not Know नहीं जानती	1<br>2<br>3	
H8	How can you make a child's food nutrient and energy dense?<br><br>खाना को पोषक और शक्तिवर्धक कैसे बनाना चाहिए?	By adding butter/ghee to food<br>खाने मे मक्खन/घी मिलाकर<br>By adding sugar/jaggery to food<br>खाने मे चीनी/गुड़ मिलाकर<br>Adding green vegetables in dal<br>दाल मे हरी शब्जी मिलाकर<br>Do not Know नहीं जानती	1<br><br>2<br><br>3<br>8	

**INS: IF CHILD IS NOT ALIVE (D1J=2), GO TO I1**<br>**Check D1c. If age = 6 months or more ; then ask H9 to H10, else go to H11:**<br>निर्देशः **D1J** चेक करें। अगर बच्चा जीवित नही है तो **I1** पर जाएं<br>**D1c** से बच्चे की आयु चेक करें। यदि उम्र 6 महीने या इससे अधिक है तो **H9 – H10** पूछें, अन्यथा प्रश्न **H11** पर जायें				
H9	How many times did you feed semi-solid or solid food (Khichri /rice / roti/ halwa) to your youngest child yesterday during the day and night?<br>आपने कल दिन और रात मिलाकर कुल कितनी बार अपने सबसे छोटे बच्चे को अर्ध ठोस या ठोस पदार्थ जैसे खिचड़ी/चावल/रोटी/हलवा खिलाया था?	Number of times<br>कितनी बार	☐	
H10	Other than above, how many times did you feed snacks to your youngest child yesterday during the day and night?<br>इसके अलावा, कल का दिन और रात मिलाकर कुल कितनी बार अपने सबसे छोटे बच्चे को आपने कुछ नाश्ता खिलाया था?	Number of times<br>कितनी बार	☐	

H11 Now I would like to ask you about **liquids** drank by your youngest child yesterday during the day or at night.

INS: Multiple responses possible. Note all spontaneous answers first. Then read one by one those options which were not mentioned by the respondent.

अब आप हमें बतायें कि आप ने कल के दिन और रात मिला कर अपने सबसे छोटे बच्चे को क्या क्या पीने को दिया था ?
निर्देश : कई जवाब संभव हैं। पहले सभी सहज रूप से बताए जवाब जो माँ बताती है वह रिकार्ड करें। फिर एक एक कर के जो नहीं बताया उसके बारे में पूछें

		**Spontanious responses**<br><br>खुद से बताया	**After Probing**<br><br>पूछने पर बताया<br>**YES**	 <br><br>**NO**
**a.**	Plain water सादा पानी	1	1	2
**b.**	Milk like tinned, powdered or fresh animal milk<br>दूध जैसे जानवर का ताजा दूध/पाउडर या डिब्बा वंदी दूध	2	1	2
**c.**	Commercially produced infant formula milk<br>बाजार से मिलने वाला फार्मूला दूध	3	1	2
**d.**	Fruit juice फल का रस	4	1	2
**e.**	Any other liquids ____________________<br>कोई अन्य तरल पदार्थ	7	1	2

H12	**INS: Check D1C, if child is more than 6 months then ask H12, otherwise skip to H13**<br>Now I would like to ask you about the **food** ate by him/her yesterday during the day or at night, either separately or combined with other foods.<br>निर्देशः **D1c** से बच्चे की आयु चेक करें। यदि उम्र 6 महीने या इससे अधिक है तो **H12** पूछें, अन्यथा प्रश्न **H13** पर जायें।<br>अब मैं आप का सबसे छोटे बच्चे के द्वारा कल पुरे दिन और रात मे खाये गये खाद्य पदार्थो के बारे में पूछना चाहूँगी । हर वह पदार्थ चाहे वह जो अलग से खिलाया गया है या अन्य पदार्थो के साथ दिया गया हो या उसे बताये।

		**Yes**<br>हाँ	**No**<br>नहीं	**Do not Know**<br>नहीं जानती
a	Any baby food bought from market, e.g. Cerelac or Farex<br>बाजार से खरीदा हुआ खाने का सामान जैसे सेरेलैक या फॅरेक्	1	2	8
b	Cheese, curd, yogurt or other milk products<br>क्या आपने कल पनीर, दही या दूध से बने अन्य खाद्य पदार्थ दिये थे	1	2	8
c	Rice/Roti/Halwa/Khichri/Tehri/Biscuit<br>क्या आपने कुल दिन और रात में चावल/रोटी/हलवा/खिचड़ी/तहरी/बिस्कुट दिये थे	1	2	8
d	Daal/daal ka pani/beans/peas/nuts<br>क्या दाल/दाल का पानी/सेम/मटर/बादाम/मुम्फली/चना आदि दिया था	1	2	8
e	Pumpkin, carrots, or sweet potatoes that are yellow or orange inside<br>क्या कद्दु, गाजर, शकरकंद या मीठे आलु जो अन्दर से पीले या नारंगी रहते है दिया था	1	2	8
f	Any dark green, leafy vegetables<br>क्या कोई गहरी हरी पत्ते दार साग सब्जियां दिया था	1	2	8
g	Ripe mangoes, papayas, jackfruit<br>क्या कोई पका हुआ आम, पपीता, खरबूजा या कठहल दिया था	1	2	8
h	Any other fruits or vegetables<br>क्या कोई अन्य फल या सब्जियां दिया था	1	2	8
i	Eggs बच्चे को खाने के लिए अन्डे दिये थे	1	2	8
j	Any meat/ fish क्या कोई मांस या मछली वाला खाना दिया था	1	2	8
k	Any food made with oil, fat, ghee or butter<br>क्या तेल, घी या मक्खन से बनाया हुआ कोई भी खाना दिया था	1	2	8

IMMUNIZATION टीकाकरण

S.N.	Questions	Coding categories	Codes
H13	Do you have animmunization card where vaccinations are written down?<br>क्या आपके पास टीकाकरण कार्ड है जिसमे बच्चे को कौन कौन से टीकें लगे हैं, वह लिखे हुऐ हैं ?	Yes हाँ<br>No नहीं	1<br>2
H14	Note down all vaccine from the card or if card not available ask mother and fill in the table below<br><br>कार्ड से सभी टीकों के बारे में रिकार्ड करें। यदि कार्ड उपलब्ध नहीं है तो नीचें दी गयी टेबल को महिला से पूछ कर भरे।<br>Yes हाँ =1<br>No नहीं =2<br>Do not know नहीं जानती =8	Only by card<br>केवल कार्ड<br>Reported by mother only<br>केवल माँ बतायी<br>Both, mother and card<br>माँ और कार्ड दोनो	1<br>2<br>3

**Age of child** *(in months)* बच्चे की उम्र (महीनें में)	0-Polio 0 पोलियो	BCG which is given on left hand and leaves a scar बीसीजी जो बायें हाथ में दिया जाता है और उसका निशान रह जाता है	OPV 1 ओपीवी 1	DPT 1 डीपीटी 1	OPV 2 ओपीवी 2	DPT 2 डीपीटी 2	OPV 3 ओपीवी 3	DPT 3 डीपीटी 3	Measles which is given to a 9th month or older children मीजल्स / खसरा जो 9 महींने या उसके बाद दिया जाता है
1									
2									
3									
4-8									
9-12									

DIARRHEA डायरिया

S.N.	Questions	Coding categories	Codes	Skip to
H15	In the last 2 weeks has your youngest child had an incident of diarrhea?<br>पिछले 2 सप्ताह के अंदर क्या आपके ––– (सबसे छोटे बच्चे) को दस्त (डायरिया) हुआ था?	Yes हाँ<br>No नहीं	1<br>2 →	 I1
H16	From where you sought treatment?<br>**INS: Multiple answers possible. Code all given responses.**<br>बच्चे का इलाज किससे/कहाँ करवाया था?<br>निर्देश : कई जवाब संभव हैं। दिए गए सभी जवाब रिकार्ड करें।	ASHA/AWW आशा/आंगनवाड़ी कार्यकर्ता से<br>Government heath provider/ facilities सरकारी स्वास्थ्य कर्मी/केन्द्र<br>Private doctor/hospital प्राइवेट डाक्टर/अस्पताल<br>Unqualified village doctor गॉव के डाक्टर<br>Home treatment घरका इलाज<br>Other (specify) अन्य (स्पष्ट करे)_________<br>No treatment कोइ इलाज नहीं	1<br>2<br>3<br>4<br>5<br>7<br>9	
H17	What did you give to your youngest child to treat diarrhea?<br>**INS: Multiple answers possible. Code all given responses.**<br>आपने –– (सबसे छोटे बच्चे) को दस्त (डायरिया) ठीक करने के लिए क्या दिया?<br>निर्देश : कई जवाब संभव हैं। दिए गए सभी जवाब रिकार्ड करें।	Home made sugar and salt mix घर का बना नमक–चीनी का घोल<br>ORS ओ॰ आर॰ एस॰<br>Zinc tablets जिंक टैबलेट<br>Herbal जड़ी–बुटी<br>Other (specify) अन्य, (स्पष्ट करें)_________<br>Nothing कुछ नही	1 }<br>2 }<br>3<br>4<br>7<br>9	**H19** (1, 2)
H18	Why was sugar and salt mix/ORS not given during diarrhea?<br>दस्त (डायरिया) के दौरान बच्चे को चीनी और नमक का घोल/ ओ॰ आर॰ एस॰ क्यों नहीं दिया गया था?	I was not aware about this मुझे ओ॰आर॰एस॰/ चीनी के घोल के बारे में नहीं पता था<br>Not aware where to get ORS from मुझे पता नहीं था कि ओ॰आर॰एस॰कहां से मिलता है<br>Other (specify) अन्य, (स्पष्ट करें)_________<br>No one adviced किसी नें सलाह नहीं दी	1 }<br>2 }<br>7 }<br>9 }	**H21**
H19	Did you ask for ORS from ASHA or AWW or ANM during the last episode?<br>क्या आपने पिछले दस्त (डायरिया) के दौरान आशा या आंगनवाड़ी कार्यकर्ता या एएनएम से ओ॰ आर॰ एस॰ की मांग की?	Yes हाँ<br>No नहीं	1<br>2 →	 **H21**
H20	Did ASHA or AWW or ANM give you ORS during the last episode?<br>बच्चे के पिछले डायरिया के दौरान क्या आशा या आंगनवाड़ी कार्यकर्ता या एएनएम ने आपको ओ॰ आर॰ एस॰ पैकेंट दिया ?	Yes हाँ<br>No नहीं	1<br>2	
H21	When the child had diarrhea, was he/ she offered less than usual, about the same or more than usual **breastfeeding?**<br>जब (बच्चे का नाम) दस्त से पीड़ित था, तो क्या आपने उसे सामान्य से कम, पहले जैसा या सामान्य से अधिक स्तनपान कराया था?	Less than usual सामान्य से कम<br>Same/As usual पहले जैसा<br>More than usual सामान्य से अधिक<br>Stopped breastfeeding स्तनपान बंद कर दिया था<br>Not sure यह बताना मुश्किल है<br>No more breastfeeding अब बच्चे को स्तनपान नही कराते है	1<br>2<br>3<br>4<br>5<br>6	

S.N.	Questions	Coding categories	Codes	Skip to
H22	**INS: Check H5C. If code=55, go to I1.**<br>When (name the child) had diarrhea, was he/ she offered less than usual, about the same or more than usual to **eat?**<br>निर्देश: **H5c** चेक करें। वहां यदि कोड 55 है तो प्रश्न **I1** पर जायें।<br><br>जब (बच्चे का नाम) दस्त से पीड़ित था, तो आपने उसे कितना खाना खाने के लिए दिया था ? सामान्य से कम, पहले जैसा या सामान्य से अधिक ?	Less than usual सामान्य से कम<br>Same/As usual पहले जैसा<br>More than usual सामान्य से अधिक<br>Stopped food खाना बंद कर दिया था<br>Not sure यह बताना मुश्किल है	1<br>2<br>3<br>4<br>5	
H23	After diarrhea did you feed your baby one additional meal than usual?<br><br>दस्त के बाद क्या आपने बच्चे को सामान्य से एक अधिक बार खाना दिया?	Yes हाँ<br>No नहीं	1<br>2	

I. CONTRACEPTIVE USE

S.N.	Questions	Coding categories	Codes	Skip to
I1	Soon after marriage did you and your husband discuss on timing of first pregnancy?<br>शादी के तुरंत बाद क्या आपने और आपके पति ने इस बात पर चर्चा की थी कि पहला बच्चा कब हो?	Yes हाँ<br>No नहीं	1<br>2 →	 I3
I2	What was the decision on when to have first child?<br>**INS: Read the responses**<br><br>बच्चा कब चाहिए इसके बारे में आप लोगो का क्या निर्णय था?<br>निर्देश: कोड को पढ़ें	Both of them wanted soon<br>आप दोनों ही जल्दी चाहते थे<br>One of them wanted soon and other wanted to delay<br>आप मे से एक जल्दी और दूसरा देर करना चाहता था<br>Both of them wanted to delay<br>आप दोनों ही देर करना चाहते थे	1<br><br>2<br><br>3	
I3	Which are the days of a menstrual cycle when a woman has high risk of getting pregnant if she has unprotected sex?<br>**INS: Ask the woman to count from the day of start of menstruation**<br><br>मासिक चक्र के दौरान वे कौन से दिन हैं जिनमे यदि महिला असुरक्षित यौन संबंध बनाती है तो उसके गर्भवती होने की संभावना ज़्यादा है?<br>निर्देश : महिला से मासिक धर्म के शुरू होने के पहले दिन से गिनने के लिए कहें	☐☐ to days ☐☐<br><br>Do not know<br>नहीं जानती<br><br>Menstruation cycle मासिक चक्र<br>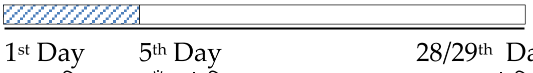<br>1st Day पहला दिन 5th Day पाँचवां दिन 28/29th Day 28वा 29वां दिन<br><br>**INS: Show the scale and ask question**<br>**निर्देश : महिला को यह रेखा दिखा कर पूछें**	 <br>88	

S.N.	Questions	Coding categories	Codes	Skip to
I4	Are you aware of emergency contraceptive pills and when it is used?<br><br>क्या आप आपातकालीन गर्भनिरोधक गोलियों के बारे मैं जानती हैं? यह गोली किस स्थिती में/क्यो इस्तेमाल करते हैं?	Not aware of ECP<br>आपातकालीन गर्भनिरोधक गोली के बारे मे नहीं जानती<br>It prevents unwanted pregnancy if no contraceptive method is used during intercourse<br>बिना गर्भनिरोधक के सम्भोग करने पर यह गर्भधारण से रोकती हैं<br>It is used to avoid pregnancy when other regular FP methods have been missed or failed<br>जब कोई नियमित परिवार नियोजन तरिके का इस्तेमाल करना भूल गया हों या वह तरीका सफल नही रहा हो तब गर्भधारण से बचने के लिए इस गोली का इस्तेमाल करते हैं<br>To introduce menstrual cycle/abortion<br>यह मासिक चक्र शुरु करने/गर्भपात मे मदद करती है<br>A new regular FP method<br>यह एक नई नियमित परिवार नियोजन विधि है<br>Other (Specify) अन्य, स्पष्ट करें __________	1<br>2<br>3<br>4<br>5<br>7	
I5	From where can one get contraceptive method like condoms, pills, ECP?<br>**INS: Multiple responses possible. Code all given responses.**<br><br>परिवार नियोजन विधियाँ जैसे कन्डोम या माला डी कहां से मिल सकती हैं?<br><br>निर्देश : कई जवाब संभव हैं। दिए गए सभी जवाब रिकार्ड करें।	ASHA आशा<br>AWW आंगनवाड़ी कार्यकर्ता<br>ANM/LHV एएनएम/एलएचवी<br>Government health facility<br>सरकारी अस्पताल<br>Private doctors /hospitals<br>प्राइवेट डाक्टर/अस्पताल<br>Medicine shop<br>दवाई कि दुकान<br>Other (Specify) अन्य, स्पष्ट करें__________<br>Do not know नहीं जानते	1<br>2<br>3<br>4<br>5<br>6<br>7<br>8	
I6	If some women want to insert IUD, from where they could get this services?<br><br>अगर किसी महिला को कॉपर–टी या आई यू डी लगवाना है तो यह उसे कहाँ से लगवा सकती है?	PHC/CHC/Dist Hospital<br>पीएचसी/सीएचसी/जिला अस्पताल<br>Private doctors<br>प्राइवेट डाक्टर<br>ANM/Sub Centre<br>एएनएम/सब सेंटर<br>Other (Specify)___________ अन्य, स्पष्ट करें<br>Do not know नहीं जानते	1<br>2<br>3<br>7<br>8	
I7	Has an ASHA or ANM ever offered you contraceptives such as condom/pills, ECP or refered you for Copper-T /IUD?<br>**INS: Multiple responses possible. Code all given responses.**<br><br>क्या आशा या ए एन एम ने कभी आपको कन्डोम या गर्भनिरोधक गोलियां, आपातकालीन गोलियां दिया या आपको कापर–टी/आईयूडी लागाने के लिए रेफर किया?<br>निर्देश : कई जवाब संभव हैं। दिए गए सभी जवाब रिकार्ड करें।	Never offered contraceptives<br>कन्डोम या गर्भनिरोधक गोलिया कभी नही दिया<br>Offered contraceptives<br>कन्डोम या गर्भनिरोधक गोलिया दिया<br>Never refered for Copper-T /IUD<br>कापर–टी/आईयूडी लागाने के लिए कभी भी रेफर नही किया<br>Refered for Copper-T /IUD<br>कापर–टी/आईयूडी लागाने के लिए रेफर किया<br>Not required contraceptives<br>जरूरत नहीं पड़ी	1<br>2<br>3<br>4<br>5	

S.N.	Questions	Coding categories	Codes	Skip to
I8	Did you ever ask for condom or pills or ECP from ASHA or ANM and did she give you?<br><br>क्या आपने आशा या एएनएम से कभी कन्डोम या गर्भ निरोधक गोलियां या आपातकालीन गोलियां मांगी और उसने आपको दिया?	Never asked<br>कभी नही मांगा<br>Asked and received<br>मांगा और मिला<br>Asked but not received<br>मांगा पर नही मिला<br>Asked and received some times<br>मांगने पर कभी कभी मिलती हैं	1<br>2<br>3<br>4	
I9	After your last delivery, did you and your husband discuss about when to have the next pregnancy or the use of FP methods?<br><br>आपके पिछले प्रसव के बाद क्या आपने और आपके पति ने अगला गर्भा धारण कब करना चाहिए या परिवार नियोजन विधियों के इस्तेमाल के बारे में कभी कोई चर्चा किया है?	Yes हाँ<br>No नहीं	1<br>2	
I10	Are you currently pregnant?<br>क्या आप अभी गर्भवती है?	Yes हाँ<br>No नहीं	1<br>2 →	 **I12**
I11	When you became pregnant this time, did you want the pregnancy at that time or wanted later or wanted no child at all?<br>इस बार जब आप गर्भवती हुई तो क्या आप उसी समय गर्भवती होना चाहती थी या बाद में चाहती थी या बिल्कुल भी नहीं चाहती थी?	Yes, wanted that time<br>हाँ उसी समय गर्भवती होना चाहती थी<br>Wanted it later<br>बाद में गर्भवती होना चाहती थी<br>Not wanted at all<br>बिल्कुल भी गर्भवती होना नहीं चाहती थी	1<br>2<br>3	J1
I12	When would you like to become pregnant again?<br><br>आप आज से कितने महीनों के बाद फिर से गर्भवती होना चाहेंगी?	As soon as possible/ within 6 months<br>जल्द से जल्द/6 महीने के अंदर<br>Between 6 months -1 year<br>6 महीने से एक साल के बीच<br>Between 1-2 years<br>1–2 साल के बीच<br>After 2 years 2 साल के बाद<br>Want no more children<br>मुझे और बच्चा नही चाहिये<br>Have not thought about it<br>इसके बारे में नहीं सोचा है<br>Other (specify)___________अन्य, स्पष्ट करें	1 →<br>2<br>3<br>4<br>5<br>6<br>7	**J1**

S.N.	Questions	Coding categories	Codes	Skip to
I13	Curently are you and/or your husband using any family planning method to delay your next pregnancy? If yes, what method?<br><br>**INS: Multiple responses possible. Code up to 3 responses.**<br>**Probe: Anything else?**<br><br>आपको अगला गर्भधारण अभी नही हो इसके लिए आप या आपके पति वर्तमान में कोई विधि इस्तेमाल कर रहे हैं?<br><br>अगर हाँ तो कौन सी विधि?<br><br>निर्देश : कई जवाब संभव। 3 जवाब तक कोड करें।<br>फिर पुछें : और क्या कर रहे हैं?	Condom कन्डोम<br>Oral Contraceptive Pills (OCP) खाने की गर्भनिरोधक गोलियां (ओसीपी)<br>Copper-T/IUD कापर–टी/आईयूडी<br>Injectable contraceptives इंजेक्शन से दिये जाने वाले गर्भनिरोधक<br>Woman has been sterilized महिला की नसबंदी हो गयी है<br>Husband has been sterilized पति की नसबंदी हो गयी है<br>Withdrawal बाहर स्खलन<br>Safe period/ Rhythm method सुरक्षित पीरियड/रिद्म वाला तरीका<br>Menstrual cycle has not started मासिक चक्र शुरू नहीं हुआ है<br>Breastfeeding स्तनपान करा रही है<br>Abstaining from sex यौन संबंध से परहेज करते हैं<br>Using LAM एलएएम/लैम विधी का इस्तेमाल<br>Other (specify)____________ अन्य, स्पष्ट करें<br>Nothing कुछ नहीं	01<br>02<br>03<br>04<br>05<br>06<br>07<br>08<br>09<br>10<br>11<br>12<br>77<br>55	01–77: J1
I14	Do you have any intention to use contraceptive method? If yes when?<br><br>क्या आप गर्भनिरोधक तरीकों का उपयोग करने के बारे में सोच रही है? अगर हाँ तो कब से शुरू करेगी?	As soon as possible जल्द से जल्द<br>Waiting for sterilization camp नसबंदी कैम्प का इंतजार कर रहे है<br>When husband agrees जब पति हाँ करे या स्वीकृती दे<br>When supply of pills is available with ASHA जब गोलियों कि सप्लाई उपलब्द हो आशा के पास तब से<br>Not sure when पता नही कब<br>Other (specify)____________ अन्य, स्पष्ट करें	1<br>2<br>3<br>4<br>5<br>7	
I15	Which methods are you interested to use?<br><br>**INS: Multiple responses possible. Code up to 2 responses.**<br><br>आप कौन सा गर्भनिरोधक तरीका आपनाने की इच्छुक है?<br><br>निर्देशः कई जवाब संभव। 2 जवाब तक कोड करें।	Condom कन्डोम<br>Oral Contraceptive Pills (OCP) खाने की गर्भनिरोधक गोलियां (ओसीपी)<br>Copper-T/IUD कापर–टी/आईयूडी<br>Injectable contraceptives इंजेक्शन से दिये जाने वाले गर्भनिरोधक<br>Female sterilization महिला नसबंदी<br>Female sterilization पूरूष नसबंदी<br>Withdrawal बाहर स्खलन<br>Safe period/ Rhythm method सुरक्षित पीरियड/रिद्म वाला तरीका<br>Abstinence यौन संबंध से परहेज<br>Other (specify)____________ अन्य, स्पष्ट करें<br>Nothing कुछ नहीं	01<br>02<br>03<br>04<br>05<br>06<br>07<br>08<br>09<br>77<br>55	

J. ACCESS TO COMMUNICATION CHANNELS AND BCM INTERVENTION

S.N.	Questions	Coding categories	Codes	Skip to
J1	**INS: Check A4, If Respondent can read, ask J1 otherwise go to J2**<br>Do you read newspaper?<br><br>क्या आप अखवार पढ़ती है?	Yes हाँ<br>No नहीं	1<br>2	
J2	Do you listen to radio?<br><br>क्या आप रेडियों सुनतीं हैं?	Yes हाँ<br>No नहीं	1<br>2	
J3	Do you watch TV?<br><br>क्या आप टेलीविजन देखती हैं?	Yes हाँ<br>No नहीं	1<br>2	
**J4-J7 are applicable only to INTERVENTION AREA, i.e GPs where health intervention is implemented**				
J4	During your last pregnancy, were you called by a SHG to attend meeting where health issues been discussed?<br><br>पिछली गर्भावस्था के दौरान क्या आपको किसी समूह में स्वास्थ्य संबंधित चर्चा में हिस्सा लेने के लिए बुलाया था?	Yes हाँ<br>No नहीं	1<br>2	
J5	During your last pregnancy, did a SHG member or SS visit your home to share knowledge on healthy practices- such as pregnancy and mother care/newborn care?<br><br>पिछली गर्भावस्था के दौरान स्वास्थ्य संबंधित चर्चा जैसे की माँ व नवजात शिशु की देखभाल कैसे करें उसको बताने के लिए क्या कोई समूह सखी या स्वास्थ्य सखी आपके घर आई थी?	Yes हाँ<br>No नहीं	1<br>2	
J6	Did you/ your family members receive any health messages through SHG during your last pregancy? If yes, who received such messages?<br><br>पिछली गर्भावस्था के दौरान आप/आपके परिवार में किसी भी सदस्य को समूह द्वारा कोई स्वास्थ्य संदेश मिला? अगर हाँ तो किस किस को यह स्वास्थ्य संदेश मिला?	Only me केवल मुझे<br>Only my other family member केवल परिवार के अन्य सदस्य को<br>Both me and family member दोनो मुझे और परिवार के अन्य सदस्य को<br>Do not know नहीं पता<br>No one किसी को नहीं	1<br>2<br>3<br>8<br>9	
J7	In last 3 months, did you receive any health related leaflet/Badhai letter from any SHG member?<br><br>पिछले 3 महिनों के दौरान क्या आपको किसी समूह के सदस्य ने स्वास्थ्य संदेश पर पर्चा या बधाई पत्र दिया था?	Yes हाँ<br>No नहीं	1<br>2	

K. SHG MEMBERSHIP

(K1 to K21 is Applicable only in GPs covered by RGMVP,i.e., *Study arm=1 or 2 and Type of HH = SHG HH*)

S.N.	Questions	Coding categories	Codes	Skip to
K1	Are you a member of an SHG?<br>क्या आप समूह की सदस्य हैं?	Yes हाँ<br>No नहीं	1<br>2	 → K5
K2	If yes, since which month and year have you been a SHG member?<br>आप कौन से साल और महीनें से समूह की सदस्य हैं?	Year वर्ष<br>Month महीना	☐☐☐☐<br>☐☐	
K3	When you expressed desire to become SHG member, did you face any of the following?<br>जब आपने समूह का सदस्य बनने की चाह बताइ तब क्या आप को घर में निम्नलिखित स्थितियों का सामना करना पडा?	(a) Oppression from MIL<br>सास का क्रोध और दबाव	Yes हाँ 1<br>No नहीं 2	
		(b) Scolding from husband<br>पति का क्रोध और डांट	Yes हाँ 1<br>No नहीं 2	
		(c) Violence or beating<br>क्रोध और मारपीट या पिटाई	Yes हाँ 1<br>No नहीं 2	
K4	**INS: If yes, (code 1 in at lease one cases of K3), ask K4 otherwise go to K5.**<br>How did they finally agree to you becoming an SHG member?<br>**INS: Multiple responses possible. Code up to 3 responses.**<br>निर्देश: अगर K3 में एक भी हाँ है तो K4 पुछें नही तो K5 में जाये<br>अंत मैं वह आपके समूह का सदस्य बनने के लिए कैसे मान गए?<br>निर्देश : कई जवाब संभव। 3 जवाब तक कोड करें।	On my persistant request<br>मेरे लगातार अनुरोध करने पर<br>Explained to them the advantages<br>समूह के सदस्य को होने वाले लाभ समझाने पर<br>Other SHG member convinced husband/MIL<br>समूह के सदस्य मिल कर सास और पति को समझाने पर<br>Other family member helped and convinced<br>परिवार के दूसरे लोगों के समझाने पर<br>Other (specify) ___________<br>अन्य (स्पष्ट करे)	1<br>2<br>3<br>4<br>7	
K5	Who all in your family are members of any RGMVP SHG?<br>**INS: Multiple responses possible. Code all given responses.**<br>आपके परिवार के और कौन कौन से लोग आर.जी.एम.वी.पी. के समूह के सदस्य है? साथ में उनके समूह का नाम भी बतायें ।<br>निर्देश : कई जवाब संभव। सभी जवाब कोड करें।	a)<br>Mother in law सास<br>Sister in law देवरानी/जेठानी/ननद<br>Other (specify) ____________ अन्य (स्पष्ट करे)<br>Noone except me<br>मेरे अलावा और कोइ नहीं	 1<br>2<br>7<br>9 → K7	b) What is the name of SHG?<br>समूह का नाम क्या हैं?<br>__________<br>__________<br>__________
K6	Since which year and month is this household is a member of the SHG?<br>आपका परिवार कौन से साल और महीने से समुह का सदस्य हैं?	Year वर्ष<br>Month महीना	☐☐☐☐<br>☐☐	
K7	Generally, how many SHG meetings take place in a month?<br>आम तौर पर एक महीने में समूह की कितनी बैठक होतीं है?	If no meeting, then code 0<br>कोई बैठक नही तो 0 कोड करें<br>Do not know नहीं पता	☐<br>8	
K8	Generally how many SHG meetings in a typical month do you/ or member of this household attend?<br>आम तौर पर एक महीने में आप कितनी बैठकों में भाग लेती है? अगर आप समुह कि सदस्य नही है तो घर का जो सदस्य समुह का सदस्य है उसके बारे में बताये?	If no meeting, then code 0<br>कोई बैठक नही तो 0 कोड करें<br>Do not know नहीं पता	☐<br>8	

S.N.	Questions	Coding categories	Codes	Skip to
K9	How many times in a typical month are health issues discussed during SHG meetings?<br>एक महीने में, समूह की कितनी बैठकों में, स्वास्थ्य सबंधी चर्चा हुई?	If no meeting, then code 0<br>कोई बैठक नही तो 0 कोड करें<br>Do not know नहीं पता	☐<br>8	
K10	What were the motivations for you or household to become the member of SHG?<br><br>**INS: Multiple responses possible. Code all given responses.**<br><br>आप का या घर के अन्य सदस्य जो समुह के सदस्य है उनको समूह का सदस्य बनने के लिए क्या प्रेरणा थी?<br><br>निर्देश : कई जवाब संभव हैं। दिए गए सभी जवाब रिकार्ड करें।	To get financial support and loan<br>आर्थिक सहायता और लोन के लिये<br>To bring economic welfare in family<br>परिवार में आर्थिक विकास के लिये<br>To have self respect in family<br>परिवार में आत्मसम्मान के लिये<br>To work outside home<br>घर से बाहर जा कर काम करने के लिए<br>To work for welfare of village women and community<br>महिलाओं और गाँव के लिए काम करने के लिए<br>Personal empowerment<br>व्यक्तिगत सशक्तिकरण/खुद की क्षमता से कुछ करने की ताकत बढाने के लिए<br>People suggested and put my name<br>लोगों ने सुझाव दिया और मेरा नाम रख दिया<br>Neighbors/known families joined<br>पडोसी/जानने वाला परिवार जुडा है इसलिये<br>To learn new things/receive knowledge informations<br>कुछ नया चीजें/नई जानकारीयाँ प्राप्त करने के लिये<br>To get opportunity to go out of home<br>घर से बाहर जाने के अवसर मिलना<br>To be independent and emporwered<br>खुद के पैरों पर खड़ें होनें के लिए<br>Other (specify) अन्य (स्पष्ट करे)_________	01<br><br>02<br><br>03<br><br>04<br><br>05<br><br>06<br><br>07<br><br>08<br><br>09<br><br>10<br><br>11<br>77	
K11	In the last 3 years, have you/your family member taken any loan from your SHG or bank (through SHG), of more than Rs.500?<br><br>क्या पिछले तीन सालों में आपने या आपके परिवार के दुसरे सदस्यों ने समूह से या अपने समूह की सहायता से बैंक से 500 या इससे ज्यादा का कर्ज लिया है?	Yes हाँ<br>No नहीं	1<br>2 →	 **K15**
K12	In the last 3 years, how many times have you/ your family member taken loan of more than Rs. 500 from your SHG?<br><br>पिछले तीन सालों में आप या आपके परिवार के दुसरे सदस्यो ने अब तक कितनी बार समूह से या अपने समूह की सहायता से बैंक से कर्ज लिया जो रू 500 या उससे ज्यादा का था?	समूह से<br><br>अपने समूह की सहायता से बैंक से<br><br>कुल	☐<br><br>☐<br><br>☐	

S.N.	Questions	Coding categories		Codes	Skip to
K13	From where (SHG/bank) did you take the loan? Start from most recent loan.<br><br>आपने कर्ज कहां से लिया था – समूह से या बैंक से ?<br><br>सबसे आखरी बार का कर्ज से बताइए।<br>SHG=1 Bank through SHG=2	B. How much loan was taken each time? Write loan amount in Rs.<br>कितना कर्ज लिया था?<br>कर्ज का राशि रूपये में लिखें	C. What was the **main** purpose of the loan?*<br>कर्ज लेने का <u>मुख्य कारण क्या</u> था?		
	**A) Last loan** ☐	☐☐☐☐☐	☐☐		
	**B) 2nd from last loan** ☐	☐☐☐☐☐	☐☐		
	**C) 3rd from last loan** ☐	☐☐☐☐☐	☐☐		
	**Purpose***<br>01=To start business व्यापार शुरु करने के लिए<br>02=To expand business<br>व्यापार बडा करने करने के लिए<br>03= To pay back old loan/loan swapping<br>पिछले लोन का भुगतान करने के लिए<br>04 =Treatment of illness रोग का इलाज<br>05 =Delivery expenses प्रसव के खर्चे<br>06=Marriage expenses शादी के खर्चे<br>07=Education of children बच्चों की शिक्षा<br>08=House repairing/construction घर की मरम्मत/बनवाना<br>77=Other (specify) अन्य (स्पष्ट करे) ___________				
K14	**INS: Check K13, if Purpose of loan taken includes codes 1 & 2 continue with K14 otherwise skip to K15**<br><br>**Mainly**, for what business was the loan used?<br><br>निर्देशः **K13** के जवाब को देखें अगर कोड़ 1 और 2 दिया है तो **K14** का सवाल करें नही तो **K5** पर जाँय<br><br>आप ने कर्ज का उपयोग <u>मुख्य रूप से</u> किस व्यापार के लिए किया?	Agriculture/ farm releated activities with sale of product<br>कृषि या उस से संबधित काम, जिसके पैदवार को आप बेच सकते हो<br>Goat and cattle rearing/sale<br>पशु की खरीद व पालना व बेचना<br>Fisheries मछली पालन<br>Shop provision/grocery sell<br>किराने की दुकान या उसी तरह की अन्य दुकान<br>Purchase of swing machine for tailoring<br>सिलाई मशीन खरीदकार सिलाई का काम करने के लिये<br>Making handicarft घरेलु उधोग करने के लिय<br>Other (specify)अन्य (स्पष्ट करे)__________		1<br><br>2<br><br>3<br>4<br><br>5<br>6<br>7	
K15	In the last 3 months have you or any SHG member of your family participated in discussion within your SHG on identification of pregnant women/recently delivered women/women with less than 2 years child?<br>पिछले 3 महिनों के दौरान क्या आप या आपके परिवार में जो समूह के सदस्य हें उन्होनें अपने समूह के बैठकों में भाग लिया, जिसमें गर्भवती महिला या जिस के हाल में ही (42 दिन के अन्दर) बच्चा हुआं हैं या ऐसी महिला जिसका 2 साल से छोटा बच्चा हो उसको पहचान करने की चर्चा हुई?	Yes हाँ<br>No नहीं<br>Don't Know पता नही या मालूम नही		1<br>2<br>8	

S.N.	Questions	Coding categories	Codes	Skip to
K16	In the last 3 months have you or any SHG member of your family been attached as sakhi to any pregnant women/ recently delivered women/women with less than 2 years child?<br>पिछले 3 महिनों के दौरान क्या आप या आपके परिवार में जो समूह के सदस्य हें उन्होनें किसी गर्भवती महिला या ऐसी महिला जिसको हाल में बच्चा हुआ हो या जिसका 2 साल से छोटा बच्चा हो उन के साथ जुड़े हैं?	Yes हाँ<br>No नहीं<br>Don't Know पता नही या मालूम नही	1<br>2<br>8	
K17	In last 3 months have you or any SHG member of your family visited any pregnant women/women with less than 2 year child households to distribute shubhkamna /Badhai letter?<br>पिछले 3 महिनों के दौरान क्या आप या आपके परिवार में जो समुह के सदस्य है उन्होने किसी गर्भवती महिला या जिसको हाल में बच्चा हुआ हो या ऐसी महिला जिसका 2 साल से छोटा बच्चा हो उस को शुभकामना/बधाई पत्र दिया है?	Yes हाँ<br>No नहीं<br>Don't Know पता नही या मालूम नही	1<br>2<br>8	
K18	In last 3 months have you or any SHG member of your family ever asked for supply like ORS, IFA, family planning products, supplementary nutrition etc from Government health workers (ASHA/ANM/doctors) or AWW?<br>पिछले 3 महिनों के दौरान क्या आपनें या आपके परिवार में जो समुह के सदस्य हे उन्होनें कभी सरकारी स्वास्थकर्मी (आशा/ए ॰एन॰एम॰ /डाक्टर) या आंगनवाड़ी कार्यकर्ता से ओ॰ आर॰ एस॰/आई॰एफ॰ ए॰/परिवार नियोजन की विधी या पूरक पोषाहार मांगा?	Yes हाँ<br>No नहीं<br>Don't Know पता नही या मालूम नही	1<br>2<br>8	
K19	Did you or any SHG member of your family talk within family about the health issues that were discussed in the SHG meetings?<br>समूह की बैठकों में चर्चा किए हुए स्वस्थ सबंधी मुद्दों पर क्या आपने, या आप के घर के सदस्य जो समूह के सदस्य है उन्होंने उनपर अपने परिवार के अन्य सदस्यों के साथ बातचीत किया?	Yes हाँ<br>No नहीं	1<br>2	
K20	Did you or any SHG member of your family talk with your neighbor or other non-SHG household members about the health issues discussed in the SHG meetings?<br>क्या आप या आप के घर के सदस्य जो समूह के सदस्य है समुह में होने वाले स्वास्थ्य सबंधी बातों की चर्चा, अपने पड़ोसी या अन्य परिवार कि महिला जो समूह से नहीं जुडी है उनके साथ बातचीत किया ?	Yes हाँ<br>No नहीं<br>Don't Know पता नही या मालूम नही	1<br>2 }<br>8 }	 <br>L1
K21	Do all the people, with whom you or any SHG member of your family discussed the health issues, live in this purva/village or some of them live in other purva/villages?<br>जिनके साथ यह चर्चा हुई क्या वह सब इसी गाँव या मोहल्ले में रहते है या बाहर रहते है?	All live in the same purva/village सभी लोग इस गॉव/महल्ले में रहते है<br>Some live in this purva and some in other village/purva उनमे से कुछ लोग अन्य गॉव/मोहल्ले में रहते है<br>All live in other purva/village सभी लोग अन्य गॉव/मोहल्ले में रहते है<br>Don't Know पता नही या मालूम नहीं	1<br>2<br>3<br>8	L1

S.N.	Questions	Coding categories	Codes	Skip to
**APPLICABLE to WOMEN from NON-SHG household only (NSHG1 and NSHG2)**				
K22	In the last 3 years, have you or your family member taken any loans, which was more than Rs. 500?<br>क्या पिछले तीन सालों में आपने या आपके परिवार के दुसरे सदस्यों ने रू 500 से ज्यादा कोई कर्ज लिया है?	Yes<br>No<br>Don't Know	1<br>2<br>8	L1 (codes 2, 8)
K23	In the last 3 years, how many times have you/ your family member taken loan which was Rs. 500 or more?<br><br>पिछले तीन सालों में आपने या आपके परिवार के दुसरे सदस्यों ने रू 500 से ज़्यादा कितनी बार कर्ज लिया है?		☐	
K24	From where did you take loan each time? Start from the most recent one.<br>हर बार आपने कर्ज कहां से लिया था? सबसे आखरी बार का कर्ज से बताइए<br>Friends/neighbors दोस्त/पडोसी = 1<br>Family परिवार = 2<br>Money lender साहूकार = 3<br>Bank (not through SHG) बैंक से (बिना समुह के) = 4	B) How much loan was taken each time?<br>Write loan amount in Rs.<br><br>हर बार कितना कर्ज लिया था?<br><br>कर्ज राशि रूपये में लिखें	C) What was the main purpose of the loan?*<br><br>कर्ज लेने का मुख्य कारण क्या था?	
	**A) Last loan** ☐	☐☐☐☐☐	☐☐	
	**B) 2nd from last loan** ☐	☐☐☐☐☐	☐☐	
	**C) 3rd from last loan** ☐	☐☐☐☐☐	☐☐	
K25	**INS: Chek K24, ask only if code is 3 or 4 (money lender or bank) in anyone of the sources of loan.**<br>At what interest rate was the loan taken?<br><br>निर्देश: तभी पुछें जब कर्ज सहूकार या बैंक से लिया हो।<br>आप ने कर्ज किस ब्याज दर पर लिया ?	**Moneylender** साहूकार ☐☐.☐☐<br><br>**Bank** बैंक ☐☐.☐☐		

Purpose*

01=To start business व्यापार शुरु करने के लिए
02=To expand business व्यापार बडा करने करने के लिए
03= To pay back old loan/loan swapping पिछले लोन का भुगतान करने के लिये
04 =Treatment of illness रोग का इलाज के लिय
05 =Delivery expenses प्रसव के खर्चे
06=Marriage expenses शादी के खर्चे
07=Education of children बच्चों की शिक्षा
08=House repairing/construction घर की मरम्मत/ बनवाना
77=Other (specify) अन्य (स्पष्ट करे) ___________

L. EMPOWERMENT

S.N.	Questions	Coding categories	Codes
L1.	I am going to read out some statements about events. On these events can you speak out your mind/say what you feel: **(SELF-EXPRESSION)**<br>**INS: Read the option with every statement**<br><br>मैं आपको कुछ परिस्थितीयों के बारे में पढ़कर सुनाती हूँ। क्या आप इन परिस्थितीयों में बिना किसी हिचक के अपने मन की बात कह सकतीं हैं?<br><br>निर्देशः हर कोड को हर किसी सवाल के साथ पढ़ें	Most of the time/ हमेशा=1<br>Occasionally/कभी कभी= 2<br>Never/कभी नहीं= 3<br><br>**INS: Read codes every time**<br><br>निर्देशः सभी कोड हर बार पढ़ कर सुनाये	
	a) In any meeting or in any training or SHG meeting<br>लोगों की बैठक में हों या ट्रेनिंग के दौरान या समूह में, क्या आप बिना किसी हिचक के अपने मन की बात कह सकतीं हैं? ऐसा हमेशा होता है, कभी कभी होता है या कभी नहीं होता है?	हमेशा<br>कभी कभी<br>कभी नहीं	1<br>2<br>3
	b) With a bank officer/manager<br>अगर आपको बैंक अफसर के साथ बात करनी हो, तो क्या आप बिना किसी हिचक के अपने मन की बात कह सकतीं हैं? ऐसा हमेशा होता है, कभी कभी होता है या कभी नही होता है?	हमेशा<br>कभी कभी<br>कभी नहीं	1<br>2<br>3
	c) With the Sarpanch /pradhan<br>सरपंच/प्रधान के साथ बात करने कि आवश्यक्ता हो तो क्या आप बिना किसी हिचक के अपने मन की बात कह सकतीं हैं? ऐसा हमेशा होता है, कभी कभी होता है या कभी नही होता है?	हमेशा<br>कभी कभी<br>कभी नहीं	1<br>2<br>3
	d) With the ANM/AWW/ASHA<br>एएनएम, आंगनवाड़ी कार्यकर्ता या आशा के साथ बात करने में क्या आप बिना किसी हिचक के अपने मन की बात कह सकतीं हैं? ऐसा हमेशा होता है, कभी कभी होता है या कभी नही होता है?	हमेशा<br>कभी कभी<br>कभी नहीं	1<br>2<br>3
L2.	Can you say that you have no restriction from your family in going alone outside home for the following purposes: **(MOBILITY)**<br>**INS: Read the option with every statement**<br><br>अगर किसी काम से आपको घर के बाहर अकेले जाना हो तो क्या आप घर के किसी सदस्य को केवल बता कर, बिना किसी भय के अकेले बाहर जा सकतीं हैं? उदाहरण के तौर पर:<br>निर्देशः हर कोड को हर किसी सवाल के साथ पढ़े	Most of the time/ हमेशा=1<br>Occasionally/कभी कभी= 2<br>Never/कभी नहीं= 3<br><br>**INS: Read codes every time**	
	a) Shopping within the village:<br>अगर गाँव में खरीदारी करने के लिए जाना हो, तो क्या आप घर के किसी सदस्य को केवल बता कर, बिना किसी भय के अकेले बाहर जा सकतीं हैं? ऐसा हमेशा होता है, कभी कभी होता है या कभी नही होता है?	हमेशा<br>कभी कभी<br>कभी नहीं	1<br>2<br>3
	b) Shopping outside the village<br>अगर खरीदारी करने के लिए गाँव के बाहर जाना हो, तो क्या आप घर के किसी सदस्य को केवल बता कर, बिना किसी भय के अकेले बाहर जा सकतीं हैं? ऐसा हमेशा होता है, कभी कभी होता है या कभी नही होता है?	हमेशा<br>कभी कभी<br>कभी नहीं	1<br>2<br>3
	c) Visiting health facility/doctor outside village<br>अगर गाँव के बाहर किसी स्वास्थ्य केंद्र या डाक्टर के पास जाना हो, तो क्या आप घर के किसी सदस्य को केवल बता कर, बिना किसी भय के अकेले बाहर जा सकतीं हैं? ऐसा हमेशा होता है, कभी कभी होता है या कभी नही होता है?	हमेशा<br>कभी कभी<br>कभी नहीं	1<br>2<br>3
	d) Going to VHSND/immunization camp within the village<br>अगर गाँव के अंदर टीकाकरण दिवसए जो एएनएम दीदी करती हैं उस में जाना हो, तो क्या आप बिना किसी रोक टोक जा सकतीं हैं? ऐसा हमेशा होता है, कभी कभी होता है या कभी नही होता है?	हमेशा<br>कभी कभी<br>कभी नहीं	1<br>2<br>3
	e) Attending a religious festival/social function within the village<br>अगर गाँव के अंदर धार्मिक व सामाजिक पर्व या मिलन पर जाना हो, तो क्या आप बिना किसी रोक टोक के अकेले जा सकतीं हैं? ऐसा हमेशा होता है, कभी कभी होता है या कभी नही होता है?	हमेशा<br>कभी कभी<br>कभी नहीं	1<br>2<br>3

<table>
<tr><td>L3.</td><td>Within the family, generally you are consulted if a decision on the following issues is taken: (DECISION MAKING)
INS: Read the option with every statement

यदि परिवार में कुछ फैसले लेने होते हैं तो आपकी राय भी ली जाती हैं जैसे
निर्देश: हर कोड को हर किसी सवाल के साथ पढ़ें</td><td colspan="2">Most of the time/हमेशा= 1
Occasionally/कभी कभी = 2
Never/कभी नहीं = 3

INS: Read codes every time</td></tr>
<tr><td></td><td>a) Repair/extension of the house
घर की मरम्मत करवाना या एक–आध कमरा बढ़ाने के फैसले के लिए आपकी राय भी ली जाती हैं। ऐसा हमेशा होता है, कभी कभी होता है या कभी नही होता है?</td><td>हमेशा
कभी कभी
कभी नहीं</td><td>1
2
3</td></tr>
<tr><td></td><td>b) Purchase of livestock
गाय, भैंस या बकरी खरीदने का फैसला करने से पहले आपकी राय भी ली जाती हैं। ऐसा हमेशा होता है, कभी कभी होता है या कभी नही होता है?</td><td>हमेशा
कभी कभी
कभी नहीं</td><td>1
2
3</td></tr>
<tr><td></td><td>c) Investment in business/land
जमीन खरीदना या किसी धंधे में पैसे लगाने का फैसला करने से पहले आपकी राय भी ली जाती हैं। ऐसा हमेशा होता है, कभी कभी होता है या कभी नही होता है?</td><td>हमेशा
कभी कभी
कभी नहीं</td><td>1
2
3</td></tr>
<tr><td></td><td>d) Choice of doctor for child's treatment
बच्चे के इलाज के लिय किस डाक्टर को चुने इस का फैसले लेने से पहले आपकी राय भी ली जाती हैं। ऐसा हमेशा होता है, कभी कभी होता है या कभी नही होता है?</td><td>हमेशा
कभी कभी
कभी नहीं</td><td>1
2
3</td></tr>
<tr><td></td><td>e) Taking loan from Bank/SHG/moneylender/other source
बैंक या समूह, साहूकार या कहीं और से उधार लेने का फैसला करने से पहले आपकी राय भी ली जाती हैं। ऐसा हमेशा होता है, कभी कभी होता है या कभी नही होता है?</td><td>हमेशा
कभी कभी
कभी नहीं</td><td>1
2
3</td></tr>
<tr><td>L4.</td><td>I am going to read a number of statements. Indicate whether it is true; most of the time, occasionally or never.
In general you make the decision on the following aspects:
(CONTROL OF RESOURCES)
INS: Read the option with every statement
मै आप को कुछ वाक्य पढ़कर सुनाती हूं, आप बताये यह वाक्य किस हद तक आप पर लागु होते हैं। क्या यह फैसले आप खुद करती है।. हमेशा या कभी कभी या कभी नहीं?
निर्देश: हर कोड को हर किसी सवाल के साथ पढ़ें</td><td colspan="2">Most of the time/हमेशा = 1
Occasionally/कभी कभी = 2
Never/कभी नहीं = 3

INS: Read codes every time</td></tr>
<tr><td></td><td>a) Whether or not to work for money
पैसे के लिए आप काम करें या न करें इस का फैसला आप खुद लेती हैं। ऐसा हमेशा होता है, कभी कभी होता है या कभी नही होता है?</td><td>हमेशा
कभी कभी
कभी नहीं</td><td>1
2
3</td></tr>
<tr><td></td><td>b) What livelihood activity you should take-up
आमंदनी के लिऐ कौनसा काम या धंधा करना चाहिए इस का फैसला आप खुद लेती है। ऐसा हमेशा होता है, कभी कभी होता है या कभी नही होता है?</td><td>हमेशा
कभी कभी
कभी नहीं</td><td>1
2
3</td></tr>
<tr><td></td><td>c) Money that could be spent on education of children
बच्चों की पढाई पर कितने पैसे खर्च किए जाएं इस का फैसला आप खुद लेती है। आप किस हद तक सहमत है? ऐसा हमेशा होता है, कभी कभी होता है या कभी नही होता है?</td><td>हमेशा
कभी कभी
कभी नहीं</td><td>1
2
3</td></tr>
<tr><td></td><td>d) When you should have next child
यह अधिकतर आपका निर्णय है कि अगला बच्चा कब हो। आप किस हद तक सहमत है? ऐसा हमेशा होता है, कभी कभी होता है या कभी नही होता है?</td><td>हमेशा
कभी कभी
कभी नहीं</td><td>1
2
3</td></tr>
</table>

L5.	Have you taken any action along with SHG members or community members to demand any of the following **(COLLECTIVE ACTION)**<br>**INS: Read the option with every statement**<br>निर्देशः कोड को हर किसी सवाल के साथ पढ़ें<br><br>पिछले एक साल में आपने गाँव की और महिलाओं के साथ या समूह के साथ मिलजुल कर अपने गाँव वालों के लिए क्या सरकार या पंचायत से कोई मांगे की जैसे?	Yes/हाँ = 1<br>No/ नहीं = 2<br><br>**INS: Read code every time**	
	a) Demand for better health services for mother and child in the community/VHND/health facility or from AAA.<br>स्वास्थ्य केन्द्र या एएनएम आंगनवाड़ी कार्यकर्त्री आशा से माँ और बच्चे के लिए बेहतर स्वास्थ्य सेवा की मांगें की?	हाँ<br>नहीं	1<br>2
	b) Demand for BPL card<br>गरीबी रेखा या बी पी एल (BPL) के कार्ड की मांग सरकार या पंचायत से की हैं?	हाँ<br>नहीं	1<br>2
	c) Demand for MNREGA<br>मनरेगा में काम करने की मांग सरकार या पंचायत से की हैं?	हाँ<br>नहीं	1<br>2
	d) Demand for PDS/ration card<br>सरकार या पंचायत से राशन के कार्ड की मांग की?	हाँ<br>नहीं	1<br>2
	e) Demand for opening bank account<br>बैंक में खाता खुलवाने की मांग की हैं?	हाँ<br>नहीं	1<br>2
	f) Any other (specify) कोई और मांग? (स्पष्ट करें)	हाँ<br>नहीं	1<br>2
L6.	Please tell me to what extent you agree or disagree to the following statements: **(SELF ESTEEM)**<br>**INS: Read the option with every statement**<br>मैं आप को अब कुछ वाक्य पढ़कर सुनाती हूं, सोच कर बताऐं यह आप पर किस हद तक लागू होते हैं।<br>निर्देशः कोड को हर किसी सवाल के साथ पढ़ें	Agree पूरी तरह = 1<br>Agree to some extent<br>कुछ हद तक = 2<br>Disagree बिलकुल नहीं = 3<br><br>**INS: Read codes every time**	
	a) I can do a work as efficiently as any other person can do<br>मैं कोई भी काम उतनी ही कुशलता और आसानी से कर सकती हूँ जितना कोई और महिला कर सकती है। आप इस से किस हद तक सहमत है?<br>पूरी तरह, कुछ हद तक या बिलकुल नही?	पूरी तरह<br>कुछ हद तक<br>बिलकुल नहीं	1<br>2<br>3
	b) I feel I have spent a wasteful life. I have had no achievement in life.<br>मुझे लगता है कि मेरा जीवन बेकार गुजर गया। मुझे जीवन में कोई सफलता नही मिली है। आप इस से किस हद तक सहमत है?<br>पूरी तरह, कुछ हद तक या बिलकुल नही?	पूरी तरह<br>कुछ हद तक<br>बिलकुल नहीं	1<br>2<br>3
	c) I have no respect either in the family or in the community<br>ना तो मुझे घर में सम्मान दिया जाता हैए ना ही घर के बाहर।<br>पूरी तरह, कुछ हद तक या बिलकुल नहीं?	पूरी तरह<br>कुछ हद तक<br>बिलकुल नहीं	1<br>2<br>3
	d) I feel satisfied that I am consulted in most family decisions<br>मुझे बुरा लगता है कि घर में अगर कोई अहम् या ज़रूरी फैसला लिया जाता है तो हमरी राय नहीं ली जाती हैं। आप इस से किस हद तक सहमत है?<br>पूरी तरह, कुछ हद तक या बिलकुल नहीं?	पूरी तरह<br>कुछ हद तक<br>बिलकुल नहीं	1<br>2<br>3
	e) When I visit clinics, I feel I am not given equal respect as anybody else there<br>जब मैं सवास्थ्य केंद्र जाती हूँ तो मुझे लगता है कि मुझे उतना सम्मान नहीं मिलती है जितना वहाँ और महिलाओं को मिलती है। आप इस से किस हद तक सहमत है?<br>पूरी तरह, कुछ हद तक या बिलकुल नहीं?	पूरी तरह<br>कुछ हद तक<br>बिलकुल नहीं	1<br>2<br>3
	f) I don't feel there is any difference between me and a woman of another caste<br>मुझे नहीं लगता कि मुझमें या किसी और अन्य जाति कि महिला में कोई अंतर है। आप इस से किस हद तक सहमत है?<br>पूरी तरह, कुछ हद तक या बिलकुल नहीं?	पूरी तरह<br>कुछ हद तक<br>बिलकुल नहीं	1<br>2<br>3

M. DOMESTIC VIOLENCE

M1.	**(SELF ESTEEM Contd.)**<br>A wife beating is justified if:<br>**INS:** Read the option with every statement<br>पत्नी को पति का मारना पीटना ठीक है अगर<br>निर्देश: हर कोड को हर किसी सवाल के साथ पढ़ें	Agree/पूरी तरह = 1<br>Agree to some extent/ कुछ हद तक = 2<br>Disagree/बिलकुल नहीं = 3<br>**INS: Read code every time**	
	a) She burns the food<br>वह खाना जला देती है। तो क्या पति का मारना पीटना किस हद तक ठीक है? पूरी तरह, कुछ हद तक या बिलकुल नहीं?	पूरी तरह<br>कुछ हद तक<br>बिलकुल नहीं	1<br>2<br>3
	b) She neglects the children<br>वह बच्चों पर ध्यान नहीं देती है। तब पति का मारना पीटना किस हद तक ठीक है? पूरी तरह, कुछ हद तक या बिलकुल नहीं?	पूरी तरह<br>कुछ हद तक<br>बिलकुल नहीं	1<br>2<br>3
	c) She argues with husband<br>वह पति से बहस करती है। तब पति का मारना पीटना किस हद तक ठीक है? पूरी तरह, कुछ हद तक या बिलकुल नहीं?	पूरी तरह<br>कुछ हद तक<br>बिलकुल नहीं	1<br>2<br>3
	d) She talks to other men<br>वह पराये पुरूष से बात करती है। तब पति का मारना पीटना किस हद तक ठीक है? पूरी तरह, कुछ हद तक या बिलकुल नहीं?	पूरी तरह<br>कुछ हद तक<br>बिलकुल नहीं	1<br>2<br>3
	e) She wastes husbands money<br>वह पति के पैसे बर्बाद करती है। तब पति का मारना पीटना किस हद तक ठीक है? पूरी तरह, कुछ हद तक या बिलकुल नहीं?	पूरी तरह<br>कुछ हद तक<br>बिलकुल नहीं	1<br>2<br>3
	f) She goes out without telling husband<br>वह पति को बिना बताए घर के बाहर जाती है। तब पति का मारना पीटना किस हद तक ठीक है? पूरी तरह, कुछ हद तक या बिलकुल नहीं?	पूरी तरह<br>कुछ हद तक<br>बिलकुल नहीं	1<br>2<br>3
	g) She refuses sex to husband<br>वह पति के साथ यौन संबंध बनाने से मना करती है। तब पति का मारना पीटना किस हद तक ठीक है? पूरी तरह, कुछ हद तक या बिलकुल नहीं?	पूरी तरह<br>कुछ हद तक<br>बिलकुल नहीं	1<br>2<br>3
M2.	Now I am going to read few happening whichoften occurs in family. I will like you to tell whther during the last 12 months or ever your husband has done any of the following<br>**INS:** Read the option with every statement<br>अब में कुछ वाक्य पढकर सुनाती हु कृपया बताँये की पिछले 12 महिनों में आप के पति ने कभी ऐसा किया? अगर पिछले 12 महिनों में नही तो क्या उन्होंने ऐसा कभी भी ऐसा किया?	**(A)**<br>**Last 12 months**<br>पिछले 12 महिनों में	**(B)**<br>**Ever**<br>कभी भी
	a) Said or done something to humiliate you in front of others?<br>दूसरों के सामनें आपकों कुछ कहा या बोला जिस से आपका अपमान हुआ हो?	Yes हाँ 1<br>No नहीं 2 →	Yes हाँ 1<br>No नहीं 2
	b) Insulted you or made you feel bad about yourself?<br>आपको ऐसी बातें कहीं हो जिस से आप खुद को तुच्छ महसूस करने लगे हों?	Yes हाँ 1<br>No नहीं 2 →	Yes हाँ 1<br>No नहीं 2
	c) Slapped you?<br>आपको झापड़ लगाया हो?	Yes हाँ 1<br>No नहीं 2 →	Yes हाँ 1<br>No नहीं 2
	d) Twisted your arm or pulled your hair?<br>आपकी बाजू या हाथ मोड़ा हो या बाल खीचा हो?	Yes हाँ 1<br>No नहीं 2 →	Yes हाँ 1<br>No नहीं 2
	e) Punched you or serious beating?<br>आपकों घूंसा या जोरदार पिटाई की हो?	Yes हाँ 1<br>No नहीं 2 →	Yes हाँ 1<br>No नहीं 2
	f) Had sexual intercourse with you when you did not want to because you were afraid of what your husband might do? आपके साथ संभोग किया जब कि आप नही चाहती थी क्योकि आप को डर था कि न जाने आपके पति क्या करेंगे?	Yes हाँ 1<br>No नहीं 2 →	Yes हाँ 1<br>No नहीं 2
	g) Physically forced you to have sexual intercourse with him even when you did not want to?<br>आपके नही चाहते हुए भी आपके साथ जबरदस्ती संभोंग किया?	Yes हाँ 1<br>No नहीं 2 →	Yes हाँ 1<br>No नहीं 2
	h) Forced you to perform any sexual acts that you did not want to?<br>आपको जबरदस्ती यौन लिंग सबंधी कुछ ऐसे काम करवाये जो आप नही करना चाहती थी ?	Yes हाँ 1<br>No नहीं 2 →	Yes हाँ 1<br>No नहीं 2
	i) Threatened to marry other women<br>अन्य महिला को शादी करने से धमकी दी	Yes हाँ 1<br>No नहीं 2 →	Yes हाँ 1<br>No नहीं 2

M3.	I would like to read out some statements. I would like you to think about your relationship with your husband, and for each statement to tell me how strongly you agree.<br>**INS:** Read the option with every statement<br>मैं अब आपको कुछ परिस्थीतियों के बारे में पढ़ कर सुनाती हू। आप इन से किस हद तक सहमत है?<br>निर्देश: हर कोड को हर किसी सवाल के साथ पढ़ें	Agree/पूरी तरह = 1<br>Agree to some extent/ कुछ हद तक = 2<br>Disagree/बिलकुल नहीं = 3<br><br>**INS: Read code every time**	
a)	When my husband wants sex he expects me to agree<br>जब मेरे पति को यौन सबंध बनाने होते है तो वह उम्मीद रखते है कि में सहमत होंगी।	पूरी तरह<br>कुछ हद तक<br>बिलकुल नहीं	1<br>2<br>3
b)	If I asked my husband to use a condom, he would get angry<br>यदि मैं अपने पति से कोन्ड्म का उपयोग करने को कहू तो वह गुस्सा होगे।	पूरी तरह<br>कुछ हद तक<br>बिलकुल नहीं	1<br>2<br>3
c)	I cannot take any initiative to ask my husband for family planning as he will think that I do not have a good moral character<br>मै किसी भी गर्भनिरोधक का उपयोग करने की राय खुद से नहीं कर सकती, क्योकि मेंरे पति सोचेगा की मेरा नैतिक चरित्र अच्छा नहीं है।	पूरी तरह<br>कुछ हद तक<br>बिलकुल नहीं	1<br>2<br>3
d)	I think my husband is having sex with some other woman also<br>मेरे पति किसी और महिला के साथ भी यौन सबंध बनाते हैं	पूरी तरह<br>कुछ हद तक<br>बिलकुल नहीं	1<br>2<br>3
e)	I know that my husband frequently drinks (take alcohol)<br>मुझे पता है कि मेंरे पति अक्सर शराब पीते है	पूरी तरह<br>कुछ हद तक<br>बिलकुल नहीं	1<br>2<br>3

Thank You
धन्यवाद

End Time ☐☐ ☐☐
समाप्त करने का समय
